# The RASSF1C-HIF-1α axis drives macrophage lipid metabolism to promote pancreatic cancer

**DOI:** 10.1038/s41419-026-08609-0

**Published:** 2026-03-30

**Authors:** Ting Zhan, Min Huang, Mengge Chen, Wei Chen, Yiyun Wang, Xiaoli Chen, Yanli Zou, Meng Liu, Qingxi Zhu, Xia Tian, Zheng Han, Xiaodong Huang

**Affiliations:** 1 Department of Gastroenterology, Tongren hospital of WuHan University(WuHan Third Hospital), Wuhan, China; 2 Department of Gastroenterology, Zhongnan Hospital of Wuhan University, Wuhan, China

**Keywords:** Cancer metabolism, Post-translational modifications

## Abstract

Pancreatic adenocarcinoma (PAAD) has a poor prognosis. Its microenvironment is closely associated with tumor progression and immune evasion. This study combines single-cell RNA sequencing (scRNA-seq) and spatial transcriptomics (ST) to reveal the critical role of tumor-associated macrophages (TAMs) in PAAD. Ras association domain family member 1 C (RASSF1C) is significantly upregulated under hypoxia, enhancing glycolysis by promoting the Warburg effect. This generates lactate and contributes to acidification of the tumor microenvironment (TME). Lactate activates TAMs and reprograms their lipid metabolism, promoting PAAD migration and invasion. Further investigation demonstrated that lactate suppressed ubiquitin-fold modifier 1 ligating enzyme 1 (UFL1) protein levels in macrophages, thereby weakening the protective effect of UFL1-mediated interferon regulatory factor 7 (IRF7) UFMylation. This suppression led to enhanced K48-linked ubiquitination of IRF7 and accelerated proteasomal degradation, ultimately reducing IRF7 stability and impairing lipid metabolic functions in macrophages. Additional mechanistic evidence showed that UFL1-UFMylation axis maintains IRF7 homeostasis by counteracting K48-linked ubiquitin-mediated degradation. Moreover, immunohistochemical (IHC) validation using tissue microarrays from 20 human pancreatic ductal adenocarcinoma (PDAC) specimens revealed that the overall expression of RASSF1C and hypoxia-inducible factor-1 alpha (HIF-1α) was higher than that of UFL1 and IRF7. RASSF1C expression was significantly positively correlated with HIF-1α and negatively correlated with UFL1 and IRF7. Clinicopathological correlation analysis further showed that high RASSF1C expression was associated with poor differentiation and advanced TNM stage, whereas low UFL1 and IRF7 expression was associated with lymph node metastasis. Collectively, this study demonstrated that the hypoxia-RASSF1C-HIF-1α axis reshaped TAM function through lactate-mediated immunometabolic regulation and promoted PAAD progression by inhibiting UFL1-mediated IRF7 UFMylation, thereby reducing IRF7 stability. These findings identify potential therapeutic targets for combined metabolic and immune interventions in PAAD.

**Figure:**
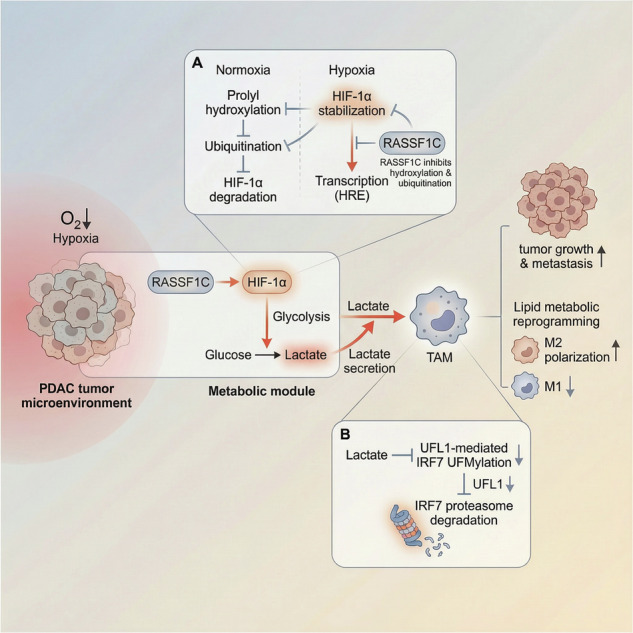
Graphic abstract. Schematic illustration of the molecular mechanism by which the RASSF1C-HIF-1α axis induces glycolytic reprogramming, drives lactate accumulation in the acidic TME, and promotes lactate flux into macrophages, thereby facilitating UFL1-IRF7 interaction and ultimately accelerating PAAD progression.

Graphic abstract. Schematic illustration of the molecular mechanism by which the RASSF1C-HIF-1α axis induces glycolytic reprogramming, drives lactate accumulation in the acidic TME, and promotes lactate flux into macrophages, thereby facilitating UFL1-IRF7 interaction and ultimately accelerating PAAD progression.

## Introduction

Pancreatic adenocarcinoma (PAAD) is a highly malignant tumor characterized by rapid progression and a high propensity for metastasis, making it one of the leading causes of cancer-related deaths worldwide [1, 2]. According to the World Health Organization, the five-year survival rate for PAAD is only about 9%, primarily due to late diagnosis, the lack of early symptoms and effective screening methods, and its high resistance to current treatment options [3, 4]. For this type of cancer, radiotherapy and chemotherapy are critical treatment modalities. Although these therapies can be effective in some cases, further research is needed to improve their efficacy and reduce side effects [5, 6]. Therefore, there is an urgent need to develop new diagnostic and therapeutic strategies to improve patient survival and prognosis.

The tumor microenvironment (TME) refers to the complex network of non-cancerous cells, extracellular matrix (ECM), blood vessels, and immune cells within tumor tissue [7, 8]. Immune cells, particularly tumor-associated macrophages (TAMs), play a crucial role in the progression of PAAD [9, 10]. Studies have shown that M2 macrophages, through the secretion of cytokines such as IL-10 and TGF-β, suppress anti-tumor immune responses, promote tumor cell migration and invasion, and support tumor growth by regulating cancer cell metabolism [11–13]. As a result, the functional and metabolic state of TAMs within the PAAD immune microenvironment has become a key focus in current cancer immunology research.

In recent years, metabolic reprogramming has been recognized as a hallmark of cancer cells, and the Warburg effect—whereby tumor cells preferentially rely on glycolysis for energy production even under aerobic conditions—plays a pivotal role in tumor cell survival and proliferation [14, 15]. The Ras association domain family member 1 (RASSF1) gene generates multiple isoforms through alternative promoter usage and splicing [16, 17], among which Ras association domain family member 1 isoform A (RASSF1A) is generally regarded as a classical tumor suppressor and is frequently silenced by promoter hypermethylation in PAAD and other malignancies [18]; in addition, dysregulated expression of different RASSF1 isoforms has been observed in pancreatic ductal adenocarcinoma (PDAC) [19]. In contrast, accumulating evidence indicates that Ras association domain family member 1 C (RASSF1C) exerts oncogenic functions in diverse tumor contexts, promoting malignant phenotypes such as tumor cell proliferation, migration and invasion, as well as tumor sphere formation [20–23]. Within the hypoxic TME, hypoxia-inducible factor-1 alpha (HIF-1α) serves as a central transcriptional regulator driving glycolytic gene expression and metabolic adaptation [24]; previous studies have reported that RASSF1A can form a positive feedback loop with HIF-1α to facilitate a glycolytic switch [25]. However, whether and how the oncogenic isoform RASSF1C participates in HIF-1α-mediated metabolic reprogramming, lactate production, and subsequent modulation of the immune microenvironment in PAAD remains unclear. Therefore, this study focused on the mechanistic role of the RASSF1C-HIF-1α axis in PAAD, aiming to clarify its regulatory impact on the Warburg effect, lactate generation, and metabolic remodeling of TAMs.

Although hypoxia-induced lactate accumulation and its role in macrophage polarization have been reported [26–29], existing studies have largely focused on lactate-driven transcriptional remodeling or metabolic shifts in macrophages, whereas systematic evidence addressing whether tumor-derived metabolic signals regulate key immune effector molecules through post-translational modification pathways remains limited. In particular, within the PAAD TME, it has not been clearly elucidated whether lactate influences the stability of critical innate immune regulators via specific protein modification mechanisms. As a major functional isoform of RASSF1, RASSF1C exhibits biological effects distinct from, and in some contexts opposite to, those of the canonical tumor-suppressive isoform RASSF1A. RASSF1C has been implicated in tumorigenesis and progression through its involvement in oncogenic phenotypes, signaling pathway regulation, and transcriptomic or epigenetic modulation across multiple cancer types [20, 30–32]. However, whether RASSF1C participates in hypoxia-induced metabolic alterations and subsequently mediates the crosstalk between tumor metabolic signaling and immune regulation has not been systematically investigated. In addition, ubiquitin-fold modifier 1 ligating enzyme 1 (UFL1)-mediated UFMylation has traditionally been studied in the context of endoplasmic reticulum homeostasis and ribosome quality control [33, 34], and its potential regulatory roles in the TME remain largely unexplored. Notably, interferon regulatory factor 7 (IRF7), a central transcription factor governing innate antiviral responses and immune activation [35–37], has not been experimentally validated to determine whether it is subject to hypoxia- or metabolic stress-induced post-translational modifications, nor whether it is directly regulated by the UFMylation pathway.

Based on the above background and scientific questions, this study focused on the mechanistic role of the RASSF1C-HIF-1α axis in PAAD, aiming to determine whether and how it regulates the Warburg effect and lactate production, and subsequently drives immunometabolic remodeling of TAMs to promote PAAD progression. To address these questions, we integrated single-cell RNA sequencing (scRNA-seq), spatial transcriptomics (ST), and multiple in vitro and in vivo models to dissect lactate-mediated metabolic-immune crosstalk systematically. Notably, we found that hypoxia-induced activation of RASSF1C suppressed UFL1-mediated UFMylation, thereby compromising IRF7 stability and directly linking tumor metabolic reprogramming to innate immune regulation. This finding not only reveals a previously unrecognized molecular mechanism by which lactate drives immune suppression but also provides a potential strategy to target TAM metabolism and enhance immunotherapeutic responses in PAAD.

## Results

### scRNA-seq combined with ST reveals the role of TAMs in the immune microenvironment of PAAD in mice

**Fig. 1:**
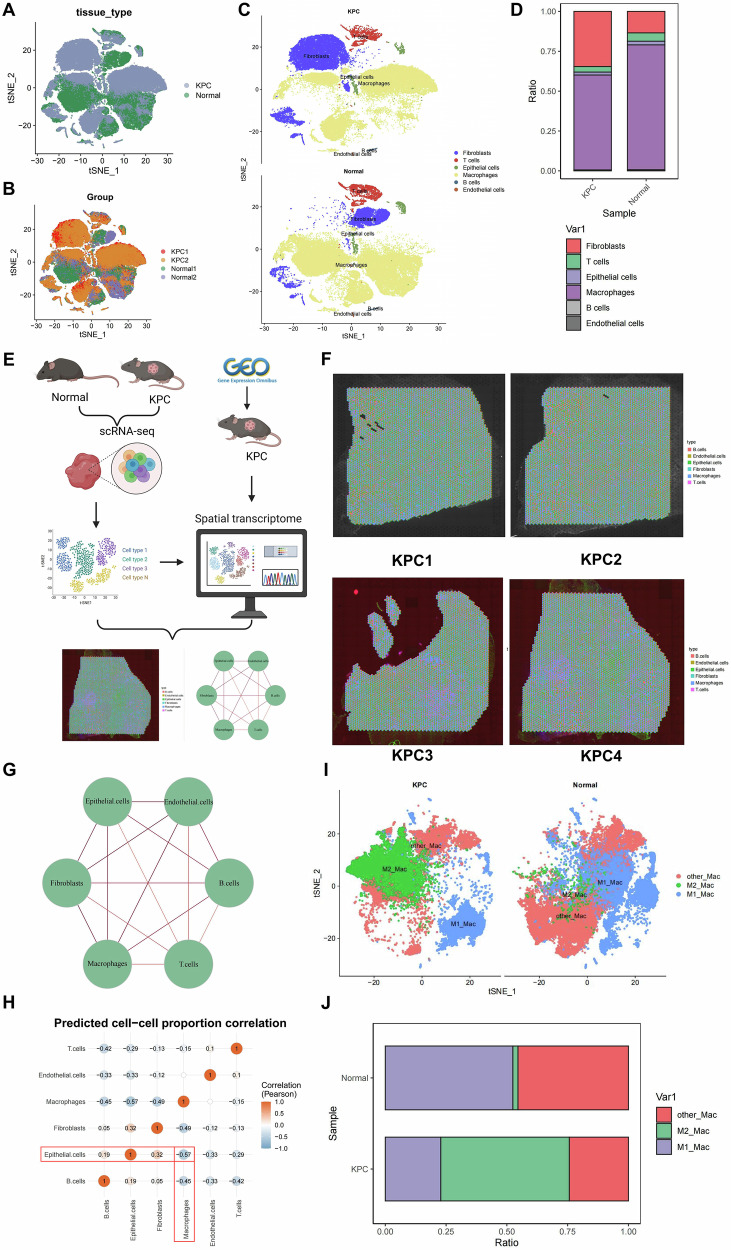
Analysis results of scRNA-seq combined with ST. **A**, **B** t-SNE plots showing cell distribution differences between the KPC model (PAAD) and normal pancreatic tissue. **A** Grouped by tissue type. **B** Grouped by experimental groups. **C** t-SNE plot annotated with six major cell types: endothelial cells, epithelial cells (cancer cells), macrophages (TAMs), fibroblasts, B cells, and T cells. **D** Proportional distribution of different cell types in KPC and normal tissues. **E** Flowchart of combined analysis of scRNA-seq and ST; **F** ST results showing the spatial distribution of cell types in four KPC samples. **G** Network graph of cell interactions, with red lines indicating interaction strength. **H** Heatmap of cell proportion correlation, displaying correlations between cell types. t-SNE plot (**I**) and a bar graph (**J**) show the macrophage subtypes’ distribution.

To explore heterogeneity within the PAAD microenvironment and capture the diversity of cellular states, we established an orthotopic Kras-driven pancreatic cancer (KPC) model using the murine PAAD cell line PANC02, with normal mouse pancreatic tissue serving as the control. Tumor tissues from two tumor-bearing mice were collected and subjected to scRNA-seq analysis (Fig. 1A, B). Quality control, dimensionality reduction, and clustering analyses of the scRNA-seq data were performed using the Seumouse package. Cell annotation was conducted using known cell lineage-specific marker genes identified through a literature review and the CellMarker online database (Fig. S3A, B). This analysis identified six cell types: endothelial cells, epithelial cells (cancer cells), macrophages (TAMs), fibroblasts, B cells, and T cells (Fig. 1C). To facilitate readers’ understanding of the acquisition, quality control, and preprocessing of the scRNA-seq and ST data in this study, we summarized the overall workflow for dual-modality data processing and integrative analysis in an additional schematic diagram (Fig. S4).

Kyoto Encyclopedia of Genes and Genomes (KEGG) pathway analysis of differentially expressed genes (DEGs) in each cell subpopulation revealed specific functional insights. DEGs in endothelial cells were significantly enriched in pathways related to inflammatory responses and signal transduction, such as the IL-17 and TGF-β signaling pathways, as well as functions associated with blood flow shear stress. These findings suggest that endothelial cells may contribute to inflammation and vascular remodeling in the TME. Enrichment analysis of epithelial cells (cancer cells) highlighted metabolic pathways, including cholesterol metabolism, fatty acid absorption, and carbohydrate metabolism, indicating that cancer cells may rely on metabolic reprogramming to support rapid growth. TAMs were enriched in cytokine-cytokine receptor interaction and phagosome pathways, suggesting a role in regulating the immunosuppressive TME. Fibroblast KEGG analysis identified enrichment in ECM-receptor interaction and leukocyte transendothelial migration pathways associated with matrix remodeling and enhanced tumor invasiveness. B cells were enriched in pathways related to immune function, including B cell receptor signaling and antigen presentation, suggesting a potential role in tumor immune responses. Differentially expressed genes in T cells were enriched in pathways related to Th1 and Th2 cell differentiation as well as Th17 cell differentiation, suggesting that the functional diversification of T cell subsets within the TME was subject to regulatory control (Fig. S5A, B). Compared to normal samples, macrophages were significantly reduced in KPC tissues, while fibroblasts were markedly increased. Among all cell types, fibroblasts and TAMs accounted for the largest proportions in tumor tissues (Fig. 1D).

In recent years, integrating ST with scRNA-seq has proven invaluable for characterizing the spatial composition of different cell types within tissues. This combination addresses the loss of spatial information that occurs with scRNA-seq alone and has broad applications in biology [38]. The SPOTlight package utilizes this integration to infer the spatial distribution of cell types and states within tissues and to analyze their interactions [39].

To clarify the strategy for integrating the scRNA-seq reference atlas with ST data, we summarized the computational workflows for anchor-based mapping (Seurat anchors), label transfer, and SPOTlight deconvolution analysis in an additional schematic diagram (Fig. S6). To further characterize the distribution of different cell types in PAAD tissues, we validated our approach using ST data from four mouse models of PAAD. By calculating the overlap between genes localized to specific regions in ST data and the cell-type-specific genes identified in our scRNA-seq data, we inferred the enrichment of specific cell types in given tissue regions and annotated cells in the ST data (Fig. 1E, F). For instance, the distribution of macrophages is shown in Fig. S7A. By integrating our scRNA-seq data and using the R package “SPOTlight,” we obtained spatial information on cellular interactions, visualized as circular plots of interaction strengths (Fig. 1G) and heatmaps of cell correlations (Fig. 1H). The results showed that within the KPC TME, epithelial cells (cancer cells) and macrophages (TAMs) exhibited greater spatial co-distribution, while the spatial proportions of these two cell populations were inversely correlated. Previous studies have demonstrated the high plasticity of macrophages in the TME, with different phenotypes playing critical roles in tumor development and progression. M1 macrophages predominantly produce pro-inflammatory factors, participate in antigen presentation, and exert antitumor effects, whereas M2 macrophages support tumor progression by secreting anti-inflammatory factors and promoting immune suppression [40]. TAMs often exhibit an M2 phenotype, accelerating tumor metastasis and mediating immune evasion [41]. Therefore, targeting TAMs presents a promising novel anticancer strategy. Integrating the scRNA-seq and ST analyses, we summarized the spatial enrichment patterns of major cell types, spatially constrained cell-cell interactions, and their associations with mechanism-related metabolic activities, including hypoxia, glycolysis, and lactate-related processes (Fig. S8).

Further macrophage subset analysis based on the scRNA-seq data revealed that, in KPC samples, the proportion of M2-polarized macrophages was increased compared with that in normal samples, whereas the proportion of M1-polarized macrophages was reduced (Fig. S7B; Fig. 1I, J).

Collectively, these results indicated that scRNA-seq analysis suggested that cancer cells may support rapid growth through metabolic reprogramming. In combination with ST, the data further supported that TAMs constitute the predominant immune cell population in the TME of this model and are spatially proximal to tumor cells, thereby providing a foundation for subsequent mechanistic investigations.

### Hypoxia-Induced RASSF1C Promotes Glycolysis and Progression in PAAD

**Fig. 2:**
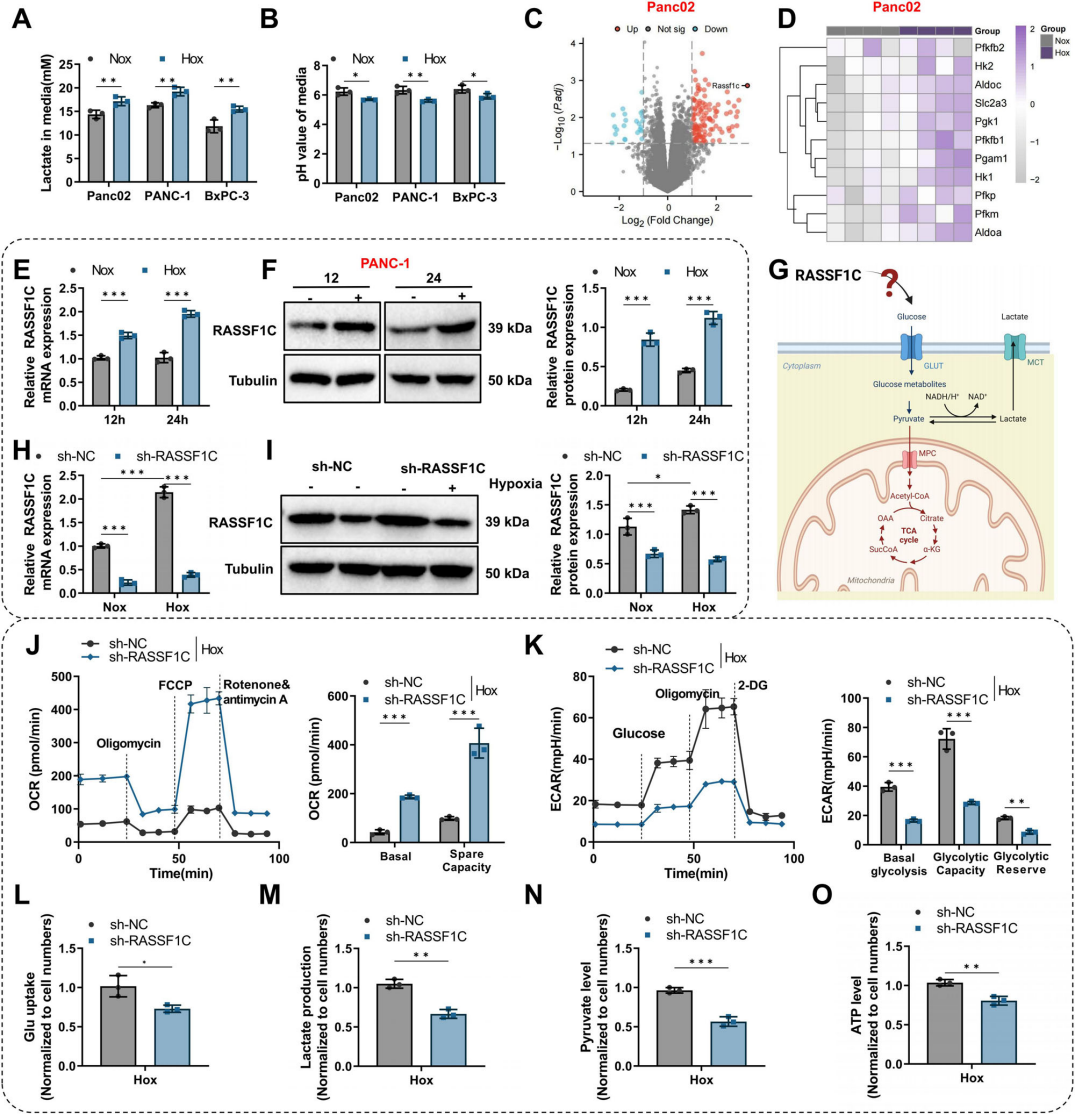
Analysis of RASSF1C’s role in glycolytic metabolism of PAAD under Hox. **A** Measurement of lactate levels in culture supernatants of Panc02, PANC-1, and BxPC-3 cells after normoxic (Nox) or hypoxic (Hox) treatment; **B** pH measurement of culture supernatants under the same conditions; **C** Volcano plot of transcriptomic DEGs in Panc02 cells under Nox and Hox conditions; **D** Heatmap of glycolysis-related gene expression among the DEGs; **E** Relative RASSF1C mRNA expression in cells after 12 h and 24 h of Nox or Hox treatment (qPCR); **F** Detection of RASSF1C protein levels in PANC-1 cells after 12 h and 24 h of hypoxic treatment by Western blot (Tubulin as a loading control), with densitometric quantification; **G** Schematic working model illustrating the regulatory relationship between RASSF1C and glycolysis; **H** Relative RASSF1C mRNA expression in sh-NC and sh-RASSF1C cells under Nox or Hox conditions (qPCR); **I** RASSF1C protein levels in sh-NC and sh-RASSF1C cells under Nox or Hox conditions assessed by Western blot (Tubulin as a loading control), with densitometric quantification; **J** Seahorse XF mitochondrial stress test showing dynamic OCR curves and calculated parameters, with sequential addition of Oligo, FCCP, and rotenone/antimycin A at the indicated time points; **K** Seahorse XF glycolysis stress test showing dynamic ECAR curves and calculated parameters, with sequential addition of glucose, Oligo, and 2-deoxy-D-glucose (2-DG) at the indicated time points; Under hypoxic conditions, assessment of glucose uptake (**L**), lactate production (**M**), pyruvate levels (**N**), and ATP levels (**O**). All cell-based experiments were performed with three independent biological replicates, and data are presented as mean ± SD with individual data points overlaid. ns indicates no statistically significant difference; *p < 0.05, **p < 0.01, ***p < 0.001.

scRNA-seq analysis suggests that PAAD cells may support rapid growth through metabolic reprogramming. Limited and fluctuating oxygen availability in tumors drives cells to adapt metabolically, including shifting towards aerobic glycolysis, known as the “Warburg effect.” This shift increases glycolytic rates, decreases pyruvate oxidation, and enhances lactate production [42, 43]. When PAAD cells were subjected to Hox, we observed an increase in lactate production and a decrease in pH compared to Nox (Fig. 2A, B). To further explore the mechanisms by which PAAD cells regulate their metabolism, we conducted transcriptomic analysis of Panc02 cells under hypoxic conditions. This analysis identified 141 DEGs (Fig. 2C), with significant enrichment in glycolysis/gluconeogenesis pathways (Figure S9A, B). Glycolysis-related genes were notably upregulated in Hox (Fig. 2D). Additionally, we observed a significant upregulation of RASSF1C in the Hox compared to the Nox, with the most substantial fold change (Fig. 2C; Fig. S10A). Additionally, we exposed PAAD cells to Hox for various durations and tracked RASSF1C levels (Fig. S10B). The results show that in PANC-1 cells, acute hypoxic exposure (15 min to 6 h) strongly induced RASSF1C protein expression but had no effect on mRNA expression (Fig. S10C, D). However, after 12 and 24 h of hypoxia, both RASSF1C mRNA and protein expression were significantly increased in PANC-1 and Panc02 cells (Fig. 2E, F). Immunofluorescence results also demonstrated upregulation of RASSF1C in PANC-1 cells (Fig. S10E). Previous studies have indicated that RASSF1C acts as an oncogene in various cancers and is associated with glycolysis-related genes, but the link between RASSF1C and glycolysis induced by PAAD remains to be further confirmed [22, 25, 44] (Fig. 2G).

To investigate whether RASSF1C is involved in the metabolic adaptation of PAAD, we manipulated the expression of RASSF1C in PAAD cells (Fig. S11A, B) and subjected these cells (sh-NC and sh-RASSF1C) to Hox (Fig. 2H, I). Compared to the sh-NC group, RASSF1C expression was significantly reduced. We further examined the impact of RASSF1C knockdown on the glycolytic phenotype under hypoxia. Results showed that RASSF1C knockdown significantly decreased extracellular acidification rate (ECAR) and increased oxygen consumption rate (OCR) (Fig. 2J, K), confirming suppression of glycolysis and enhancement of mitochondrial respiration. Additionally, glucose uptake, lactate production, pyruvate levels, and adenosine triphosphate (ATP) production were notably reduced (Fig. 2L–O), supporting the role of RASSF1C in promoting glycolytic metabolism to adapt to Hox, thereby facilitating rapid cancer growth. Similar effects on glycolytic activity were confirmed in Panc02 cells (Fig. S11C–J).

Previous studies have shown that dysregulation of aerobic glycolysis contributes to malignant progression [45]. Thus, we explored whether RASSF1C-induced aerobic glycolysis affects the growth and metastasis of PAAD. Cell Counting Kit-8 (CCK-8) (Fig. S12A) and Transwell assays (Fig. S12C) demonstrated that overexpression of RASSF1C enhanced the viability, migration, and invasion of PAAD cells; however, these effects were significantly mitigated by treatment with the LDHA inhibitor Oxamate (OXM). Similar experiments in Panc02 cells yielded consistent results (Fig. S12B; Fig. S12D). Subsequently, to determine the role of glycolysis in vivo in RASSF1C-mediated tumor growth regulation, we established breast cancer xenograft models in BALB/c nude mice and C57BL/6 J mice. As expected, overexpression of RASSF1C promoted tumor growth in grafts, and OXM strongly attenuated this effect (Fig. S12E–G), indicating that RASSF1C-induced glycolysis is crucial for PAAD cell growth.

These findings suggest that RASSF1C promotes metabolic adaptation and malignant progression in PAAD by inducing aerobic glycolysis, highlighting its potential as a therapeutic target for PAAD treatment.

### RASSF1C-induced lactate production facilitates TAM activation in PAAD

Solid tumors coexist with various stromal cells within TMEs. Recent studies have identified lactate as a metabolic byproduct and a key signaling molecule regulating the TME. Lactate notably plays a critical role in modulating macrophage function and polarization. To explore the key regulatory mechanisms mediating interactions between tumor cells and TAMs, research has linked lactate to cellular reprogramming in the TME [46, 47]. However, the signaling communications between tumor cells and macrophages remain unclear.

Our analysis of lactate levels in the conditioned medium (CM) of different cancer cell lines compared to macrophages (Raw264.7 and THP-1) revealed significantly higher lactate concentrations and reduced pH values in the CM of PAAD cell lines (PANC-1, Panc02, and BxPC-3) (Fig. S13A, B). This suggests that PAAD cells exhibit greater glycolytic activity than macrophages. Moreover, time-point analyses demonstrated that lactate production in PANC-1 and Panc02 cells increased (6, 12, and 24 h), showing significant time-dependent changes (Fig. S13C). Treatment with the lactate dehydrogenase inhibitor OXM significantly reduced lactate levels in the CM of PANC-1 and Panc02 and ameliorated medium acidification (Fig. S13D).

**Fig. 3:**
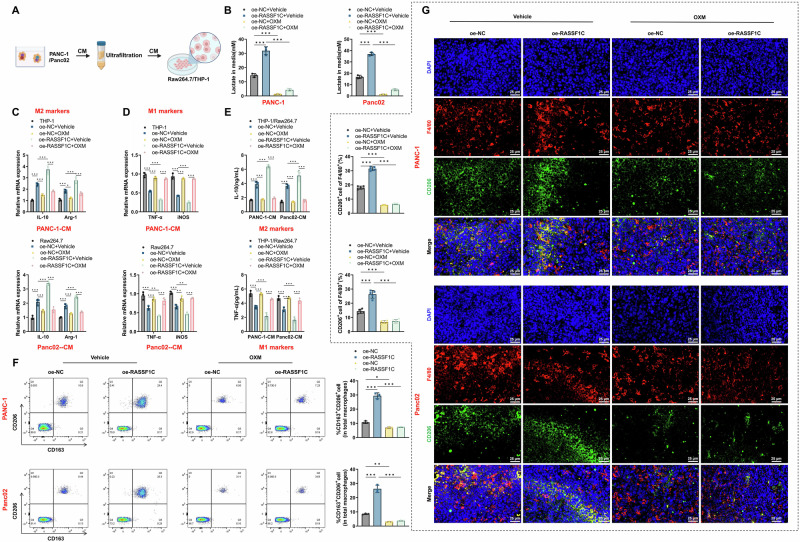
RASSF1C promotes TAM activation through lactate production. **A** Schematic illustration of the experimental workflow for preparation of CM from PANC-1 and Panc02 cells, followed by ultrafiltration and concentration for treatment of RAW264.7 or THP-1 cells; **B** Measurement of lactate concentrations in macrophage culture supernatants after treatment with CM derived from different sources or conditions; **C** Relative mRNA expression of M2-associated markers (IL-10 and Arg-1) in RAW264.7 and THP-1 cells after treatment with PANC-1-CM or Panc02-CM, as determined by RT-qPCR; **D** Relative mRNA expression of M1-associated markers (TNF-α and iNOS) under the same conditions, as determined by RT-qPCR; **E** Quantification of cytokine levels (IL-10 and TNF-α) in macrophage culture supernatants by ELISA; **F** FCM analysis of the proportions of TAM-associated surface markers (CD206 and CD163) in single-cell suspensions from mouse tumor tissues; **G** Immunofluorescence co-staining of tumor tissues showing the distribution and colocalization of F4/80 and CD206 signals, with quantitative analysis of the proportion of CD206⁺ cells (scale bar = 25 μm). Cell-based experiments were performed with three independent biological replicates, and animal experiments included *n* = 5 mice per group. Data are presented as mean ± SD with individual data points overlaid. ns indicates no statistically significant difference; *p < 0.05, **p < 0.01, ***p < 0.001.

We aimed to validate whether lactate derived from cancer cells could regulate TAM polarization. As shown in Fig. 3A, lactate-enriched fractions from the CM of hypoxia-induced PAAD cells were added to macrophage cultures. We observed that CM from RASSF1C-overexpressing PAAD cells increased lactate concentrations, while OXM treatment effectively blocked this increase (Fig. 3B). RT-qPCR analysis indicated that PAAD CM could promote the expression of M2-like TAM markers, including Arg1 and IL-10, and reduce the expression of M1 TAM markers, including TNF-α and iNOS. Furthermore, CM from RASSF1C-overexpressing PANC-1 cells enhanced the expression of M2-like TAM markers and reduced M1 markers, effects that were reversed by OXM addition (Fig. 3C, D). ELISA further confirmed these findings (Fig. 3E), suggesting that acidic signaling in PAAD CM promotes TAM activation via RASSF1C.

Additionally, we employed xenograft models of breast cancer in BALB/c nude and C57BL/6 J mice to investigate the in vivo effects of RASSF1C on macrophage polarization. Results demonstrated that RASSF1C overexpression promoted the expression of M2-like TAM markers CD206/CD163, and OXM strongly mitigated these effects (Fig. 3F, G).

These findings indicate that RASSF1C-induced aerobic glycolysis promotes TAM activation via lactate production.

### HIF-1α promotes RASSF1C expression at the transcriptional level under hypoxia

**Fig. 4:**
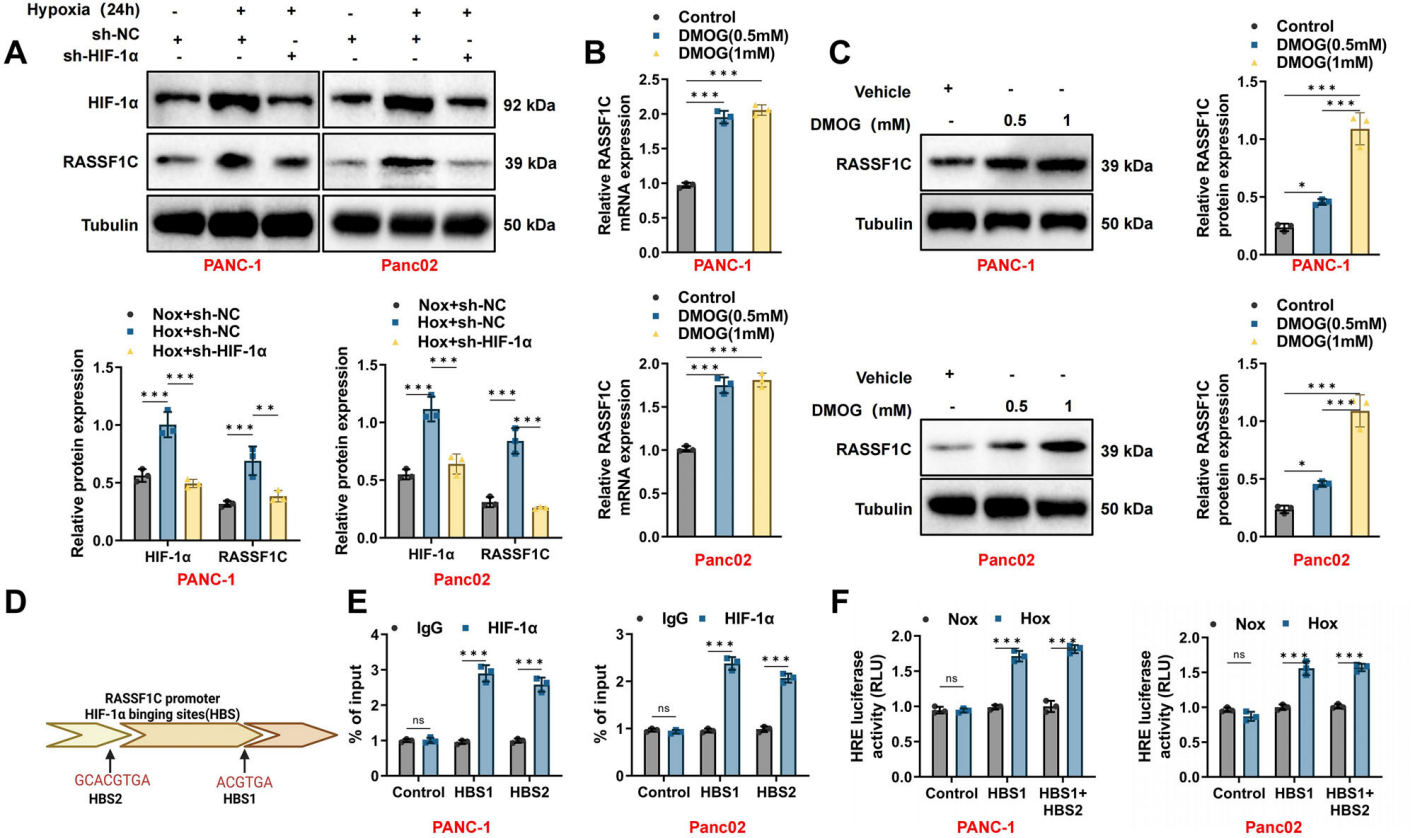
HIF-1α regulates RASSF1C expression by binding to its promoter. **A** Changes in HIF-1α and RASSF1C protein levels in PANC-1 and Panc02 cells after knocking down HIF-1α under Nox and Hox; **B** Changes in RASSF1C mRNA levels in PANC-1 and Panc02 cells treated with PHD inhibitors DMOG (0.5 mM and 1 mM); **C** Effects of DMOG (0.5 mM and 1 mM) on RASSF1C protein expression in PANC-1 cells; **D** Diagram showing HIF-1α HBS in the RASSF1C promoter region; **E** ChIP assay showing enrichment of HIF-1α at HBS1 and HBS2 regions of the RASSF1C promoter under Hox; **F** Luciferase reporter assay demonstrating the activation effect of the HBS1 and HBS1 + HBS2 regions on RASSF1C expression under Hox. All cell-based experiments were performed with three independent biological replicates. Data are presented as mean ± SD with individual data points overlaid. ns indicates no statistically significant difference; *p < 0.05, **p < 0.01, ***p < 0.001.

A central pathway in hypoxia signaling involves the activation of the transcription factor HIF-1α [48]. To identify the upstream signaling events that lead to hypoxia-mediated increases in RASSF1C protein, we employed sh-RNA (sh-HIF-1α-1 & sh-HIF-1α-2) to inhibit HIF-1α signaling (Fig. S14A, B). Results demonstrated that hypoxia induced expression of both HIF-1α and RASSF1C, whereas HIF-1α interference reduced their expression (Fig. 4A). Treatment of human PANC-1 cells with the PHD inhibitor DMOG, which stabilizes and accumulates HIF-1α protein, similarly stabilized RASSF1C expression (Fig. 4B, C). These observations suggest that HIF-1α regulates RASSF1C expression.

To further investigate whether HIF-1α directly binds to the RASSF1C promoter, we utilized the JASPAR database to identify binding sites on the RASSF1C promoter (Fig. S14C, D). Analysis of the human and mouse RASSF1C gene promoter sequences revealed the highest-scoring candidate HBS, selecting the top two for further study (Fig. 4D). Chromatin immunoprecipitation (ChIP) assays conducted on PANC-1 and Panc02 cells exposed to hypoxia for 24 h showed strong enrichment of HIF-1α binding at these sites (Fig. 4E).

Moreover, to test whether the HBS in the RASSF1C promoter function as hypoxia response elements (HREs), we inserted fragments containing one or two binding sites (HBS1 and HBS1 + HBS2) upstream of a luciferase coding sequence in the pGL3 plasmid. Transfection of PANC-1 and Panc02 cells with these RASSF1C-HBS1 or HBS1 + HBS2 reporter genes and a control reporter (pGL3-Renilla) followed by exposure to Hox for 24 h significantly increased luciferase activity in hypoxic PANC-1 and Panc02 cells (Fig. 4F).

These data indicate that the increase in RASSF1C expression under sustained hypoxia is mediated by HIF-1α binding to sites within the RASSF1 promoter.

### Regulation of HIF-1α stability and reactivation by RASSF1C

**Fig. 5:**
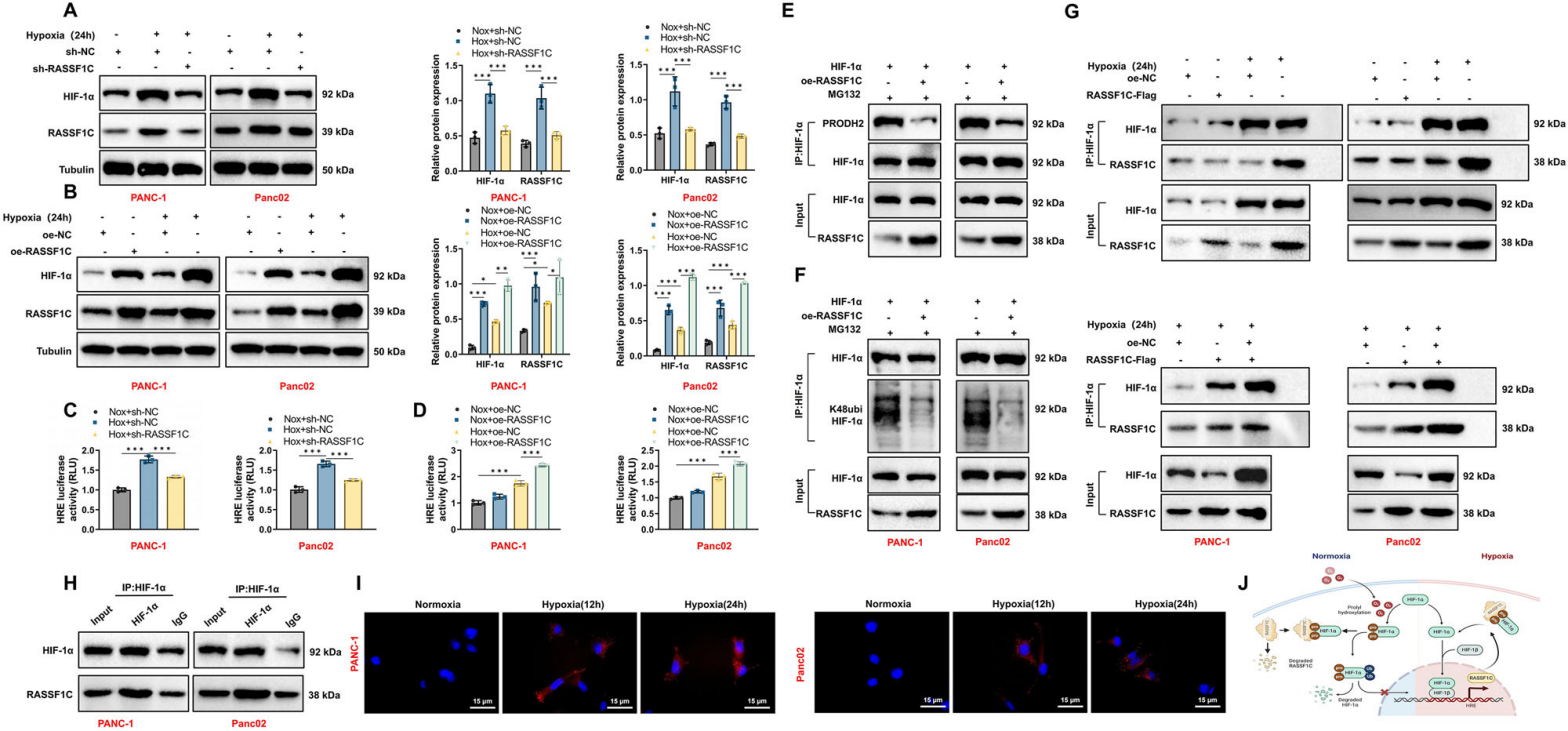
RASSF1C modulates HIF-1α stability and transcriptional activity via direct interaction. **A** Changes in HIF-1α and RASSF1C protein levels in PANC-1 and Panc02 cells under hypoxia after RASSF1C knockdown. **B** Changes in HIF-1α and RASSF1C protein levels in PANC-1 and Panc02 cells under hypoxia after RASSF1C overexpression. **C** Impact of RASSF1C knockdown on the transcriptional activity of the HIF-1α-dependent reporter plasmid HRE-PGL3. **D** Impact of RASSF1C overexpression on the transcriptional activity of the HIF-1α-dependent reporter plasmid HRE-PGL3. **E** Effect of MG132 treatment on prolyl hydroxylation levels of HIF-1α after RASSF1C overexpression. **F** Effect of MG132 treatment on ubiquitination levels of HIF-1α after RASSF1C overexpression. **G** Co-IP experiment assessing the binding of RASSF1C to HIF-1α under hypoxia. **H** Co-IP experiment confirming the binding of endogenous RASSF1C to HIF-1α under hypoxia. **I** PLA detecting interactions between RASSF1C and HIF-1α at different time points under hypoxia (scale bar = 15 µm). **J** Schematic representation of RASSF1C regulating HIF-1α stability. All cell-based experiments were performed with three independent biological replicates. Data are presented as mean ± SD with individual data points overlaid; *p < 0.05, **p < 0.01, ***p < 0.001.

To explore the regulatory relationship between RASSF1C and HIF-1α, we employed shRNA to suppress RASSF1C expression in PANC-1 and Panc02 cells. This intervention significantly reduced the protein levels of HIF-1α and RASSF1C under Hox (Fig. 5A). Conversely, overexpression of RASSF1C in these cell lines increased HIF-1α protein levels (Fig. 5B) and rescued the effects of RASSF1C knockdown on HIF-1α expression (Fig. S15A). To determine if RASSF1C impacts HIF-1α-mediated transcriptional activity, PANC-1 and Panc02 cells were co-transfected with HIF-1α-dependent reporter plasmid HRE-PGL3. After 24 h of hypoxia, cells with RASSF1C knockdown showed a significant reduction in HIF-1α transcriptional activity, whereas RASSF1C overexpression enhanced it (Fig. 5C, D).

Notably, following RASSF1C knockdown, levels of HIF-1α protein decreased; however, treatment with the proteasome inhibitor MG132 resulted in a dose-dependent increase in HIF-1α levels after RASSF1C knockdown (Fig. S15B). PANC-1 and Panc02 cells overexpressing NC or RASSF1C were transfected with HIF-1α to confirm these observations. Post-transfection, cells were subjected to hypoxia for 5 h in the presence of 25 μM MG132, and cell lysates were immunoprecipitated with an anti-HIF-1α antibody, followed by immunoblotting with antibodies against ubiquitin (K48ubi) and hydroxyproline (PRODH2). Expression of RASSF1C-Flag led to reduced hydroxylation of proline in HIF-1α (Fig. 5E) and decreased ubiquitination (Fig. 5F), suggesting that RASSF1C stabilizes HIF-1α by inhibiting its proline hydroxylation.

To hypothesize that the physical interaction between RASSF1C and HIF-1α underlies its effect on hypoxia signaling, co-immunoprecipitation (Co-IP) experiments were conducted in hypoxic cells expressing oe-RASSF1C and HIF-1α. RASSF1C-Flag was precipitated with an anti-HIF-1α antibody and vice versa (Fig. 5G). Similarly, endogenous RASSF1C was specifically precipitated with an anti-HIF-1α antibody in hypoxic PANC-1 and Panc02 cells (Fig. 5H). Proximity ligation assay (PLA) further confirmed these interactions, predominantly localized in the cytoplasm at various hypoxia time points (Fig. 5I), consistent with the primary cytoplasmic localization of RASSF1C (Fig. S15C). These findings suggest that RASSF1C interacts with HIF-1α, preventing its prolyl hydroxylation, ubiquitination, and degradation, thereby enhancing its transcriptional activity (Fig. 5J).

### Regulation of IRF7 by tumor-derived lactate enhances PAAD cell adhesion, migration, and invasion

**Fig. 6:**
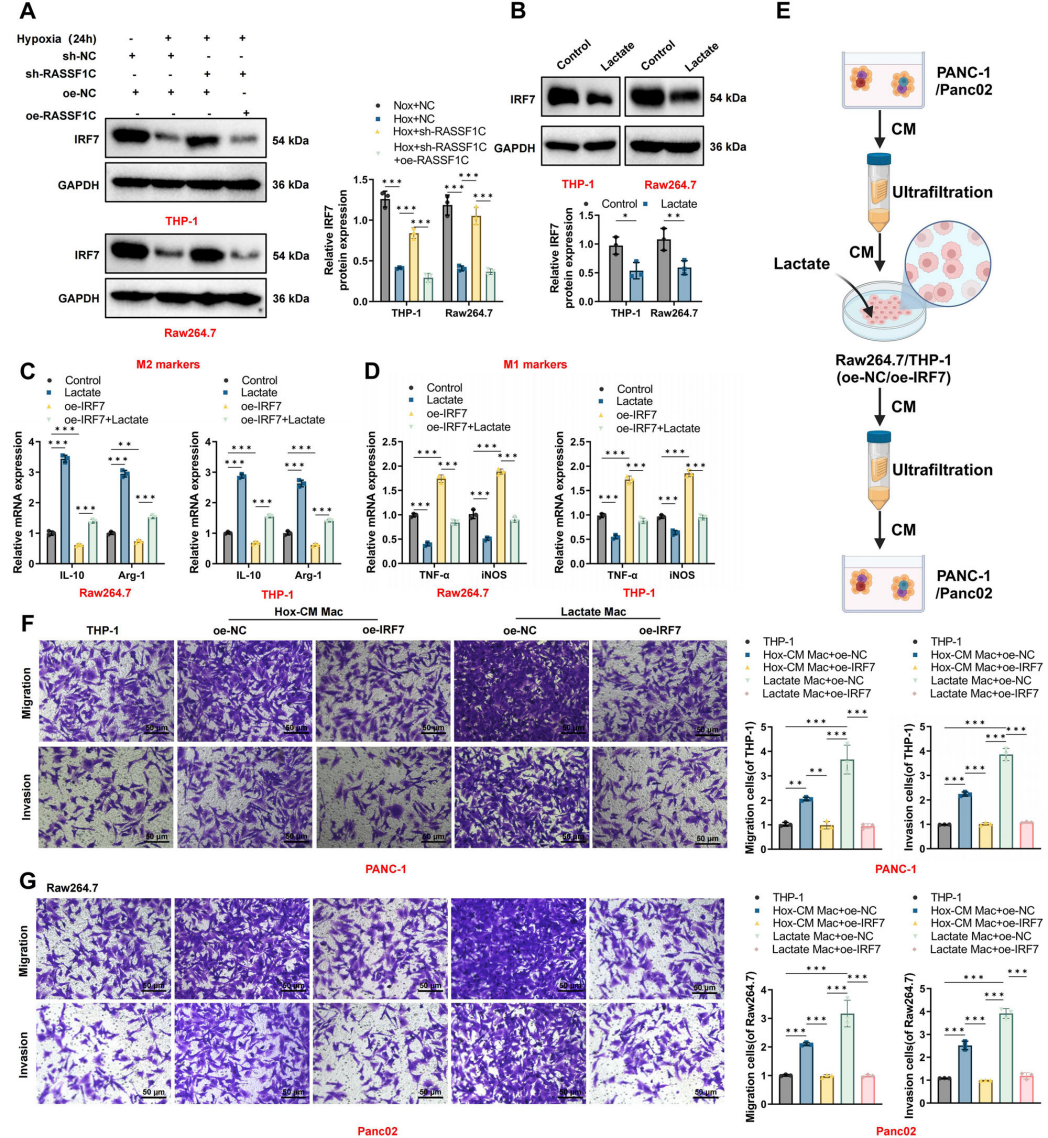
Lactate-activated macrophages influence PAAD cell migration and invasion through IRF7. **A** Changes in IRF7 expression in THP-1 and Raw264.7 macrophages after hypoxia or lactate treatment. **B** Effect of lactate on IRF7 expression in macrophages. **C** Influence of lactate on M2 marker expression in macrophages. **D** Influence of lactate on M1 marker expression in macrophages. **E** Schematic of various pre-treated macrophages and their CM impacting PAAD cells. **F**, **G** Transwell assays showing the effects of macrophage-CM on PAAD cell migration and invasion (scale bar = 50 µm). All cell-based experiments were performed with three independent biological replicates. Data are presented as mean ± SD with individual data points overlaid; *p < 0.05, **p < 0.01, ***p < 0.001.

Previous research has demonstrated that M2 macrophages promote tumor metastasis by secreting various factors [46]. Our prior studies revealed that IRF7 induces polarization of M1 macrophages, regulates lipid metabolism in PAAD, and thereby inhibits tumor formation [49]. Public databases indicate low expression of IRF7 in PAAD, with its expression level positively correlating with M1 macrophage infiltration (Fig. S16A, B). To explore whether tumor-derived lactate affects macrophage activation via IRF7, we co-cultured macrophages with CM from hypoxia-treated PAAD cells transfected to overexpress or knockdown RASSF1C. Our results showed that hypoxia-induced PAAD cell CM suppressed IRF7 expression in macrophages, whereas RASSF1C overexpression rescued the IRF7 downregulation caused by RASSF1C knockdown (Fig. 6A). Exogenous lactate also suppressed IRF7 expression in macrophages (Fig. 6B). To confirm the role of the lactate-IRF7 axis in TAM activation, we treated macrophages with exogenous lactate, both with and without overexpressed IRF7. RT-qPCR analysis showed that lactate enhanced the expression of M2-like TAM markers, including Arg1 and IL-10, in Raw264.7 and THP-1 cells, and reduced the expression of M1 markers, including TNF-α and iNOS. Overexpression of IRF7, however, suppressed the expression of Arg1 and IL-10 and enhanced TNF-α and iNOS expression, thereby negating lactate-mediated M2 polarization of macrophages (Fig. 6C, D). These results suggest that lactate from cancer cells can stimulate M2 polarization of macrophages by inhibiting IRF7.

We further investigated whether lactate-induced M2 macrophages facilitate the migration and invasion of PAAD cells by modulating IRF7. We examined the effects of CM from macrophages pretreated with various treatments on PAAD cell migration and invasion (Fig. 6E). Transwell assays showed that CM from hypoxia-treated PAAD cells and lactate-activated macrophages significantly promoted PAAD cell migration and invasion, and these effects were abrogated by overexpressing IRF7 (Fig. 6F, G). This suggests that overexpression of IRF7 in macrophages can mitigate the effects of endogenous lactate in cancer cells and exogenously added lactate in PAAD cell CM.

To further confirm that lactate is the key factor in cancer cell CM that activates M2 macrophages and promotes cancer cell metastasis, we pretreated cancer cells with OXM and then used their lactate-depleted CM to culture macrophages for tumor cell transwell experiments (Fig. S8D). Results indicated that the impact of PAAD cell CM-activated oe-NC macrophages on cancer cell migration and invasion was nullified by OXM pretreatment but restored by adding exogenous lactate to the OXM-pretreated cancer cell CM. In contrast, macrophage interference did not respond to these treatments (Fig. S16C, D), indicating that IRF7 is a critical mediator of lactate-driven macrophage activation.

These findings suggest that lactate produced by cancer cells activates macrophages, which, in turn, promote PAAD cell metastasis by suppressing IRF7.

### IRF7 is essential for lactate-driven rewiring of macrophage lipid metabolism

**Fig. 7:**
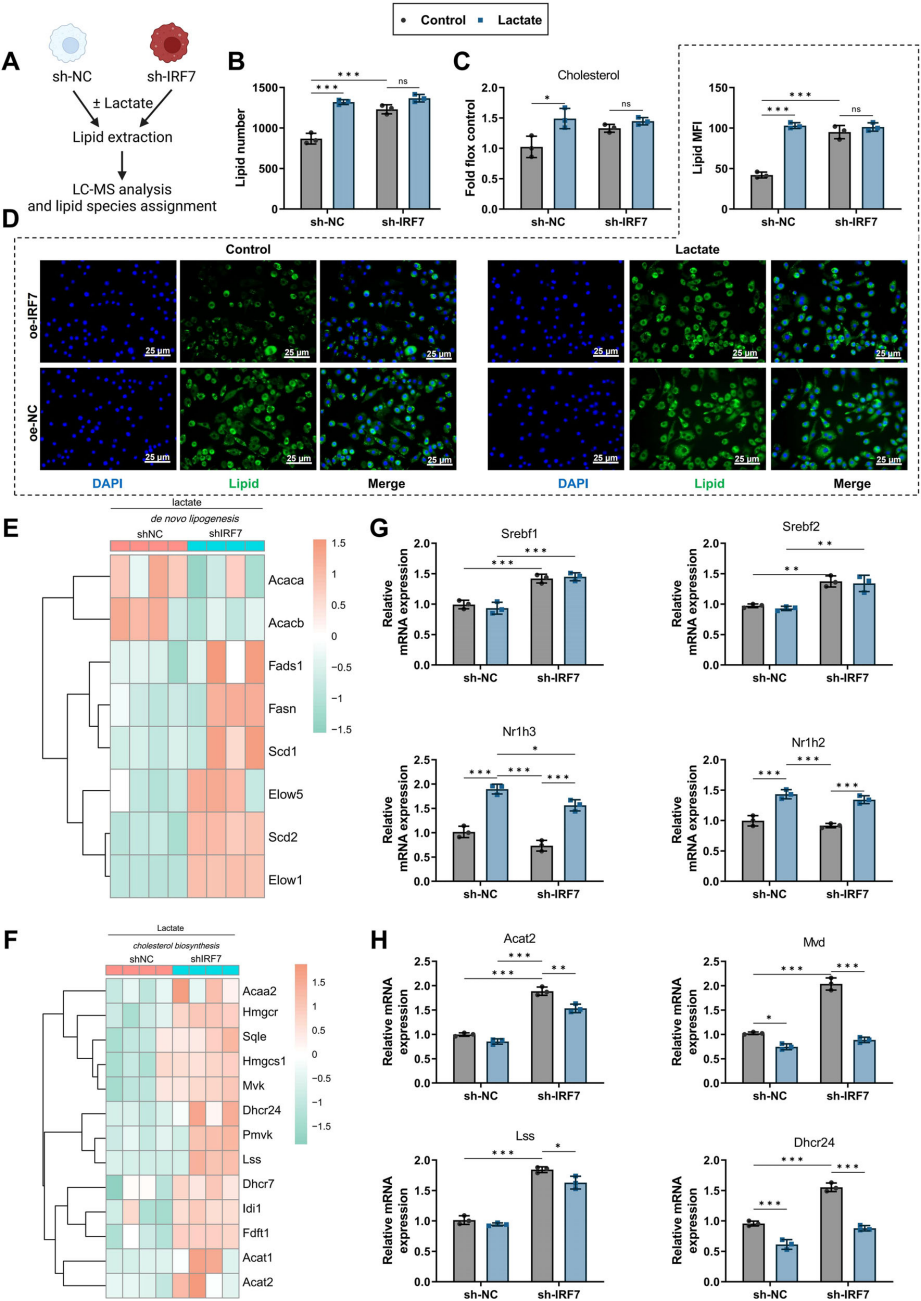
The key role of IRF7 in lipidomic reprogramming of macrophages stimulated by lactate. **A** Schematic of lipid extraction and LC-MS analysis in macrophages after lactate stimulation. **B** Changes in total lipid metabolites in macrophages. **C** Semi-quantitative results of lipid accumulation using BODIPY 493/503 dye in macrophages. **D** Changes in free cholesterol levels in macrophages. **E**, **F** Heatmaps from RNA-seq analysis showing genes involved in de novo lipogenesis and cholesterol biosynthesis. **G** RT-qPCR validation of lipid metabolism transcription factors (Srebf1 and Srebf2) and nuclear receptor genes (Nr1h3 and Nr1h2) under various conditions. **H** RT-qPCR validation of cholesterol biosynthesis-related genes under lactate stimulation and IRF7 knockdown conditions. All cell-based experiments were performed with three independent biological replicates. Data are presented as mean ± SD with individual data points overlaid. ns indicates *P* > 0.05; *p < 0.05, **p < 0.01, ***p < 0.001.

Previous studies have demonstrated that tumor cells drive macrophage polarization toward an M2 phenotype by promoting lipid accumulation [50]. Given the role of IRF7 in lipid synthesis, we investigated the impact of lactate on lipid metabolism in THP-1 macrophages using liquid chromatography-mass spectrometry (LC-MS) [49] (Fig. 7A). Our results revealed that lactate treatment significantly increased the total lipid metabolites in macrophages compared to the control group. However, this increase was less pronounced when IRF7 was knocked down (Fig. 7B). To further validate these findings, we employed BODIPY 493/503 staining for fluorescence and semi-quantitative analysis of lipid accumulation. Lactate-treated Raw264.7 macrophages showed significantly higher lipid accumulation, which was attenuated by IRF7 knockdown (Fig. 7C). Further evaluation using LC-MS indicated a trend towards increased free cholesterol levels after lactate stimulation, although the cholesterol levels in IRF7-knocked-down macrophages remained unchanged post-lactate treatment (Fig. 7D).

Transcriptional analysis via RNA-seq showed that, compared to controls, lactate-stimulated IRF7-knocked-down macrophages had significantly upregulated expression related to de novo lipogenesis and cholesterol biosynthesis pathways (Fig. 7E, F). This was associated with a slight increase in the baseline expression levels of the lipid metabolism-regulating transcription factors Srebf1 and Srebf2. Expression levels of Nr1h3 (encoding LXRα) and Nr1h2 (encoding LXRβ) were similar at baseline and post-lactate treatment (Fig. 7G). RT-qPCR confirmed key gene expression in cholesterol synthesis (Fig. 7H). IRF7-knocked-down macrophages displayed upregulation of genes involved in cholesterol synthesis; however, this upregulation was diminished following lactate stimulation (Fig. 7H).

These data indicate that IRF7 is essential for lactate-induced rewiring of macrophage lipid metabolism, and its absence results in significant metabolic defects. Macrophages with reduced IRF7 levels may upregulate lipogenic genes as a compensatory mechanism to mitigate this deficiency partially, yet they fail to restore metabolic function under lactate stimulation fully.

### UFL1 and IRF7 interact via UFMylation to promote ubiquitination

UFMylation is an emerging ubiquitin-like (UBL) modification system involving a cascade of three enzymes: the E1 ubiquitin-activating enzyme 5 (UBA5), the E2 UFM1-conjugating enzyme 1 (UFC1), and the E3 UFL1 (also known as RCAD, KIAA0776, NLBP, or MAXER). This system covalently attaches UFM1 to target proteins [51]. Using the TIMER v2.0 database for immune infiltration analysis, we found positive correlations between UFL1 and UBA5 and M1 macrophage infiltration levels, whereas UFC1 showed a negative correlation (Fig. S17A–C). Additionally, UFL1 is underexpressed in PAAD (Fig. S17D). Existing research indicates that UFL1 can interact with IRF7 [52]. We explored the influence of PAAD cell-CM under Hox on macrophage UFL1 expression. The results demonstrated that hypoxia significantly suppressed UFL1 expression in macrophages, an effect that was reversible upon RASSF1C overexpression (Fig. S17E). Moreover, treating macrophages with exogenous lactate markedly reduced UFL1 protein levels (Fig. S17F), suggesting that PAAD cells may suppress macrophage UFL1 expression via lactate, thereby modulating IRF7’s role in macrophages.

**Fig. 8:**
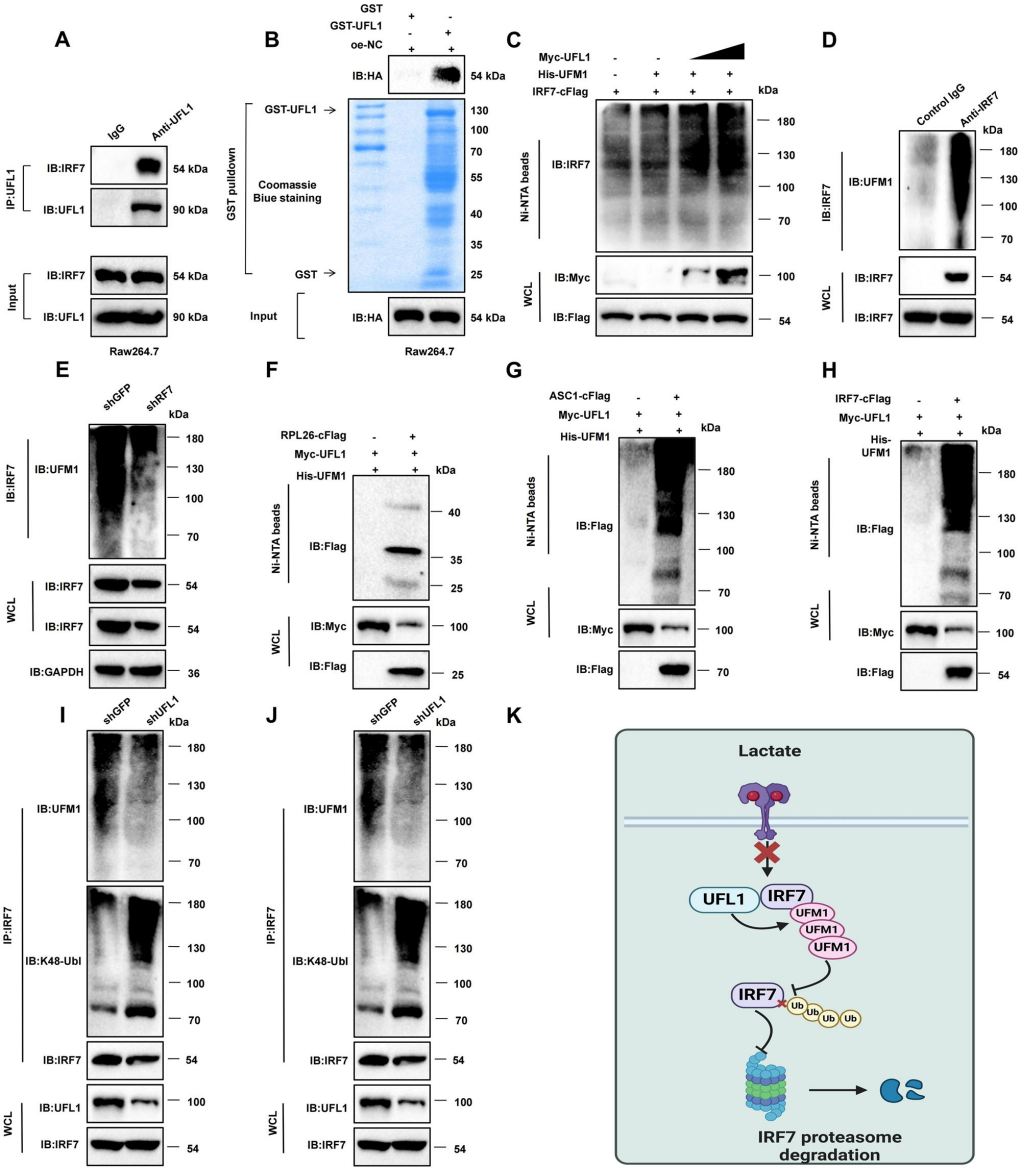
Interaction of UFL1 and IRF7 through UFMylation promotes their ubiquitination. **A** IP demonstrates the interaction between endogenous UFL1 and IRF7. **B** GST pull-down assay confirming the interaction between recombinant UFL1 and IRF7. **C** In vivo UFMylation assay showing UFL1 enhances IRF7 UFMylation. **D** Detection of endogenous UFMylation of IRF7 in Raw264.7 cells. **E** The high molecular weight UFMylation bands disappeared in Raw264.7 cells upon IRF7 knockdown. **F**, **G** Experimental verification of UFMylation in known substrates RPL26 and ASC1. **H** Poly-UFMylation of IRF7 under similar experimental conditions as RPL26 and ASC1. **I** Reduction of IRF7 UFMylation and increase in K48-linked ubiquitination in Raw264.7 cells following UFL1 knockdown. **J** Reduction of IRF7 UFMylation and increase in K48-linked ubiquitination following exogenous lactate treatment. **K** Schematic showing how lactate inhibits IRF7 UFMylation through UFL1, promoting its degradation. Experiments were repeated at least three times.

To further establish a direct link, immunoprecipitation (IP) experiments revealed endogenous interactions between UFL1 and IRF7 in Raw264.7 cells (Fig. 8A). Glutathione S-transferase (GST) pull-down assays confirmed the interaction between recombinant GST-UFL1 and IRF7 (Fig. 8B). UFL1 stabilizes its substrates by promoting UFMylation, thereby counteracting ubiquitination [53]. We investigated whether UFL1 could enhance IRF7’s UFMylation. In vitro UFMylation assays showed that UFL1 significantly increased IRF7’s UFMylation (Fig. 8C), with endogenous IRF7 in Raw264.7 cells also exhibiting UFMylation modifications (Fig. 8D). Knockdown of IRF7 resulted in the disappearance of high molecular weight UFMylated bands, further indicating that IRF7 undergoes poly-UFM1 chain modification (Fig. 8E). We used the known UFMylation substrates RPL26 and ASC1 as controls, which exhibited tri-UFMylation and poly-UFMylation, respectively (Fig. 8F, G). Under similar conditions, IRF7 displayed poly-UFMylation comparable to ASC1 (Fig. 8H). To explore the role of UFMylation in IRF7’s function, we knocked down UFL1 or treated cells with exogenous lactate. Both treatments significantly reduced IRF7’s UFMylation levels while increasing K48-linked ubiquitination (Fig. 8I, J). These data suggest that UFL1 stabilizes IRF7 by catalyzing its UFMylation, effectively countering its K48-linked ubiquitination and degradation. Lactate may reduce IRF7 stability by inhibiting UFL1-mediated UFMylation in macrophages, impacting their function (Fig. 8K).

### Expression patterns and correlation analysis of RASSF1C-HIF-1α-UFL1-IRF7 in human PDAC tissues

**Fig. 9:**
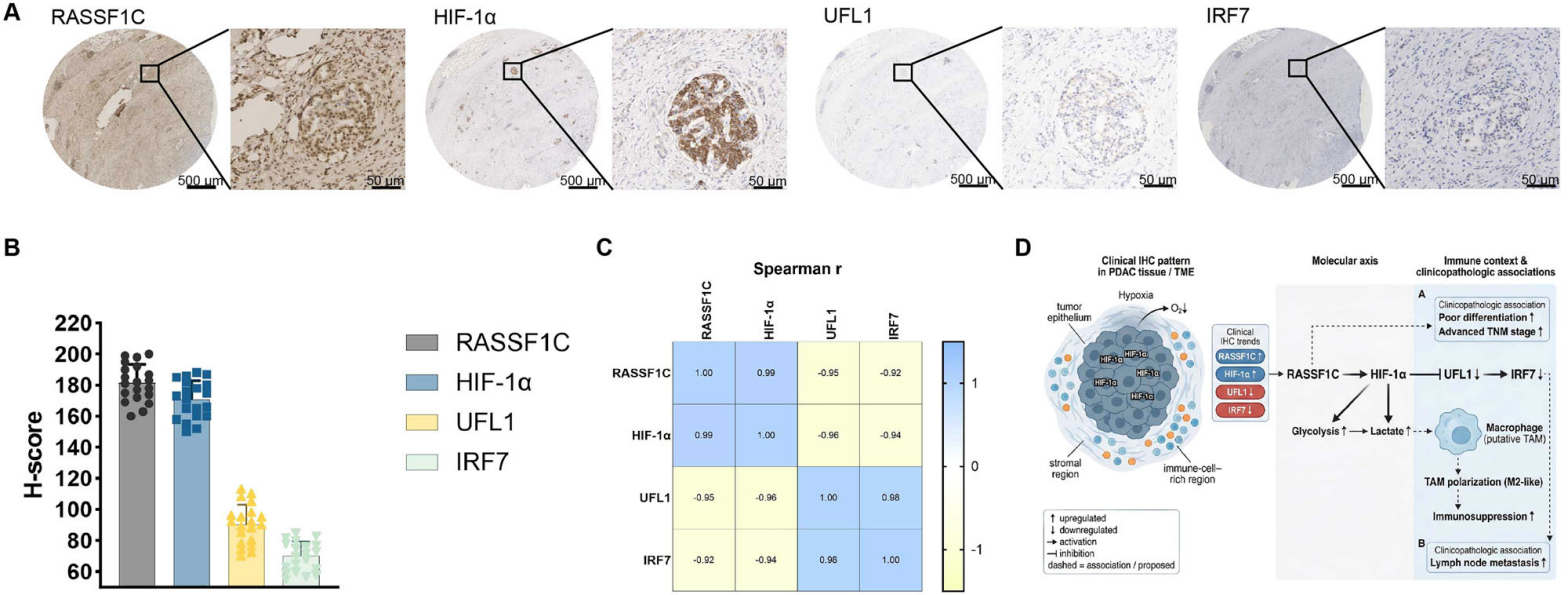
Expression pattern of the RASSF1C-HIF-1α-UFL1-IRF7 axis in human PDAC tissues. **A** Representative IHC staining images of RASSF1C, HIF-1α, UFL1, and IRF7 in tissue microarrays from 20 PDAC cases. Black boxes indicate tumor regions enriched with inflammatory cells and/or TAMs based on morphological features. **B** Semi-quantitative H-score analysis of RASSF1C, HIF-1α, UFL1, and IRF7 expression in PDAC samples (data are presented as mean ± SD, *n* = 20). **C** Heatmap showing Spearman correlation analysis based on the H-scores of the four markers across the 20 PDAC cases; numbers within the heatmap represent Spearman correlation coefficients (r). **D** Schematic illustration of the expression pattern of the RASSF1C-HIF-1α-UFL1-IRF7 axis in PDAC tissues, integrating clinical IHC findings with in vivo and in vitro mechanistic studies to summarize the overall expression trends of this signaling axis within tumor tissues.

To characterize the expression features of the RASSF1C–HIF-1α–UFL1–IRF7 axis in clinical PDAC, we performed immunohistochemical (IHC) staining on a tissue microarray comprising 20 PDAC cases and quantified staining using semi-quantitative H-scores (patient baseline characteristics are summarized in Table 1). RASSF1C and HIF-1α generally showed relatively strong staining in the tumor epithelial compartment, whereas UFL1 and IRF7 exhibited comparatively weaker staining. In tumor regions with more prominent inflammatory cell infiltration, the spatial pattern of “higher RASSF1C/HIF-1α and lower UFL1/IRF7” was more evident (Fig. 9A). H-score quantification was consistent with these observations, indicating overall higher expression levels of RASSF1C and HIF-1α compared with UFL1 and IRF7 (Fig. 9B).

**Table 1:** Baseline clinicopathological characteristics of patients (ungrouped, i = 20).

Characteristic	Overall (n = 20)
Age (years)	62.4 ± 9.1
Sex, *n* (%)	
Female	16 (42.1)
Male	22 (57.9)
Tumor size (cm)	3.2 ± 1.0
Tumor size category, *n* (%)	
<2 cm	11 (28.9)
≥2 cm	27 (71.1)
Histological grade, *n* (%)	
Well/moderately differentiated	20 (52.6)
Poorly differentiated	18 (47.4)
TNM stage, *n* (%)	
Stage I–II	16 (42.1)
Stage III–IV	22 (57.9)
Lymph node metastasis, *n* (%)	
No	14 (36.8)
Yes	24 (63.2)
Tumor location, *n* (%)	
Pancreatic head	24 (63.2)
Pancreatic body/tail	14 (36.8)

Continuous variables are presented as mean ± SD; categorical variables are presented as n (%).

Spearman rank correlation analysis revealed a strong positive correlation between RASSF1C and HIF-1α (*r* = 0.986, *p* = 2.2496 × 10⁻¹⁵, 95% CI 0.9624–0.9946). In contrast, RASSF1C showed significant inverse correlations with UFL1 (i = −0.953, *p* = 8.3881 × 10⁻¹¹, 95% CI − 0.9822 to −0.8805) and IRF7 (*r* = −0.922, *p* = 7.7963 × 10⁻⁹, 95% CI − 0.9699 to −0.8046). HIF-1α similarly exhibited significant negative correlations with UFL1 (*r* = −0.962, *p* = 1.5009 × 10⁻¹¹, 95% CI − 0.9854 to −0.9010) and IRF7 (*r* = −0.936, *p* = 1.3465 × 10⁻⁹, 95% CI − 0.9755 to −0.8384). Notably, UFL1 and IRF7 were strongly positively correlated (*r* = 0.979, *p* = 7.1962 × 10⁻¹⁴, 95% CI 0.9449–0.9920) (Fig. 9C).

Collectively, the staining distribution, H-score quantification, and correlation results (Fig. 9A–C) were integrated to construct the proposed working model (Fig. 9D).

### Mechanistic analysis of UFMylation-mediated regulation of IRF7 protein stability

**Fig. 10:**
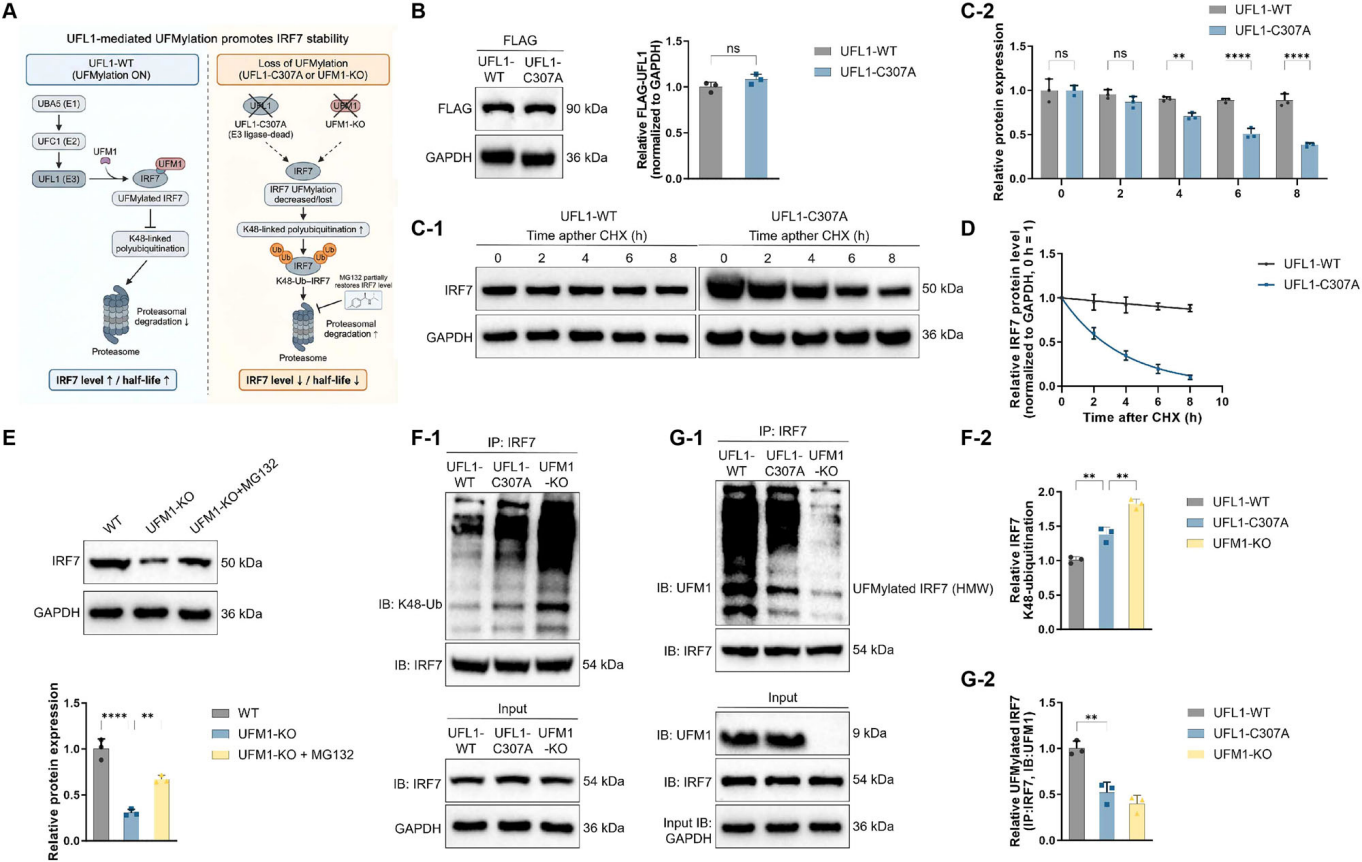
Mechanism by which UFMylation regulates IRF7 protein stability. **A** Schematic illustration of the mechanism by which UFL1-mediated UFMylation maintains IRF7 protein stability. **B** Expression of FLAG-UFL1-WT and FLAG-UFL1-C307A in RAW264.7 macrophages. **C** Western blot analysis and quantification of IRF7 protein levels at the indicated time points after CHX treatment in RAW264.7 cells overexpressing UFL1-WT or UFL1-C307A. **D** IRF7 protein half-life curves derived from the CHX chase experiments. **E** Effects of UFM1-KO alone or in combination with the proteasome inhibitor MG132 on IRF7 protein levels. **F** Detection of IRF7 UFMylation under denaturing IP conditions: UFL1-WT promoted IRF7 UFMylation, whereas UFL1-C307A overexpression or UFM1-KO markedly attenuated this modification. **G** Detection of K48-linked ubiquitination of IRF7 under hypoxic conditions: UFL1-WT reduced K48-linked ubiquitination of IRF7, whereas UFL1-C307A failed to suppress this modification, resulting in enhanced K48-linked ubiquitination of IRF7. Data are presented as mean ± SD and were obtained from at least three independent experiments (*n* = 3). Statistical analysis was performed using one-way ANOVA followed by Tukey’s multiple-comparison test; time-course analyses and IRF7 half-life calculations in **C**, **D** were analyzed using two-way ANOVA with post hoc multiple comparisons. ns indicates no statistically significant difference (*p* ≥ 0.05); *p < 0.05, **p < 0.01, ***p < 0.001, ****p < 0.0001.

To investigate whether UFL1-mediated UFMylation is involved in maintaining IRF7 protein homeostasis, we first generated a catalytically inactive UFL1 mutant, C307A (Fig. 10A). Western blot analysis showed that FLAG-UFL1-C307A was expressed at a level comparable to that of wild-type UFL1 (UFL1-WT) in RAW264.7 macrophages (Fig. 10B), thereby excluding potential confounding effects due to differences in protein expression and providing a reliable basis for subsequent functional comparisons.

We then performed cycloheximide (CHX) chase assays to analyze the kinetics of IRF7 protein degradation. In contrast to the relative stability of IRF7 over an 8 h period in UFL1-WT cells, IRF7 protein levels in UFL1-C307A cells declined markedly over time. Quantitative analysis combined with exponential decay fitting further demonstrated a significant reduction in the half-life of IRF7 under the UFL1-C307A condition (Fig. 10C, D). These results indicate that UFL1 catalytic activity plays a critical role in maintaining IRF7 protein stability.

To further assess the contribution of UFMylation itself to this process, UFM1 was knocked out in macrophages using CRISPR/Cas9. Genomic PCR and Western blot analyses confirmed the complete absence of UFM1 at both the genomic and protein levels in the selected clones (Fig. S18A, B). Similar to the effects observed in UFL1-C307A cells, UFM1 knockout significantly reduced IRF7 protein levels, and this reduction was partially reversed by the proteasome inhibitor MG132 (Fig. 10E), suggesting that loss of UFMylation at least in part promotes proteasome-dependent degradation of IRF7. Under denaturing conditions, IP of IRF7 revealed that UFL1-WT markedly enhanced IRF7 UFMylation, whereas IRF7 UFMylation signals were nearly abolished in cells overexpressing UFL1-C307A or in UFM1-KO cells (Fig. 10F). In addition, under hypoxic conditions, UFL1-WT suppressed K48-linked ubiquitination of IRF7, whereas UFL1-C307A completely lost this inhibitory effect, accompanied by increased levels of K48-linked ubiquitination of IRF7 (Fig. 10G).

Collectively, these findings demonstrate that UFL1-catalyzed UFMylation represents a key regulatory step in controlling IRF7 protein stability. When UFMylation is impaired, IRF7 becomes more susceptible to K48-linked ubiquitination and accelerated proteasomal degradation, thereby providing mechanistic support for the previously proposed “RASSF1C-HIF-1α-UFL1/UFM1-IRF7” regulatory axis.

## Discussion

PAAD is recognized as a highly invasive malignancy with formidable challenges in early diagnosis and treatment, resulting in poor prognoses [54, 55]. Recent studies have highlighted the critical role of the TME in cancer progression and immune evasion, particularly in immunosuppressive tumors like PAAD, where the TME is particularly complex [7, 56, 57]. In this study, we focused on the role of the RASSF1C-HIF-1α axis in PAAD and proposed that it contributes to disease progression by regulating the Warburg effect, promoting lactate production, and driving metabolic remodeling in macrophages. Notably, although previous reports have described divergent functions of RASSF1 across different tumor contexts, these apparent discrepancies largely stem from functional differences among RASSF1 isoforms: RASSF1A is generally regarded as a canonical tumor suppressor, whereas our in vitro and in vivo functional data consistently demonstrate that, in the context of pancreatic ductal adenocarcinoma, RASSF1C acts as a functionally oncogenic isoform that promotes tumor development and progression.

Accordingly, our findings extend the existing conceptual framework of “hypoxia/metabolic reprogramming-immune microenvironment crosstalk” by providing RASSF1C-related molecular insights that advance the understanding of how an immunosuppressive TME is established in PAAD. Previous studies have extensively documented the link between HIF-1α and the Warburg effect and have increasingly recognized the regulatory role of lactate in shaping the tumor immune milieu [58, 59]. Building on this knowledge, our study delineates a coherent line of evidence supporting the involvement of RASSF1C in hypoxia-driven metabolic-immune interactions and connects RASSF1C activity to lactate production and macrophage phenotypic reprogramming, thereby providing a more defined upstream driver and mechanistic explanation for immunometabolic regulation in PDAC.

In tumor metabolism research, the Warburg effect has long been a central focus [60, 61]. Previous studies have shown that tumor cells rely on anaerobic glycolysis under hypoxic conditions and, even in the presence of oxygen, preferentially shift toward glycolysis to generate large amounts of lactate [62]. Concurrently, HIF-1α promotes the Warburg effect under hypoxic conditions by upregulating glycolysis-related genes [63]. In this study, we found that the RASSF1C-HIF-1α axis facilitates these metabolic adaptations: upregulation of RASSF1C was accompanied by enhanced HIF-1α signaling and, under hypoxia, promoted a glycolytic phenotype and lactate production, thereby enabling metabolic alterations to be more effectively transmitted to the TME. At the same time, accumulating evidence indicates that lactate is not merely a metabolic byproduct of tumor cells but also functions as an immunoregulatory mediator involved in immune evasion [64, 65]. For example, existing studies indicate that lactate can modulate myeloid cell function and is associated with macrophage polarization toward an M2-like phenotype [66–68]. Building on this foundation, our study extends the evidence base in the context of PDAC by demonstrating that lactate accumulation driven by the RASSF1C-HIF-1α axis is accompanied by M2-like TAM activation and is linked to invasive and metastatic phenotypes [8, 69]. Therefore, compared with previous studies that have primarily centered on the “hypoxia-glycolysis-lactate-macrophage” cascade, our work highlights RASSF1C as an upstream driver in PDAC metabolic-immune interactions, providing a more precise molecular trigger and mechanistic explanation for this canonical pathway in the PDAC setting.

Compared with the substantial body of prior work that has largely focused on the paradigm in which hypoxia-, glycolysis-, or lactate-associated signals directly drive macrophage polarization [46, 65, 67, 68], the incremental innovation of this study lies in the introduction of an additional immunoregulatory node at the level of post-translational modification and protein stability, namely the UFMylation-IRF7 module. UFMylation, an emerging ubiquitin-like post-translational modification, has attracted increasing attention in recent years, with accumulating evidence implicating its roles in cellular stress responses, immune regulation, and tumor immune evasion [70, 71]. In this study, we demonstrated for the first time that lactate within the tumor-associated metabolic milieu reduces IRF7 protein stability and promotes its degradation by suppressing UFL1-mediated UFMylation of IRF7, thereby altering macrophage lipid metabolism and immune function. This finding not only provides a previously unrecognized post-translational mechanism underlying IRF7 stability control but also offers a new perspective on how metabolic signals can reprogram myeloid immune cell function through post-translational modification pathways. Although the essential role of IRF7 in innate immune responses has been well established, the mechanisms governing its stability in the TME remain poorly defined [72–74]. Accordingly, our results suggest that, beyond canonical lactate signaling pathways, UFL1/UFMylation-dependent turnover of IRF7 protein may represent a novel regulatory dimension linking tumor metabolic stress to immunosuppressive phenotypes.

In summary, this study systematically delineated the link between the RASSF1C-HIF-1α axis and metabolic reprogramming in PDAC and further showed that enhanced tumor cell glycolysis and lactate accumulation were associated with metabolic and immunophenotypic alterations in TAMs. These findings deepen the understanding of how an immunosuppressive TME is established during PAAD progression. More importantly, beyond prior studies that have primarily focused on the “hypoxia-glycolysis-lactate-macrophage” pathway, this work proposes and provides evidence that lactate can regulate IRF7 protein stability by modulating UFL1-associated UFMylation, thereby introducing a novel “post-translational modification-protein homeostasis” dimension through which metabolic signals shape myeloid immune cell function. It should be emphasized that, although this mechanism offers new molecular insights into the immunosuppressive TME in PDAC, the current evidence is largely derived from in vitro experiments and animal models and is therefore insufficient to inform immediate clinical applications directly. Future studies using models and patient cohorts that more closely reflect clinical settings will be required to systematically evaluate the reproducibility, clinical relevance, and translational potential of this pathway.

This study also has several limitations. First, there remains room to improve sample size and statistical robustness. The scRNA-seq analysis included tumor samples from only two mice, and some in vivo experiments were conducted with a group size of *n* = 5. Although such sample sizes are common in mechanistic mouse tumor studies, they may limit the ability to capture biological heterogeneity, reduce the precision of effect size estimates, and increase the risk of chance findings. To enhance the testability of our results, we included the presentation of individual data points and variance measures in the revised manuscript, reported effect sizes and confidence intervals, and reanalyzed the single-cell data using a pseudo-bulk framework at the “per mouse × cell type” level to mitigate the impact of pseudoreplication on statistical inference. Nevertheless, future studies incorporating additional biological replicates, larger animal cohorts, and independent validation datasets will be necessary to strengthen these conclusions further. Second, clinical validation requires further reinforcement. At present, human evidence is primarily derived from IHC analyses and correlation assessments using tissue microarrays, which are limited by sample size and cannot establish causality. Validation in larger patient cohorts, ideally incorporating prognostic information and treatment response data, will be essential. Third, the complexity of the immune microenvironment suggests that lactate effects may be cell-type- and context-dependent. Beyond macrophages, the impact of lactate and associated metabolic stress on other immune cell subsets and their network-level interactions remains to be elucidated. Fourth, although this study focused on the novel mechanism of UFMylation-IRF7-mediated stability control, the precise molecular pathways underlying IRF7 degradation—such as the relative contributions of proteasomal versus lysosomal pathways—as well as the substrate spectrum and upstream signaling events regulating UFMylation require more detailed functional investigation. Future work integrating patient-derived organoids, humanized mouse models, and targeted interventions along the metabolic and UFMylation axes will be important for further validating the role of this mechanism in the immunosuppressive TME of PDAC and assessing its therapeutic tractability.

## Materials and methods

### Cell culture and establishment of in vitro models

Human PAAD cell lines PANC-1 (CRL-1469) and BxPC-3 (CRL-1687), as well as the human monocytic cell line THP-1 (TIB-202), were purchased from ATCC (USA). The Panc02 mouse PAAD cell line (CL-0736) and the mouse macrophage cell line RAW 264.7 (CL-0190) were obtained from Wuhan Pricella Biotechnology Co., Ltd. PANC-1, Panc02, and RAW 264.7 cells were cultured in Dulbecco’s Modified Eagle Medium (DMEM, 11965092, Gibco, USA) supplemented with 10% fetal bovine serum (FBS) and 1% penicillin/streptomycin. BxPC-3 and THP-1 cells were cultured in RPMI-1640 (A1049101, Gibco, USA) supplemented with 10% FBS and 1% penicillin/streptomycin. For macrophage differentiation, THP-1 monocytes were treated with 50 nM phorbol 12-myristate 13-acetate (PMA, HY-18739, MedChemExpress, USA) when their density reached 1.0 × 10^5^ cells/cm^2^, and the cells were incubated in 0.4 μm cell culture inserts (CLS3412, Corning, USA) for 48 h to differentiate into macrophages [75, 76].

The Panc02-luc cell line was generated by infecting Panc02 cells with the pgm-luciferase-GFP lentiviral reporter plasmid using a standard lentiviral infection protocol, and GFP-positive cells were sorted using the Aurora CS system (Cytek Biosciences, Fremont, CA). Panc02 and Panc02-luc cells were cultured in a complete DMEM medium supplemented with 10% FBS and 100 U/mL penicillin/streptomycin.

293 T cells (CRL-3216) were purchased from ATCC and cultured in DMEM (11965092, Gibco, USA) containing 10% FBS, 10 μg/mL streptomycin, and 100 U/mL penicillin. All cells were maintained in a humidified incubator at 37 °C with 5% CO_2_ (Heracell™ Vios 160i CR CO_2_ incubator, 51033770, Thermo Scientific™, Germany) and passaged when they reached 80–90% confluence [77].

### Subcutaneous xenograft model

Male C57BL/6 J mice (age: 4–6 weeks, strain: 219) and nude mice (age: 4–6 weeks, strain: 403) were purchased from Beijing Vital River Laboratory Animal Technology Co., Ltd. (Beijing, China). The mice were housed in separate cages in an SPF-grade animal facility, with humidity maintained at 60–65% and temperature controlled at 22–25 °C. The animals were kept under a 12-h light/dark cycle, with ad libitum access to food and water. After one week of acclimatization, the animals were monitored for health status before the start of the experiment. All animal procedures were conducted in accordance with the Institutional Guidelines for Animal Care and Use.

For tumor implantation, 5 × 10^5^ PANC-1 cells were subcutaneously injected into the flanks of nude mice. Tumor size and weight were monitored every two days. Tumor dimensions were measured using a caliper, and tumor volume was calculated using the formula: Volume = 0.5 × length × width^2^. Mice were euthanized one day after the final treatment (Day 12) or when the tumor size reached 2000 mm^3^, the maximum size allowed by the Institutional Animal Care and Use Committee.

### Orthotopic tumor model

Hair from the abdominal region of C57BL/6 J mice (4–6 weeks old) was shaved using an electric razor. The skin was disinfected with povidone-iodine solution, followed by 70% ethanol. A 1-cm incision was made on the left abdominal flank, and the pancreatic body, along with the spleen, was exposed through a sterile microsurgical technique. 2 × 10^4^ Panc02-Luc cells were inoculated into the pancreas and suspended in 50 μL of DMEM medium. After verifying the absence of bleeding or leakage, the pancreas and spleen were returned to the abdominal cavity. The abdominal muscle and skin layers were sutured in succession using intermittent 4–0 absorbable sutures (Ethicon, Raritan, NJ), and the incision site was sealed with surgical adhesive (3 M, Saint Paul, MN). Throughout the procedure and postoperatively, mice were placed on a heating pad and closely monitored until fully recovered.

Tumor burden was assessed by bioluminescence intensity. Mice were intraperitoneally injected with 150 mg/kg D-luciferin (Gold Biotechnology, Olivette, MO) and anesthetized with isoflurane. Bioluminescence signals were measured using the IVIS 200 system (Perkin Elmer, Waltham, MA). On day 15, after the D-luciferin injection, the mice were euthanized and dissected to collect major organs for ex vivo imaging. Three randomly selected KPCs were used to isolate tumor tissue for single-cell suspension preparation, which was subsequently analyzed for tumor-infiltrating immune cells and subjected to scRNA-seq. The control was normal pancreatic tissue from untreated mice (Normal, *n* = 3).

In addition, mice were randomly divided into four groups (*n* = 5 per group): (1) oe-NC group (injected with stable oe-NC PANC-1 cells); (2) oe-RASSF1C group (injected with stable oe-RASSF1C PANC-1 cells); (3) Vehicle group (injected with stable oe-RASSF1C PANC-1 cells and intraperitoneally injected with saline); (4) OXM + oe-RASSF1C group (injected with stable oe-RASSF1C PANC-1 cells and intraperitoneally injected with OXM). After 10 days of tumor growth, mice/nude mice received intraperitoneal injections of 20 mg/kg OXM (every 2 days for 2 weeks) to inhibit LDH-A (lactate dehydrogenase-A) and suppress in vivo lactate production (OXM, HY-W013032, MCE, Shanghai, China) [78, 79]

### scRNA-seq and data analysis of pancreatic tissue samples

The pancreatic tumors were excised and placed in RPMI-1640 medium. After mechanical dissociation with scissors, the tissues were digested for 30 min at 37 °C in serum-free RPMI-1640 containing deoxyribonuclease I (0.3 mg/mL^−1^, Sigma-Aldrich) and TL Liberase (0.25 mg/mL^−1^, Roche). The resulting cell suspension was filtered through a 40 μm cell filter (BD Biosciences) to obtain a single-cell suspension. Single cells were captured using the C1 Single-Cell Auto Prep System (Fluidigm, Inc., South San Francisco, CA, USA). After capture, cells were lysed within the chip to release mRNA. cDNA was then synthesized by reverse transcription. The cDNA from lysed, reverse-transcribed cells was preamplified on the microfluidic chip to facilitate subsequent sequencing. Amplified cDNA was used for library construction and single-cell sequencing on the HiSeq 4000 Illumina platform (parameters: paired-end reads, read length 2 × 75 bp, approximately 20,000 reads per cell).

Data quality control was performed based on the criteria 200 < nFeature_RNA < 5000 and percent.mt <25, followed by selecting the top 2000 most highly variable genes with the greatest variance.

### t-SNE clustering and cell annotation

To reduce the dimensionality of the scRNA-Seq dataset, we performed principal component analysis (PCA) based on the top 2000 most variable genes. Using the ElbowPlot function in the Seurat package, the first 14 principal components were selected for downstream analysis. The FindClusters function in Seurat was used to identify the major cell subpopulations, with a resolution set to the default value (resolution = 0.5). Nonlinear dimensionality reduction was then performed using t-SNE on the scRNA-Seq data. Finally, cell types were annotated using known cell-line-specific marker genes and cross-referenced with the CellMarker online database.

### PAAD mouse tumor tissue sample ST data download and analysis

ST dataset GSE244534 was downloaded from the Gene Expression Omnibus (GEO) database (https://www.ncbi.nlm.nih.gov/gds). This dataset includes ST sequencing data from tumor sections of 4 PAAD mouse models. Using Seurat’s anchor-based integration pipeline, we integrated the scRNA-seq data mentioned above with the 10× Visium ST data from GSE244534. This approach enabled the transfer of cell type annotations from scRNA-seq to ST. The predicted cell types from Seurat were then loaded into the SPOTlight R package for annotation and visualization of cell types at each spatial location.

### Silencing and overexpression lentiviral vector construction

Based on GeneBank, potential short hairpin RNA (shRNA) target sequences for human cDNA were analyzed. We designed two sequences targeting RASSF1C, HIF-1α, or IRF7 and a negative control (sh-NC) with no interfering sequence. The primer sequences are listed in Table S1, and the oligonucleotides were synthesized by GenePharma® (Shanghai, China). Lentiviral packaging systems were constructed using the pLKO.1 vector (a lentiviral gene silencing vector). The packaging virus and target vector were co-transfected into 293 T cells (at 80–90% confluence) using Lipofectamine 2000. After 48 h of incubation, the supernatant was collected, filtered, and centrifuged to isolate viral particles. Viral titer was determined by measuring the growth of viruses in the logarithmic phase. Genechem (Shanghai) constructed and packaged overexpression lentiviruses, using the pLenti-CMV-GFP-puro vector. Dimethyloxalylglycine (DMOG, MCE, Shanghai, China), a PHD inhibitor, stabilized HIF-1α protein accumulation.

### Flow cytometry (FCM) analysis

Macrophage phenotypes were assessed by FCM using anti-CD206 (ab270647, Abcam, Cambridge, UK) and anti-CD163 (ab314947, Abcam, Cambridge, UK) antibodies. Cells were fixed overnight with 1% paraformaldehyde (PFA) and analyzed the following day. The cells were analyzed using an LSRFortessa™ cell analyzer (BD Biosciences, USA) and the data were processed with FlowJo software (Ashland, OR, USA).

### Immunofluorescence staining

Cells were fixed with 4% PFA at room temperature for 15 min and then blocked with 3% bovine serum albumin (BSA) at 37 °C for 30 min to prevent non-specific staining. After overnight incubation with the primary antibody, the cells were washed 3 times with phosphate-buffered saline (PBS) for 3 min each. Subsequently, the cells were incubated with the secondary antibody at room temperature for 2 h. DAPI was used to counterstain the cell nuclei. All immunofluorescence-stained slides were examined under a fluorescence microscope (Olympus Corporation, Japan). The primary antibodies used were as follows: mouse anti-RASSF1C (NBP2-03644, Bio-Techne China Co. Ltd, Shanghai, China), F4/80 (ab60343, Abcam, Cambridge, UK), and rabbit anti-CD206 (NBP1-90020, Bio-Techne China Co. Ltd, Shanghai, China). The secondary antibodies were Alexa Fluor 647-conjugated goat anti-mouse IgG (ab150159, Abcam, Cambridge, UK) or Alexa Fluor 488-conjugated affinity-purified goat anti-rabbit IgG (ab150081, Abcam, Cambridge, UK). All antibodies were diluted according to the manufacturer’s instructions.

### Seahorse XF mitochondrial/glycolytic stress test analysis

The mitochondrial respiration and glycolytic activity of cells were assessed using the Seahorse XF Mitochondrial Stress Test and Glycolysis Stress Test Kits (103015-100 and 103020-100, Seahorse Bioscience, USA). Briefly, cells were seeded into XFe96 cell culture microplates and incubated overnight. For the mitochondrial stress test, the following reagents were added: oligomycin (Oligo) in well A (final concentration 1 μM), FCCP in well B (final concentration 0.5 μM), and rotenone and antimycin A in well C (final concentration 0.5 μM). For the glycolysis stress test, the reagents included glucose in well A (final concentration 10 mM), Oligo in well B (final concentration 1 μM), and 2-deoxyglucose (2-DG, Seahorse Bioscience, 103020-100) in well C (final concentration 50 mM). The analysis was performed using the Agilent Seahorse XFe96, and the results were analyzed with Wave Desktop and Report Generator software.

### Glucose uptake assay

Cells were seeded in 6-well plates (2 × 10^5^ cells/well) and incubated at 37 °C for 24 h. Before testing, the growth medium was replaced with a glucose-free medium to starve the cells of glucose for 2–3 h. Afterward, the cells were incubated with fresh medium containing either 2-NBDG (A22189, Thermo Fisher Scientific Inc., Shanghai, China) or without it. The cells were incubated at 37 °C for an additional 45 min, washed with PBS, and digested into single cells for analysis.

### Measurement of pyruvate, lactate, and ATP

Pyruvate, lactate, and ATP production in cells were measured using the Pyruvate Assay Kit (S0299S, Beyotime, Shanghai, China) and the ATP Assay Kit (S0026, Beyotime, Shanghai, China), following the manufacturer’s protocols. Lactate in the cell CM was quantified using the Vitros 250 chemical analyzer (Johnson & Johnson, USA) at the University of Texas Southwestern Metabolic Phenotyping Center.

### ELISA assay

The expression levels of TNF-α (PT518 for human, PT512 for mice, Beyotime, Shanghai, China) and IL-10 (PI528 for human, PI522 for mice, Beyotime, Shanghai, China) in cell culture supernatant were measured using ELISA kits according to the manufacturer’s instructions. Absorbance (A) values at 450 nm were measured within 3 min using a Synergy 2 plate reader (BioTek, USA). The concentration of the target proteins was calculated by generating a standard curve from a regression equation, with the standards’ concentrations on the x-axis and absorbance on the y-axis. The sample absorbance values were then substituted to determine the protein concentration.

### CCK-8 assay

Cell proliferation was assessed using the CCK-8 kit (40203ES60, Yeasen, Shanghai, China). Cells in the logarithmic growth phase were adjusted to a concentration of 5 × 10^4^ cells/mL and seeded into 96-well plates. Each well was filled with 100 μL of CM, and cells were incubated for 0, 12, 24, or 48 h. After discarding the supernatant, fresh CM was added, followed by 10 μL of CCK-8 reagent. After incubating at 37 °C for 2 h, absorbance (A) was measured at 450 nm using a Multiskan FC plate reader (51119080, Thermo Fisher Scientific, USA). Three replicate wells were used for each condition, and the average value was calculated. The experiment was repeated three times.

### Transwell assay for cell migration and invasion

ECM gel (E1270, Sigma-Aldrich, Germany) was added to the upper chamber of a 24-well Transwell plate with 8 μm pores. The plate was incubated at 37 °C for 30 min to solidify the gel. After 48 h of transfection, cells were resuspended in a serum-free medium at a concentration of 1 × 10^5^ cells and seeded into the upper chamber. Each well was loaded with 200 μL of cell suspension (2 × 10^4^ cells). The lower chamber was filled with 800 μL of medium containing 20% FBS. After 24 h of incubation at 37 °C, the Transwell plate was removed and washed twice with PBS. Cells were then fixed with formaldehyde for 10 min and washed thrice with water. The cells were stained with 0.1% crystal violet at room temperature for 30 min, followed by two washes in PBS. Cells on the upper surface were removed with a cotton swab. The invaded cells were photographed under an inverted microscope (CKX53, Olympus, Japan) and counted using ImageJ software to assess the invasive capacity of the cancer cells. No ECM gel was applied to the Transwell plate for the migration assay, and all other steps were identical to the invasion assay.

### Extraction of lipid metabolites

Metabolite Extraction: Cells were seeded in culture dishes and allowed to grow until confluence. Once confluent, they were digested with trypsin-EDTA (25200072, Gibco, USA) and washed with PBS. The washed cells, or pretreated PAAD tissue, were then freeze-dried. 100 μL of pre-chilled ethanol/acetonitrile/water (2:2:1, v/v) was added to the lyophilized samples, followed by vortex mixing and low-temperature sonication for 30 min. The samples were placed at −20 °C for 10 min and centrifuged at 4 °C, 14,000 *g* for 20 min. The supernatant was further vacuum-dried and reconstituted with 100 μL of acetone/water (1:1, v/v). After vortex mixing, the samples were centrifuged again at 4 °C, 14,000 *g* for 15 min, and the resulting supernatant was used for subsequent analysis.

High-Performance Liquid Chromatography (HPLC) Conditions: The samples were separated using a Hydrophilic Interaction Liquid Chromatography (HILIC) column on an ultra-high-performance liquid chromatography (UHPLC) system. The column temperature was 40 °C, with a flow rate of 0.4 mL/min and an injection volume of 2 μL. The mobile phase A consisted of water with 25 mmol/L ammonium acetate and 25 mmol/L ammonium hydroxide, while phase B was acetonitrile. The gradient elution was as follows: 0–0.5 min, 95% B; from 0.5 to 7 min, B was linearly reduced from 95 to 65%; from 7 to 8 min, B was linearly reduced from 65 to 40%; from 8 to 9 min, B was maintained at 40%; and finally, from 9 to 12 min, B was increased from 40 to 95%, maintaining 95% until the end of the run.

Q-TOF Mass Spectrometry Conditions: The ESI source was operated with an ion source gas I and II at 50, a shield gas at 30, and a source temperature of 500 °C. In both negative and positive ion modes, the ion spray voltage was set to ±5500 V. Data acquisition was performed in information-dependent acquisition (IDA) mode with high sensitivity, scanning product ions. The collision energy was fixed at 35 ± 15 eV, and the declustering potential was set to ±80 V in both ion modes.

### LC-MS analysis of total lipid metabolite content

Raw mass spectrometry data (wiff.scan files) were converted to .mzXML format using ProteoWizard MSConvert. Peak alignment, retention time correction, and peak area extraction were performed using XCMS. CAMERA (a collection of algorithms for Metabolite Profile Annotation) was used to annotate isotopes and adducts. Compound identification was achieved for all lipid metabolites and other metabolites by matching m/z values (<10‰) and MS/MS spectra with the mzCloud, mzVault, and Masslist databases.

### Lipid content measurement

Cell lipid content was determined using BODIPY 493/503 (C2053S, Beyotime, Shanghai, China). Briefly, cells were washed with PBS and stained with BODIPY 493/503 in PBS (without FBS or fatty acids) for 15 min at room temperature. Staining was then analyzed immediately using confocal microscopy and FCM.

### RT-qPCR detection of relative gene expression

Total RNA was extracted from tissues or cells using Trizol reagent (15596026, Invitrogen, USA). The RNA concentration and purity were assessed using a NanoDrop LITE spectrophotometer (ND-LITE-PR, Thermo Scientific™, Germany) at 260/280 nm. Complementary DNA (cDNA) was synthesized from the extracted RNA using the PrimeScript RT Reagent Kit with gDNA Eraser (RR047Q, TaKaRa, Japan). RT-qPCR was performed using SYBR Green PCR Master Mix reagents (4364344, Applied Biosystems, USA) on an ABI PRISM 7500 Sequence Detection System (Applied Biosystems).

TaKaRa (Table S2) synthesized primers for each gene, with GAPDH as the internal control. Relative gene expression was analyzed using the 2^−ΔΔCt^ method, where ΔΔCt = [(average Ct value of the target gene in the experimental group − average Ct value of the housekeeping gene in the experimental group) − (average Ct value of the target gene in the control group − average Ct value of the housekeeping gene in the control group)]. All RT-qPCR experiments were performed in triplicate.

### Western blot

Tissues or cells were collected and lysed using enhanced RIPA lysis buffer with protease inhibitors (P0013B, Beyotime, Shanghai). Protein concentrations were determined using the BCA Protein Assay Kit (P0012, Beyotime, Shanghai). Proteins were separated by 10% SDS-PAGE and transferred to PVDF membranes. Membranes were blocked with 5% BSA at room temperature for 2 h to prevent non-specific binding. Diluted primary antibodies were added and incubated at room temperature for 1 h. Primary antibody details are provided in Table S3. After washing, HRP-conjugated secondary antibodies (goat anti-rabbit IgG, ab6721, 1:2000, Abcam, UK, or goat anti-mouse IgG, ab6785, 1:1000, Abcam, UK) were added and incubated for 1 h at room temperature. All antibodies were diluted according to the manufacturer’s instructions. Western Blot detection was performed using Pierce™ ECL Western Blot Substrate (32209, Thermo Scientific™, Germany), with equal amounts of solutions A and B mixed in the darkroom, applied to the membrane, and imaged using a gel imager. Images were captured using a Bio-Rad imaging system (USA), and band intensities were quantified using ImageJ. GAPDH/Tubulin served as the internal control. Each experiment was repeated three times.

### ChIP

Cells were cross-linked with 1% formaldehyde at room temperature for 10 min to capture DNA-protein interactions. Following crosslinking, the samples were sonicated to randomly fragment the DNA into appropriate-sized pieces (10 s on, 10 s off, for 15 cycles). The samples were centrifuged at 12,000 *g* at 4 °C, and the supernatant was divided into two tubes. One tube was incubated with a negative control antibody (IgG), and the other with a specific antibody against the target protein HIF-1α, overnight at 4 °C (antibody details are provided in Table S3). The DNA-protein complexes were precipitated using Protein Agarose/Sepharose, followed by brief centrifugation and washing of non-specific complexes. The crosslinks were reversed by overnight incubation at 65 °C, and the DNA was purified by phenol/chloroform extraction. ChIP-qPCR products were analyzed qualitatively by 3% agarose gel electrophoresis.

### PLA

Cells were seeded onto chamber slides and allowed to adhere before being exposed to Hox at various times. After Hox treatment, cells were fixed using a 1:1 mixture of acetone and methanol. The experiment was then conducted according to the manufacturer’s instructions provided with the Duolink® Proximity Ligation Assay Kit (Sigma-Aldrich, USA).

Antibodies targeting HIF-1α (1:500, ab179483, Abcam, UK) and RASSF1C (1:500, NBP2-03644, Bio-Techne, Shanghai, China) were used to detect interactions between these two proteins. After fixation and treatment, antibody incubation, ligation, and amplification were performed using the Duolink® kit to generate detectable fluorescent signals. Fluorescent images were captured using a fluorescence microscope (Olympus Corporation, Japan) to analyze the proximity interactions between HIF-1α and RASSF1C.

### Co-IP

Co-IP experiments were performed using the classic magnetic bead-based IP/Co-IP Kit (88804, Thermo Fisher Scientific Inc., Shanghai, China). Cells were cultured in 100 mm dishes to 80% confluence and transfected with various plasmids (RASSF1C-Flag, IRF7-Flag, RPL26-cFlag, ASC1-cFlag, or HIF-1α plasmids) using Turbofect Transfection Reagent (R0531, Thermo Fisher Scientific Inc., Shanghai, China). After 6 h of transfection, cells were exposed to either Hox (24 h) or normoxic conditions (Nox), followed by lysis with Co-IP lysis buffer (Thermo Scientific). To assess post-translational modifications such as proline hydroxylation and K48-linked ubiquitination, cells were pre-treated with MG132 (HY-13259, MCE, Shanghai, China) for 30 min before hypoxic exposure. After 6 h of Hox treatment, cells were lysed in Co-IP lysis buffer.

After centrifugation, the supernatant was used for Co-IP. Equal amounts of protein were incubated overnight at 4 °C with anti-Flag, anti-HIF-1α, or IgG control antibodies (antibody details provided in Table S3). Protein-antibody complexes were then incubated with Protein G Sepharose beads for 3 h at room temperature. After incubation, non-specifically bound proteins were removed by washing the beads multiple times with PBS containing 0.1% Tween-20. Finally, the beads were mixed with SDS sample buffer, SDS-PAGE-separated proteins, and Western Blot-detected target proteins.

### GST pull-down

IRF7 and UFL1 were cloned into the pGEX-6p-2 vector (HG-VYA0226, Changsha Abiowell Biotechnology Co., Ltd., Changsha, China). The recombinant IRF7 and UFL1 proteins, tagged with GST, were purified using the Escherichia coli BL21 expression system. The GST Pull-Down assay was performed by incubating 2 mg of purified GST-tagged recombinant proteins (immobilized on glutathione-Sepharose resin, 17-0756-05, GE Healthcare, USA) with UFL1 or IRF7 proteins overexpressed in Raw264.7 cells. The incubation was carried out at 4 °C for 3 h.

After incubation, the Pull-Down products were washed 5 times with NETN buffer (Nonidet P-40, EDTA, Tris, NaCl) to remove unbound proteins. Next, 2×SDS loading buffer was added, and the samples were boiled at 95 °C for 5 min to fully denature the proteins. Finally, the samples were separated by SDS-PAGE and analyzed by Western Blot to assess the GST Pull-Down results.

### In vitro UFMylation assay

Raw264.7 cells were transfected with the specified plasmids, and 36 h after transfection, the cells were treated overnight with 10 mM MG132. The cells were then lysed using Buffer A (6 M guanidine hydrochloride, 0.1 M Na_2_HPO_4_/NaH_2_PO_4_, and 10 mM imidazole, pH 8.0). After sonication, the lysate was incubated with Ni-NTA beads (88832, Thermo Fisher Scientific Inc., Shanghai, China) for 3 h at room temperature. His Pull-Down products were washed sequentially with the following buffers: two washes with Buffer A, two washes with Buffer A/TI (a 1:3 mixture of Buffer A and Buffer TI), and one wash with Buffer TI (25 mM Tris-HCl, 20 mM imidazole, pH 6.8). Finally, the Pull-Down proteins were separated by SDS-PAGE and detected by Western Blot with specific antibodies, and the signal was detected using the ECL method.

### IP analysis

Raw264.7 cells were treated with 20 mM MG132 for 6 h and then collected. The cells were lysed in denaturing buffer (50 mM Tris, 1% SDS, 0.5 mM EDTA, 1 mM dithiothreitol (DTT), pH 7.5) and incubated at 95 °C for 20 minutes to ensure complete denaturation. The denatured cell lysate was sonicated and then diluted 10-fold with EBC buffer. UFL1, IRF7, ubiquitin-like modifier 1 (UFM1), PRODH2, and K48 ubiquitin antibodies were added, and the mixture was incubated overnight at 4 °C (antibody details are provided in Table S3). Following incubation, 50 µL of Protein A Sepharose beads (20334, Thermo Fisher Scientific Inc., Shanghai, China) were added, and the mixture was further incubated at 4 °C for 1 h. The immunocomplexes were washed four times with NETN buffer, and the proteins were separated by SDS-PAGE. Western Blot analysis was then performed to detect the protein interactions.

### Luciferase assay

The Dual-Luciferase® Reporter Assay System (Promega, E1910) was used to measure luciferase activity. To validate the binding of HIF-1α to the RASSF1C promoter, cells were seeded in 48-well plates and co-transfected with the pcDNA3.1-HIF-1α plasmid and a luciferase reporter plasmid containing the RASSF1C promoter region (hypoxia binding sites (HBS)1 or HBS2) (all plasmids synthesized by HANBIO, China). The pRL-TK vector (Promega, LM1568) was used to normalize luciferase activity. After 48 h of transfection, cells were lysed, and luciferase activity was measured.

### High-throughput sequencing and analysis

Transcriptome analysis was performed on Panc02 cells under Nox and Hox (*n* = 4 per group) and on lactate-stimulated Raw264.7 macrophages with IRF7 knockdown (sh-IRF7+Lactate) or a negative control (sh-NC+Lactate) (*n* = 4 per group). Total RNA was extracted using the Total RNA Extraction Kit (12183555, Invitrogen, USA), and RNA concentrations were quantified by measuring optical density (OD) values. The integrity of RNA samples was assessed using agarose gel electrophoresis. High-quality RNA was reverse-transcribed into cDNA to construct RNA libraries sequenced using the Illumina NextSeq 500 platform. Base calling was used to convert the raw image data into raw reads. Sequencing adaptors and low-quality reads were removed using cutadapt, and the remaining high-quality reads were referred to as “clean reads.” These reads were aligned to the human reference genome using Hisat2, and gene expression was quantified using R to generate an expression matrix.

DEGs were identified using the limma R package with thresholds of |log_2_FC | > 1 and adj.*p*.Val < 0.01. Volcano plots were generated with the ggplot2 package, and heatmaps were created using pheatmap. Gene Ontology (GO) and KEGG analyses were performed using the Xiantao Academic Database (https://www.xiantaozi.com/). The interaction sites between HIF-1α and the RASSF1C promoter were predicted using the JASPAR database (https://jaspar.genereg.net/). Immune infiltration analysis was conducted with TIMER v2.0 (http://timer.cistrome.org), and the expression of IRF7 and UFL1 in PAAD was analyzed using the Starbase database (https://rnasysu.com/encori/).

### Human PDAC tissue microarray and IHC analysis

A commercial human pancreatic ductal adenocarcinoma (PDAC) tissue microarray (TMA) containing 20 tumor specimens (vendor: Shanghai Xinchao Biotech Co., Ltd., Xinchao Biotech; catalog no.: Hpa-PanA150CS-01) was used to characterize the expression profiles of RASSF1C–HIF-1α–UFL1–IRF7–related molecules in clinical samples. TMA sections were deparaffinized in xylene and rehydrated through a graded ethanol series, followed by heat-induced antigen retrieval in pH 6.0 citrate buffer (Servicebio, G1202) using a pressure cooker. Endogenous peroxidase activity was quenched with 3% H₂O₂ (Solarbio, H8030), and sections were blocked with 5% BSA (Solarbio, A8020) for 30 min, then incubated overnight at 4 °C with the following primary antibodies: RASSF1C (Proteintech, 15387-1-AP, 1:200), HIF-1α (Abcam, ab2185, 1:200), UFL1 (Proteintech, 24086-1-AP, 1:150), and IRF7 (CST, 4920, 1:150). After PBS washes, sections were incubated with an HRP-conjugated secondary antibody (Servicebio, G1211) for 1 h at room temperature, developed using a DAB kit (Servicebio, G1211-200T), and counterstained with hematoxylin.

Stained slides were digitized using a Leica Aperio CS2 scanner. Quantification was performed using QuPath v0.4.3 and ImageJ/Fiji (NIH). To minimize confounding due to tissue composition, each core was manually annotated in QuPath based on morphologic features and subdivided into two compartments: tumor epithelium and stroma/immune infiltration regions (excluding necrosis, hemorrhage, and non-tissue areas). Under the Brightfield (H-DAB) setting, stain separation was performed followed by cell detection and positive cell detection. Intensity thresholds for each antibody were determined using negative/background signals and representative fields and then consistently applied across all samples. For each compartment, the percentages of weak (1 + ), moderate (2 + ), and strong (3 + ) positive cells (P1, P2, P3) were calculated, and H-scores were computed as H-score = 1×P1 + 2×P2 + 3×P3 (range 0–300). The final H-score for each case was summarized as the mean (or median, depending on the prespecified analysis plan) across multiple fields/annotated regions. ImageJ/Fiji was primarily used for H-DAB color deconvolution and threshold verification in representative fields to confirm consistency with QuPath-based quantification.

After compartment-specific quantification, Spearman’s rank correlation was used to assess correlations of H-scores among markers within the tumor epithelium and within the stroma/immune infiltration compartments, respectively.

### Construction and stable expression of the catalytically inactive UFL1 mutant (C307A)

The catalytically inactive UFL1 mutant (C307A) was generated by site-directed mutagenesis. Based on previously reported catalytic cysteine residues of UFL1, Cys300 [34, 80, 81], primers were designed to substitute this residue with alanine (Cys307→Ala). Mutagenesis was performed using the QuikChange II Site-Directed Mutagenesis Kit (Agilent Technologies, Cat. No. 200523) according to the manufacturer’s instructions, and the correct nucleotide and amino acid substitutions were verified by Sanger sequencing (Fig. S1). The validated UFL1-C307A construct and UFL1-WT were subsequently subcloned into FLAG-tagged lentiviral expression vectors.

For lentivirus production, psPAX2 (Addgene plasmid #12260) and pMD2.G (Addgene plasmid #12259) were co-transfected with the corresponding lentiviral vectors into HEK293T cells using Lipofectamine 2000 (Thermo Fisher Scientific, Cat. No. 11668019) following the manufacturer’s protocol. Viral supernatants were collected 48 h after transfection, filtered through 0.45 μm membranes, and used to infect RAW264.7 cells. Polybrene (hexadimethrine bromide; Sigma-Aldrich, Cat. No. H9268) was added during infection at a final concentration of 8 μg/mL to enhance transduction efficiency. At 24 h post-infection, the medium was replaced with fresh CM, and infected cells were selected with puromycin dihydrochloride (Sigma-Aldrich, Cat. No. P9620) at a final concentration of 2 μg/mL for 72 h. After selection, surviving cells were collected to establish stable cell populations expressing either UFL1-WT or UFL1-C307A for subsequent functional assays. Western blot analysis using an anti-FLAG antibody to detect exogenous UFL1 was performed, and band intensities were quantified and normalized to GAPDH, confirming comparable expression levels of UFL1-WT and UFL1-C307A and thereby excluding expression-level differences as a confounding factor in downstream functional analyses.

### CRISPR/Cas9-mediated UFM1 gene knockout

A specific single-guide RNA (sgRNA) targeting exon 2 of the mouse Ufm1 gene (sgUfm1-ex2; target sequence: 5′-CTTTAAAATCACGTTGACGT-3′) was designed and cloned into the lentiCRISPRv2 vector (Addgene plasmid #52961). A non-targeting sgRNA (sgCtrl; 5′-GCTTACGTCAGGATCGTTAC-3′) was used as a negative control. For lentivirus production, psPAX2 (Addgene plasmid #12260) and pMD2.G (Addgene plasmid #12259) were co-transfected with either lentiCRISPRv2-sgUfm1 or lentiCRISPRv2-sgCtrl into HEK293T cells using Lipofectamine 2000 (Thermo Fisher Scientific, Cat. No. 11668019) according to the manufacturer’s instructions. Viral supernatants were collected 48 h after transfection, filtered through 0.45 μm membranes, and used to infect RAW264.7 cells in the presence of polybrene (Sigma-Aldrich, Cat. No. H9268) at a final concentration of 8 μg/mL to enhance transduction efficiency. At 24 h post-infection, the medium was replaced with fresh CM, and puromycin dihydrochloride (Sigma-Aldrich, Cat. No. P9620) was added at a final concentration of 4 μg/mL for 5 days to obtain puromycin-resistant cell populations.

Stable clones were subsequently generated by single-cell dilution. Genomic PCR amplification was performed using primers flanking the target site, and PCR products were subjected to Sanger sequencing followed by TIDE/ICE analysis to assess indel formation; when necessary, PCR products were TA-cloned and sequenced to obtain allelic information, thereby confirming insertion/deletion mutations at the target locus. In parallel, UFM1 protein levels were examined by Western blot to validate knockout efficiency. At least two independent Ufm1-knockout clones exhibiting consistent phenotypes were obtained and used for subsequent functional experiments; unless otherwise specified, clones with markedly reduced UFM1 protein expression were selected. Cells infected with lentiCRISPRv2-sgCtrl and subjected to the same selection procedures served as negative controls.

### CHX chase assay for assessing IRF7 protein stability

To evaluate IRF7 protein stability, macrophages stably expressing UFL1-WT, UFL1-C307A, or UFM1-KO were seeded and cultured to the logarithmic growth phase. These cells were primarily RAW264.7 cells (ATCC® TIB-71™), unless otherwise specified in the Fig. legends. Cells were then treated with CHX (50 μg/mL; Sigma-Aldrich, Cat. No. C7698) and harvested at 0, 2, 4, 6, and 8 h after treatment [82]. Under these conditions and within this time frame, no significant cell death was observed, as assessed by the trypan blue exclusion assay (0.4% Trypan Blue Solution; Gibco, Cat. No. 15250061) (Fig. S2A–C).

Cells were lysed in ice-cold RIPA lysis buffer (RIPA Lysis and Extraction Buffer; Thermo Fisher Scientific, Cat. No. 89900) supplemented with a protease inhibitor cocktail (cOmplete™ Protease Inhibitor Cocktail; Roche, Cat. No. 11836153001) and phosphatase inhibitors (PhosSTOP™; Roche, Cat. No. 4906845001). Lysates were vortexed and clarified by centrifugation at 4 °C to remove cellular debris, and the supernatants were subjected to SDS-PAGE and Western blot analysis to determine IRF7 and GAPDH protein levels.

Western blot bands were quantified by densitometry using ImageJ (NIH). IRF7 signals were first normalized to GAPDH and then expressed relative to the normalized value at the 0 h time point, which was set to 100%. Nonlinear regression analysis using a single-exponential decay model was performed in GraphPad Prism (GraphPad Software) to calculate the IRF7 protein half-life (t½). The CHX chase assay was conducted in at least three independent experiments, and t½ values derived from nonlinear regression were used for statistical analysis.

For proteasome inhibition assays, cells were pretreated with MG132 (10 μM; Selleckchem, Cat. No. S2619) for 4 h before CHX addition, and MG132 was maintained throughout the subsequent CHX chase period to determine whether IRF7 degradation was proteasome dependent. Corresponding vehicle controls (an equal volume of DMSO; Sigma-Aldrich, Cat. No. D2650) were included to exclude potential solvent effects [82–84].

### Detection of IRF7 UFMylation

IRF7 UFMylation was examined under denaturing conditions. Cells (macrophages expressing UFL1-WT, UFL1-C307A, or UFM1-KO) were lysed in 1% SDS lysis buffer [50 mM Tris-HCl (pH 7.5), 150 mM NaCl, and 1 mM EDTA] supplemented with a protease inhibitor cocktail (cOmplete™ Protease Inhibitor Cocktail; Roche, Cat. No. 11836153001), phosphatase inhibitors (PhosSTOP™; Roche, Cat. No. 4906845001), and 20 mM N-ethylmaleimide (NEM; Sigma-Aldrich, Cat. No. E3876), followed by heating at 95 °C for 10 min. Lysates were then diluted tenfold with lysis buffer containing 1% NP-40 (with the same buffer composition and continuous supplementation of protease and phosphatase inhibitors and 20 mM NEM) to reduce the final SDS concentration to approximately 0.1%. After centrifugation at 4 °C to remove insoluble material, the supernatants were used for IP.

For each sample, approximately 500–1000 μg of total protein was incubated with an anti-IRF7 antibody (Cell Signaling Technology, Cat. No. 4920) at 4 °C with rotation for 4 h to overnight, followed by incubation with Protein A/G agarose beads (Pierce™ Protein A/G Agarose; Thermo Fisher Scientific, Cat. No. 20421) for an additional 2 h. Immunoprecipitates were washed three to five times with wash buffer containing 0.1% SDS and 0.5% NP-40, supplemented with 20 mM NEM and protease inhibitors, and were finally eluted with 2× SDS sample buffer. After heating at 95 °C for 5 min, samples were subjected to SDS-PAGE and Western blot analysis. Following membrane transfer, IRF7 UFMylation levels were detected using an anti-UFM1 antibody (Proteintech, Cat. No. 10275-2-AP). The same immunoprecipitates were subsequently probed with a K48-linked ubiquitin-specific antibody (Cell Signaling Technology, Cat. No. 12805) to assess K48-linked ubiquitination of IRF7 and to compare differences among the UFL1-WT, UFL1-C307A, and UFM1-KO conditions. When necessary, cells were pretreated with MG132 (10 μM; Selleckchem, Cat. No. S2619) for 4–6 h before lysis to prevent excessive IRF7 degradation. At least 5–10% of the total cell lysate was loaded as an input control, and species-matched IgG was used as a negative control for IP.

### Statistical analysis

All statistical analyses were conducted using R software (version 4.2.1) in the RStudio integrated development environment (version 2022.12.0-353). Perl (version 5.30.0) was used for file processing, and GraphPad Prism (version 9.0) was employed for data visualization. Quantitative data were expressed as mean ± standard deviation (SD). Independent-samples t-tests were used for comparisons between two groups, while one-way analysis of variance (ANOVA) was used for comparisons among multiple groups. For comparisons across multiple time points within groups, a two-way ANOVA was used. Post hoc tests were performed using the Bonferroni correction. A significance threshold of *p* < 0.05 was applied to all analyses.

## Supplementary information

supplementary figures and legends

Western Blot

Supplementary Tables

## Data Availability

All data can be provided as needed.
