# Supplementary material for: The RASSF1C-HIF-1α axis drives macrophage lipid metabolism to promote pancreatic cancer
Source: Cell Death Dis. 2026 Mar 30;17(1):430. doi: 10.1038/s41419-026-08609-0 (PMC13156295; doi:10.1038/s41419-026-08609-0)
Supplement: Supplementary file 1 — supplementary figures and legends [file 41419_2026_8609_MOESM1_ESM.pdf]

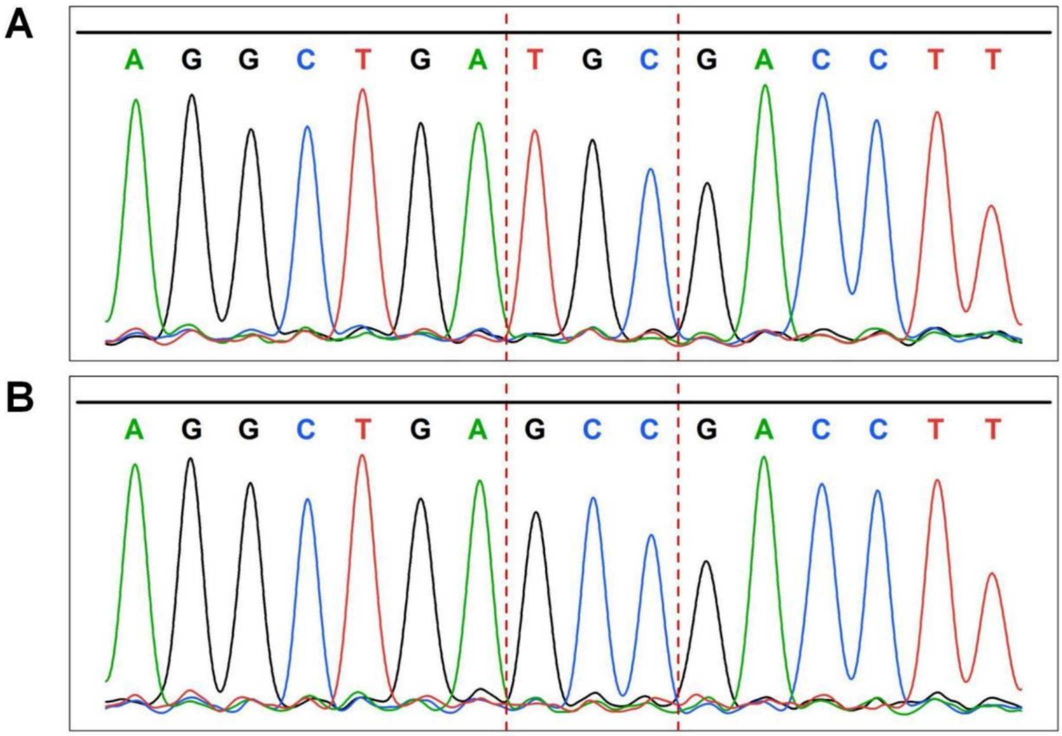


**Figure S1. Sanger sequencing confirmation of the UFL1 catalytic cysteine-to-alanine mutation.**

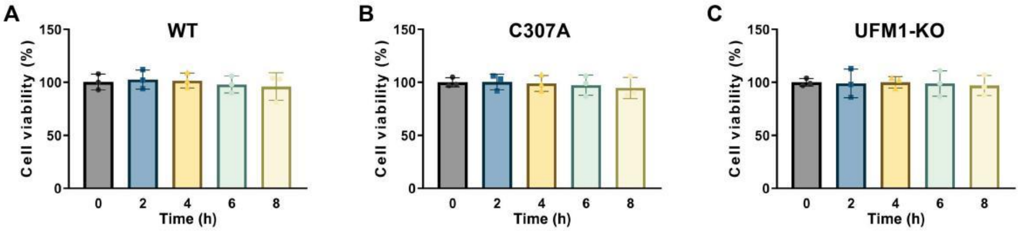


**Figure S2. Assessment of RAW264.7 cell viability under CHX chase conditions (Trypan blue exclusion assay).**

Note: (A) UFL1-WT cells; (B) UFL1-C307A cells; (C) UFM1-KO cells. After treatment with CHX (50 μg/mL), cells were subjected to 0.4% Trypan blue exclusion counting at 0, 2, 4, 6, and 8 h. Cell viability (%) was calculated as the number of viable cells divided by the total number of viable plus dead cells × 100%. Bar graphs represent the mean ± SEM from three independent experiments (n = 3). Statistical analysis was performed using one-way ANOVA (compared with the 0 h time point),

and $p < 0.05$ was considered statistically significant.

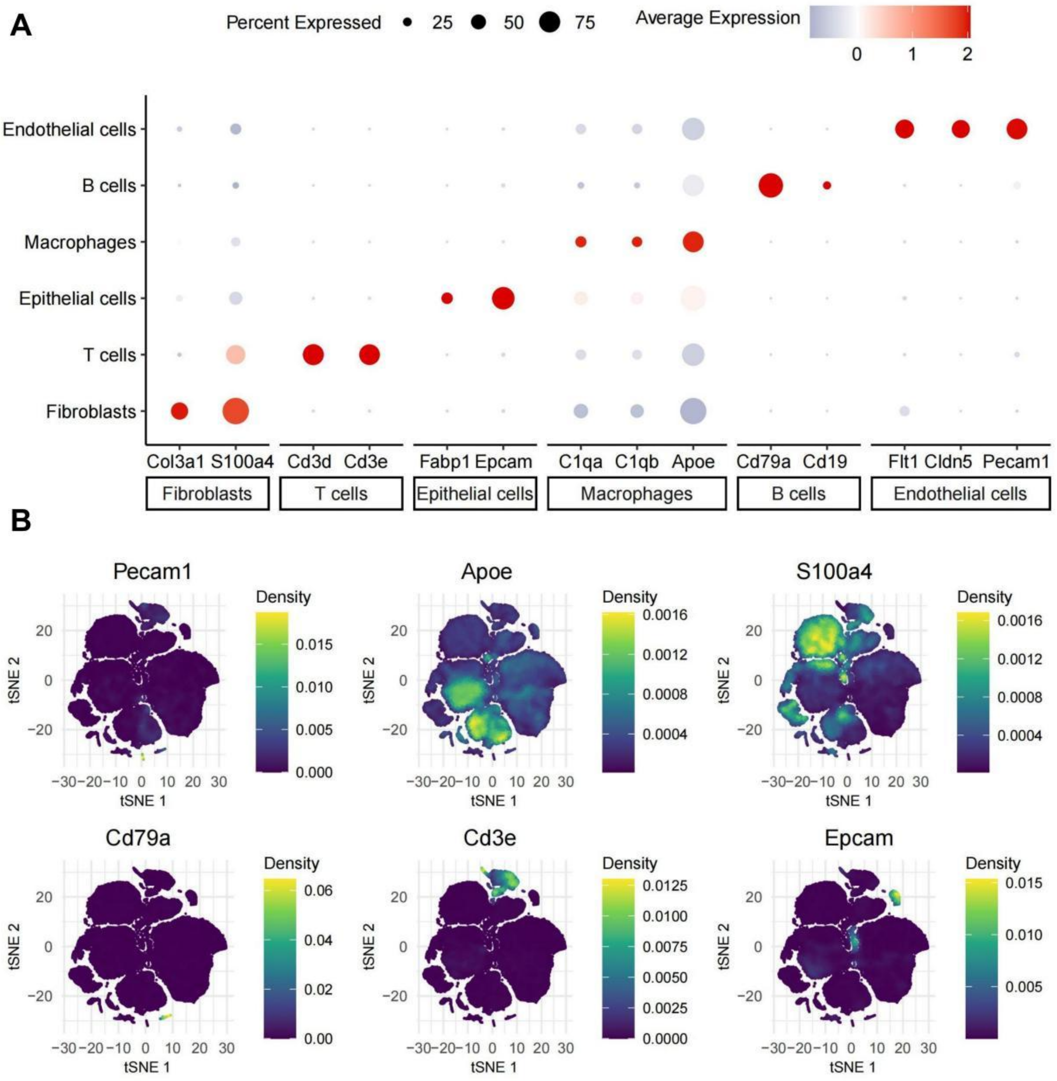


**Figure S3. Cell type-specific marker gene expression and spatial distribution analysis.**

Note: (A) Dot plot showing cell type-specific gene expression. Each dot represents the expression level of a given gene in the corresponding cell type (color intensity) and the proportion of expressing cells (dot size). (B) t-SNE plots illustrating the expression density of representative marker genes (Pecam1, Apoe, S100a4, Cd79a, Cd3e, and Epcam). Colors indicate gene expression density, with cool colors (blue) representing low-expression regions and warm colors (red) representing high-expression regions, further confirming the cell type specificity of these markers.

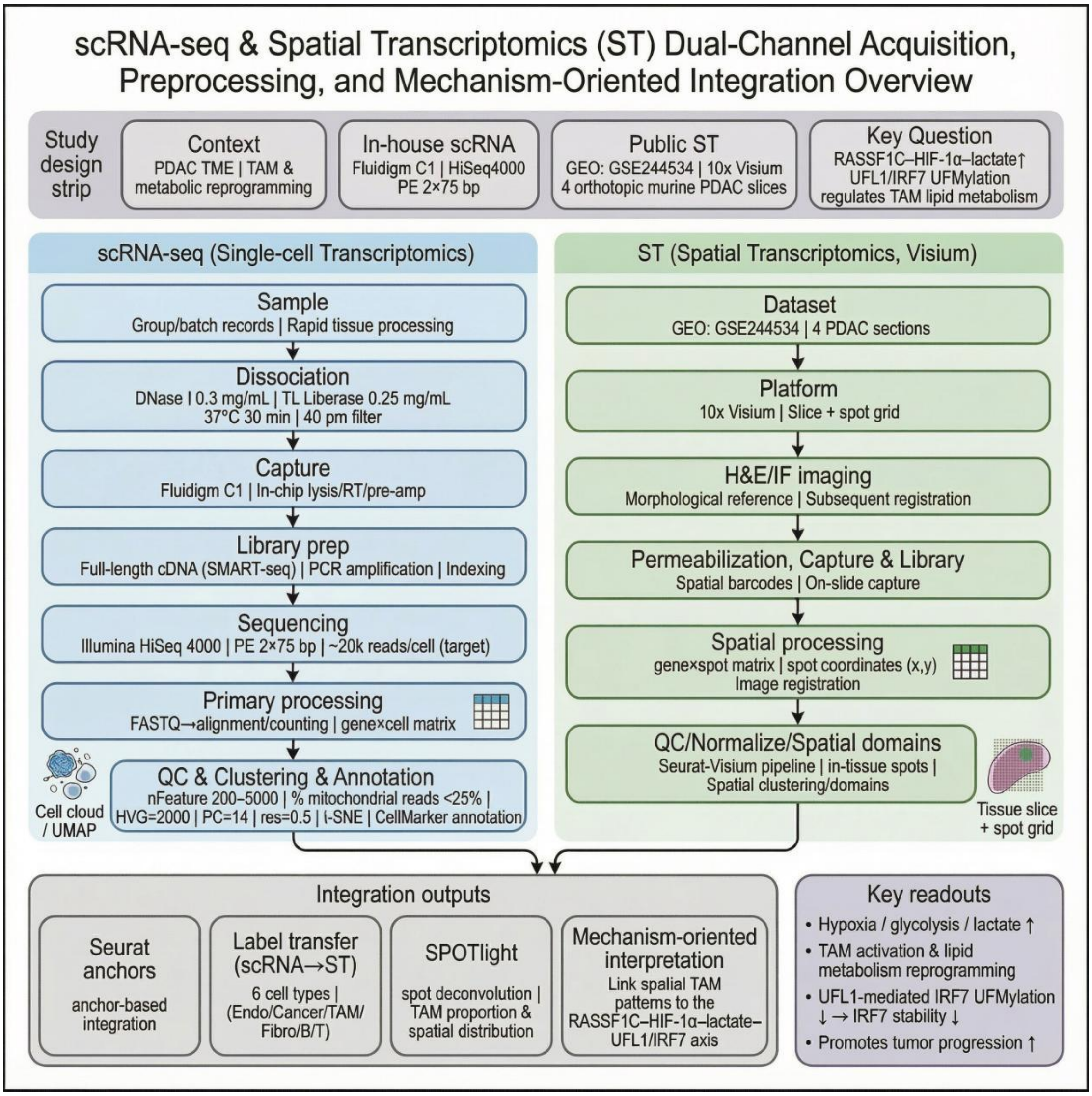


**Figure S4. Overview of the dual-modality data acquisition and preprocessing workflow for scRNA-seq and ST.**

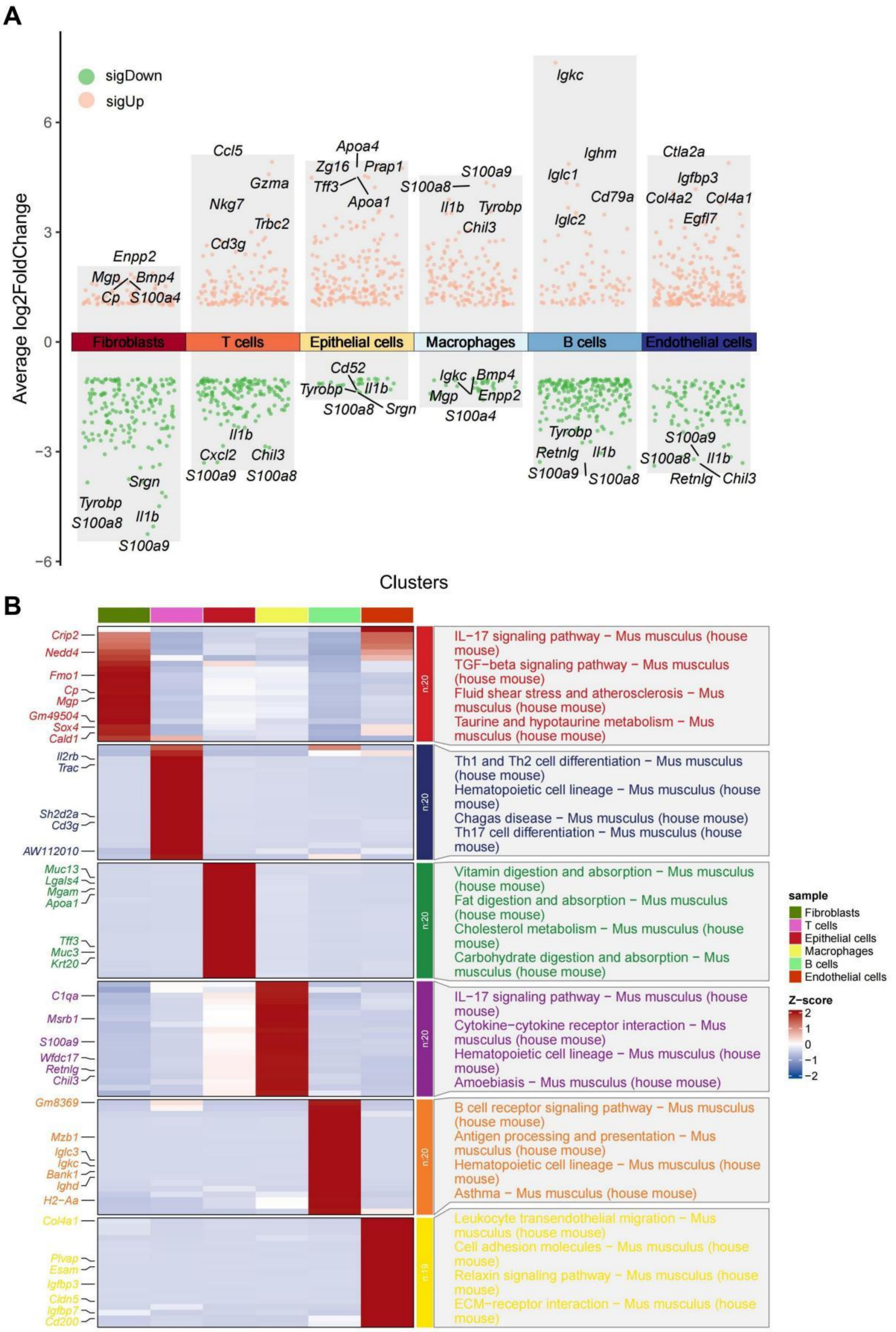


**Figure S5. scRNA-seq reveals cell type-specific gene expression and functional pathway analysis.**

Note: (A) Volcano plots of cell type-specific DEGs, showing genes that were

upregulated (red) or downregulated (green) across different cell populations, including T cells, epithelial cells, macrophages, and B cells. The x-axis represents the average $log_2FC$ in gene expression, and the y-axis indicates the level of statistical significance. (B) Heatmap of functional pathway enrichment based on DEGs. DEGs from each cell population were enriched in distinct KEGG pathways.

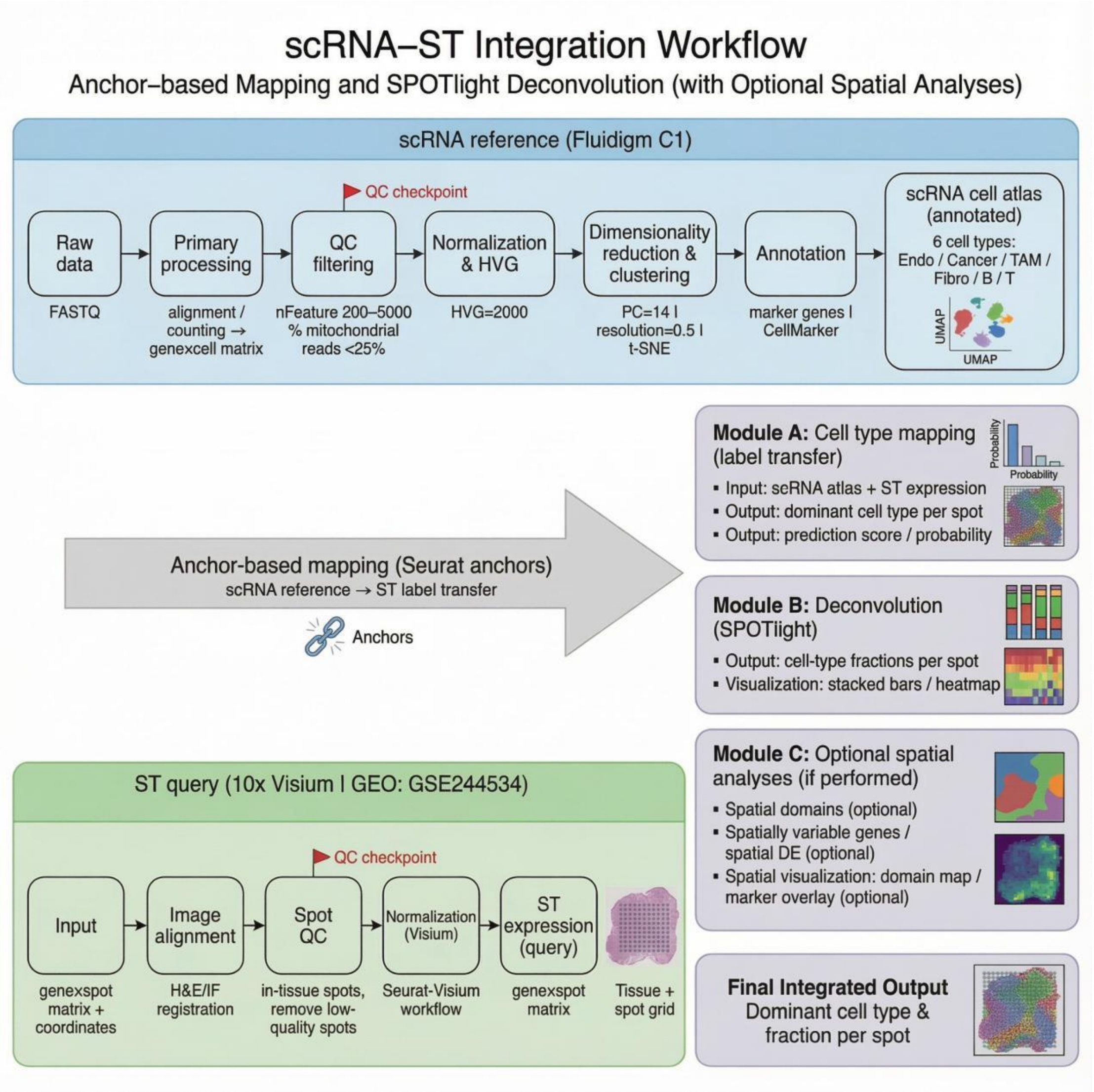


**Figure S6. Schematic overview of the scRNA-ST integrative analysis workflow: anchor-based mapping, label transfer, and SPOTlight deconvolution (including optional spatial analyses).**

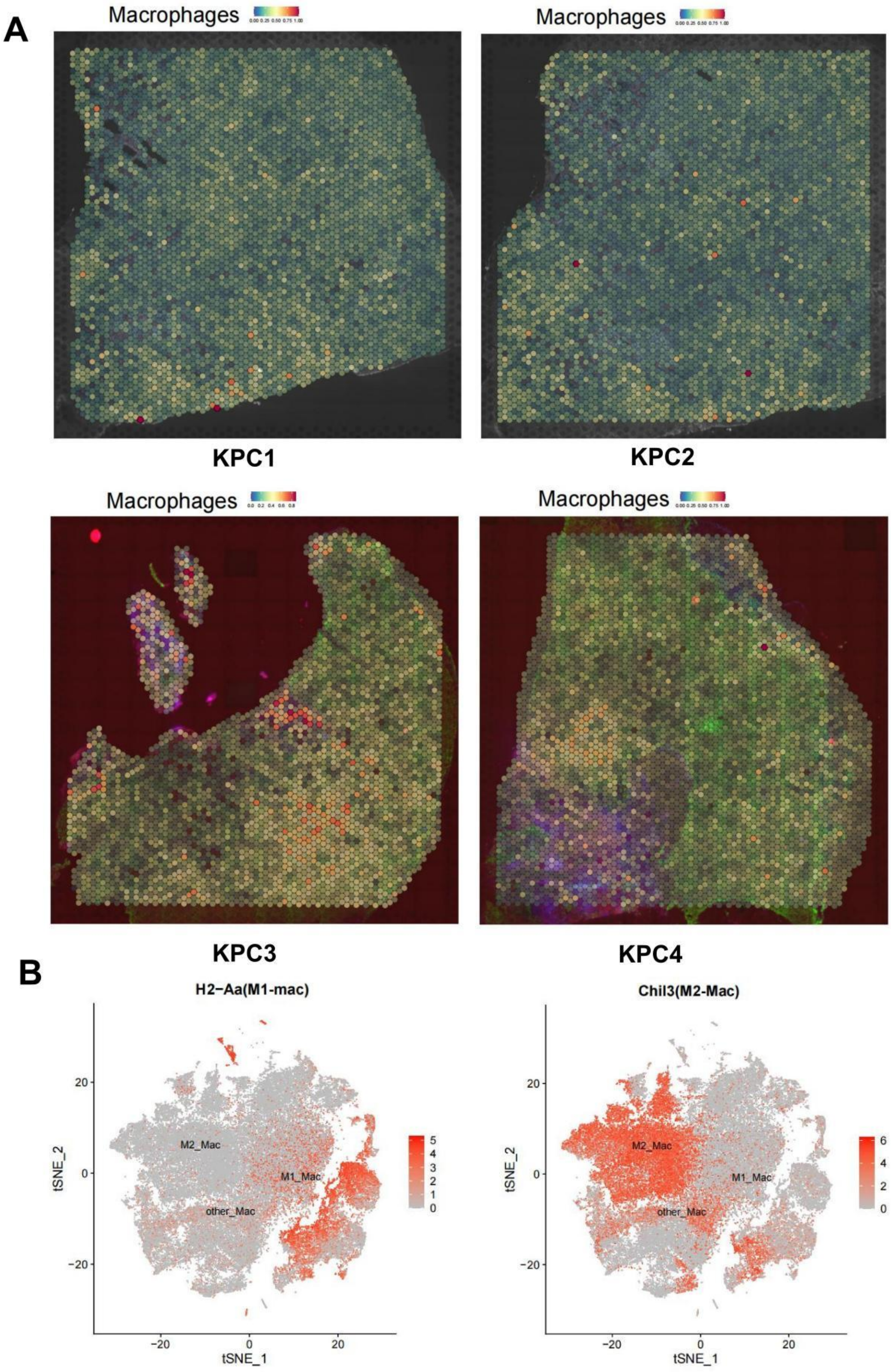


**Figure S7. Distribution of macrophages in KPC tissue samples.**

Note: (A) ST showing the spatial distribution of macrophages across four KPC model

samples (KPC1, KPC2, KPC3, and KPC4). Color intensity indicates the degree of macrophage enrichment, with the color scale ranging from blue to red to represent increasing enrichment. (B) t-SNE plot illustrating the expression distribution of M1 and M2 signature genes, with red indicating gene expression.

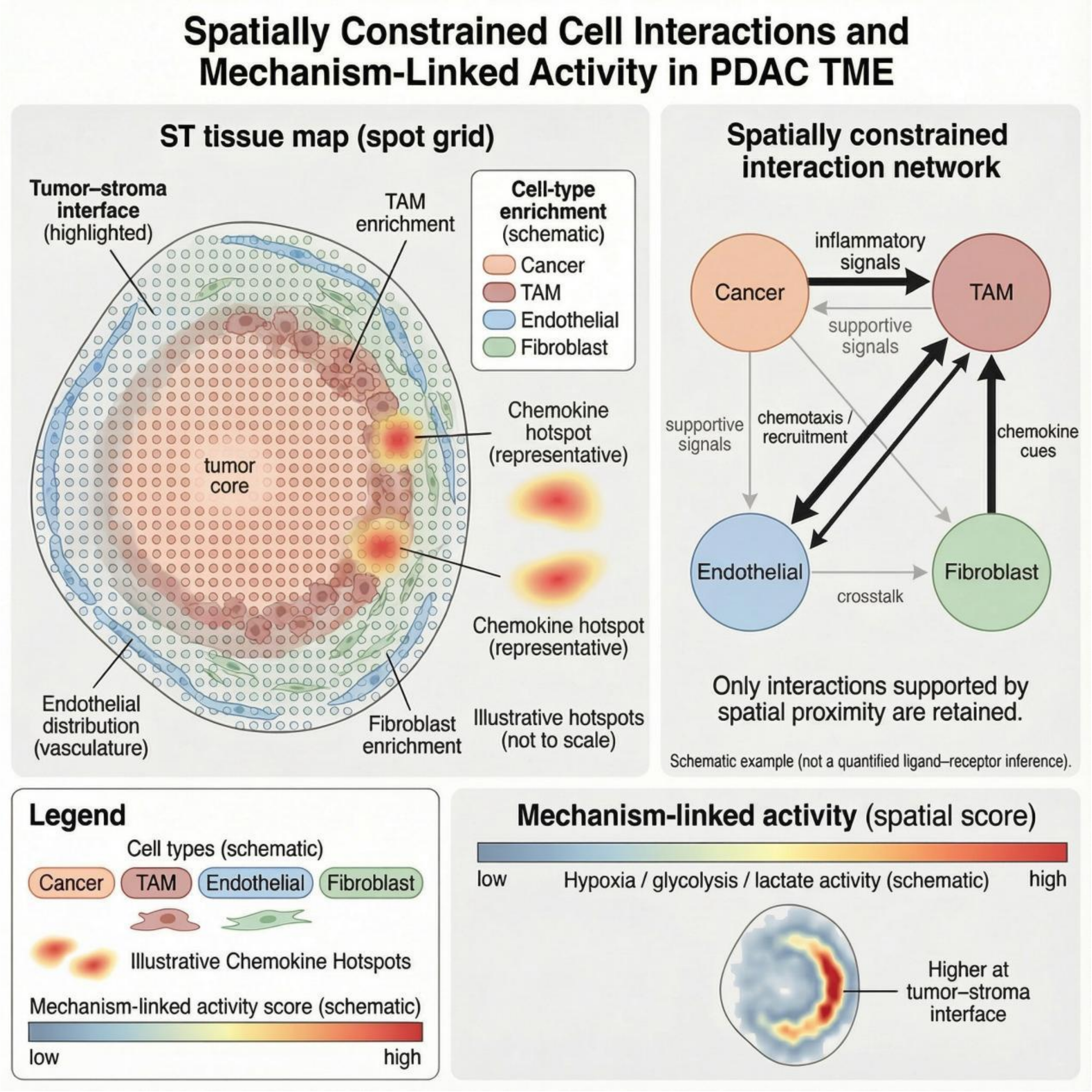


**Figure S8. Summary schematic of integrated ST analysis: spatially constrained cell-cell interactions and distribution of mechanism-related pathway activities in the PDAC microenvironment.**

Note: The left panel depicts the spatial enrichment and hotspot regions of major cell types inferred from scRNA-ST integration across tissue sections; the right panel illustrates cell-cell interaction networks under spatial proximity constraints; the

bottom panel shows spatial variation trends of mechanism-related pathway activity scores (e.g., hypoxia-, glycolysis-, and lactate-associated activities). This figure is intended for conceptual summary and result navigation rather than quantitative presentation.

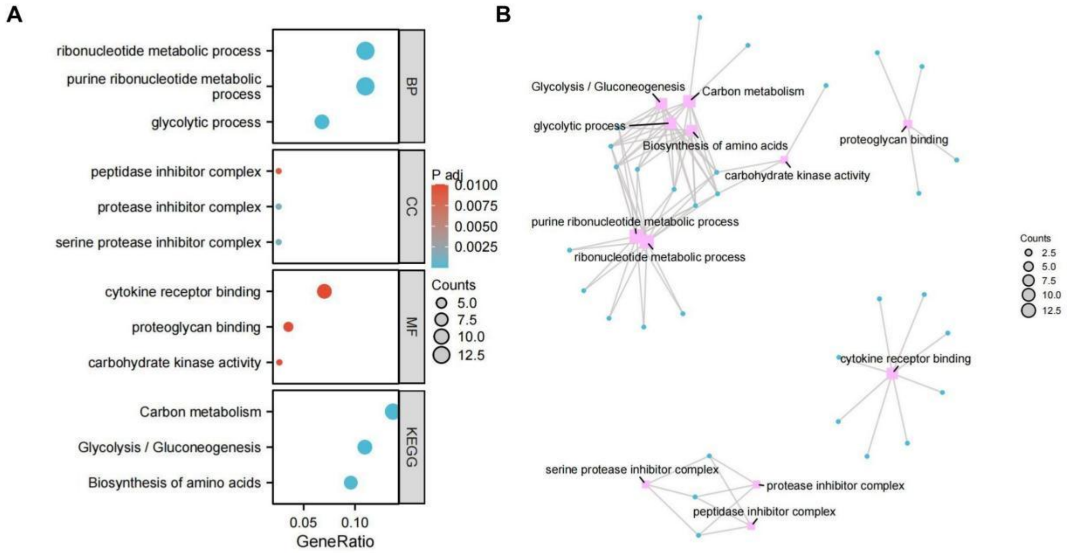


**Figure S9. Significant enrichment analysis of glycolysis-related pathways in PAAD cells under hypoxic conditions.**

Note: (A) GO and KEGG pathway analyses identifying significantly enriched biological processes (BP), cellular components (CC), molecular functions (MF), and metabolic pathways among DEGs under hypoxia; (B) Functional network construction of DEGs based on significantly enriched GO terms and KEGG pathways.

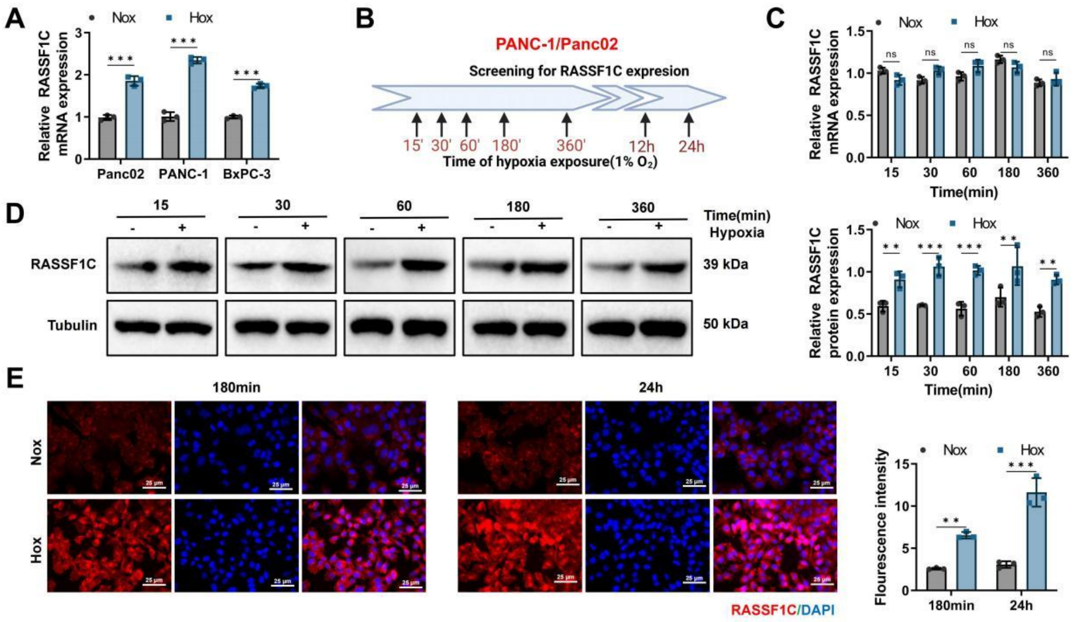


**Figure S10. Dynamic expression changes of RASSF1C under hypoxic conditions.**

Note: (A) Relative RASSF1C mRNA expression in Panc02, PANC-1, and BxPC-3 cells after normoxic (Nox) or hypoxic (Hox) treatment, as determined by qPCR; (B) Schematic illustration of the experimental design used in PANC-1 and Panc02 cells to define the hypoxia-induced time window for RASSF1C expression (acute hypoxia: 15-360 min; prolonged hypoxia: 12-24 h); (C) Dynamic analysis of relative RASSF1C mRNA expression in PANC-1 cells at different acute hypoxia exposure time points by qPCR; (D) Detection of RASSF1C protein levels in PANC-1 cells at different acute hypoxia exposure time points by Western blot (Tubulin as a loading control), with densitometric quantification; (E) Immunofluorescence staining of RASSF1C in PANC-1 cells after 180 min or 24 h of hypoxic treatment (RASSF1C, red; DAPI, blue), with quantitative analysis of fluorescence intensity (scale bar = 25 μm). All cell-based experiments were performed with three independent biological replicates, and data are presented as mean ± SD with individual data points overlaid. ns indicates no statistically significant difference; *$p < 0.05$, **$p < 0.01$, ***$p < 0.001$.

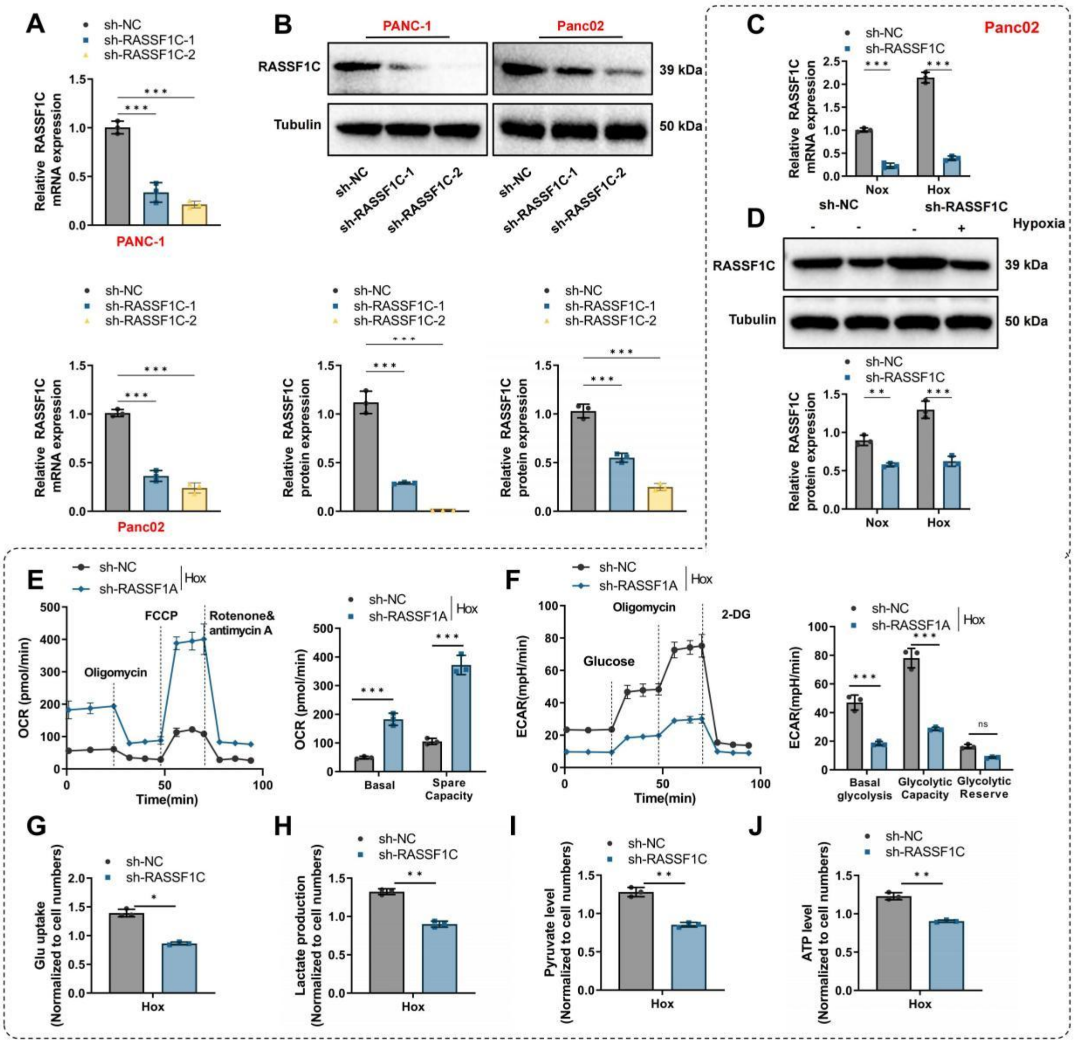


**Figure S11. Effects of RASSF1C knockdown on glycolysis and metabolic properties of PAAD cells.**

Note: (A) Changes in RASSF1C mRNA levels in PANC-1 and Panc02 cells after RASSF1C knockdown; (B) Changes in RASSF1C protein levels in PANC-1 and Panc02 cells after RASSF1C knockdown; (C) Alterations in mRNA and protein levels in Panc02 cells following RASSF1C knockdown under normoxic (Nox) and hypoxic (Hox) conditions; (D) Changes in RASSF1C protein expression in Panc02 cells after RASSF1C knockdown under Nox and Hox conditions; (E) OCR in cells after RASSF1C knockdown, assessed by sequential treatment with the mitochondrial ATP synthase inhibitor Oligo, the uncoupler FCCP, and the complex I inhibitor rotenone,

to evaluate basal and maximal respiratory capacity; (F) ECAR after RASSF1C knockdown, reflecting glycolytic activity; (G) Changes in glucose uptake following RASSF1C knockdown; (H) Changes in lactate production following RASSF1C knockdown; (I) Changes in pyruvate levels following RASSF1C knockdown; (J) Changes in ATP levels following RASSF1C knockdown. All experiments were performed with three independent biological replicates. Data are presented as mean ± SD with individual data points overlaid. ns indicates no statistically significant difference; **p* < 0.05, ***p* < 0.01, ****p* < 0.001.

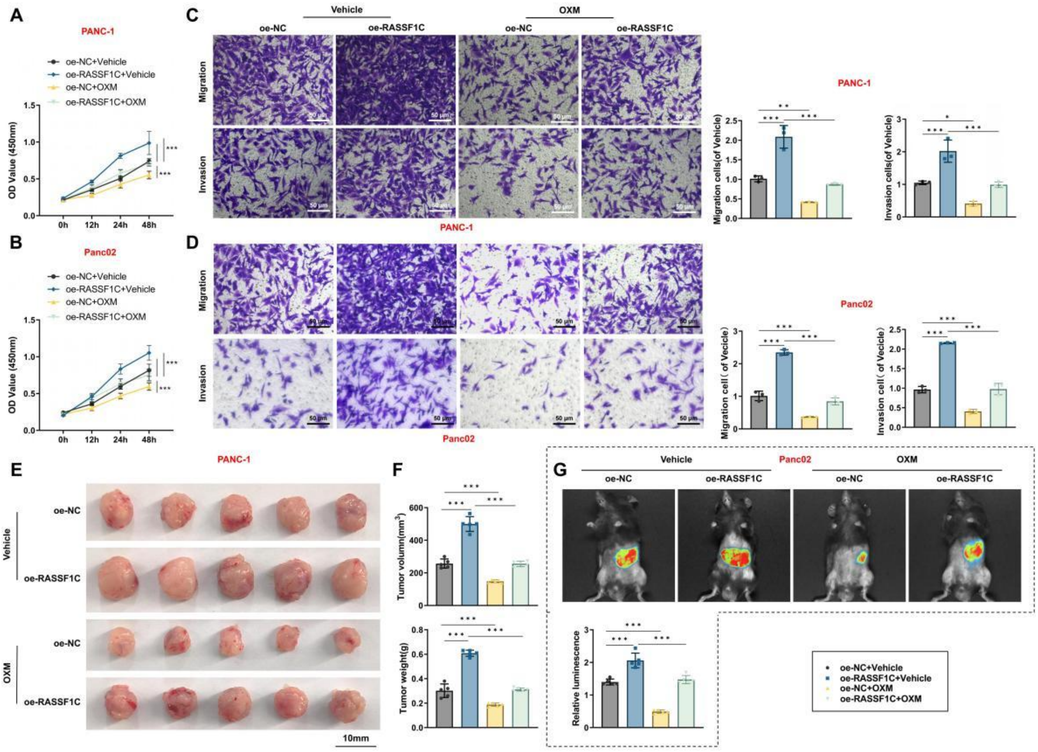


**Figure S12. Effects of RASSF1C on PAAD cell migration, invasion, and *in vivo* tumor growth.**

Note: (A-B) CCK-8 assays assessing cell viability of PANC-1 (A) and Panc02 (B) cells under RASSF1C overexpression (oe-RASSF1C) or control (oe-NC) conditions, followed by treatment with OXM or vehicle control (Vehicle) (time points are indicated in the figure); (C-D) Transwell assays evaluating the migratory and invasive

capacities of PANC-1 (C) and Panc02 (D) cells under the same treatment conditions: migration assays were performed without Matrigel, whereas invasion assays were conducted with Matrigel coating; cells were stained with crystal violet, counted, and quantified (scale bar = 50 μm); (E-F) Subcutaneous xenograft model using PANC-1 cells: representative images of tumors (E, scale bar = 10 mm) and statistical analysis of tumor volume and weight at the experimental endpoint (F), with groups receiving oe-NC or oe-RASSF1C combined with vehicle or OXM treatment; (G) Representative bioluminescence imaging (BLI) images and signal quantification of the Panc02 orthotopic xenograft model to assess tumor burden across different treatment groups (grouping as described above). For animal experiments, n = 5 per group. Data are presented as mean ± SD with individual data points overlaid. ns indicates no statistically significant difference; *$p < 0.05$, **$p < 0.01$, ***$p < 0.001$.

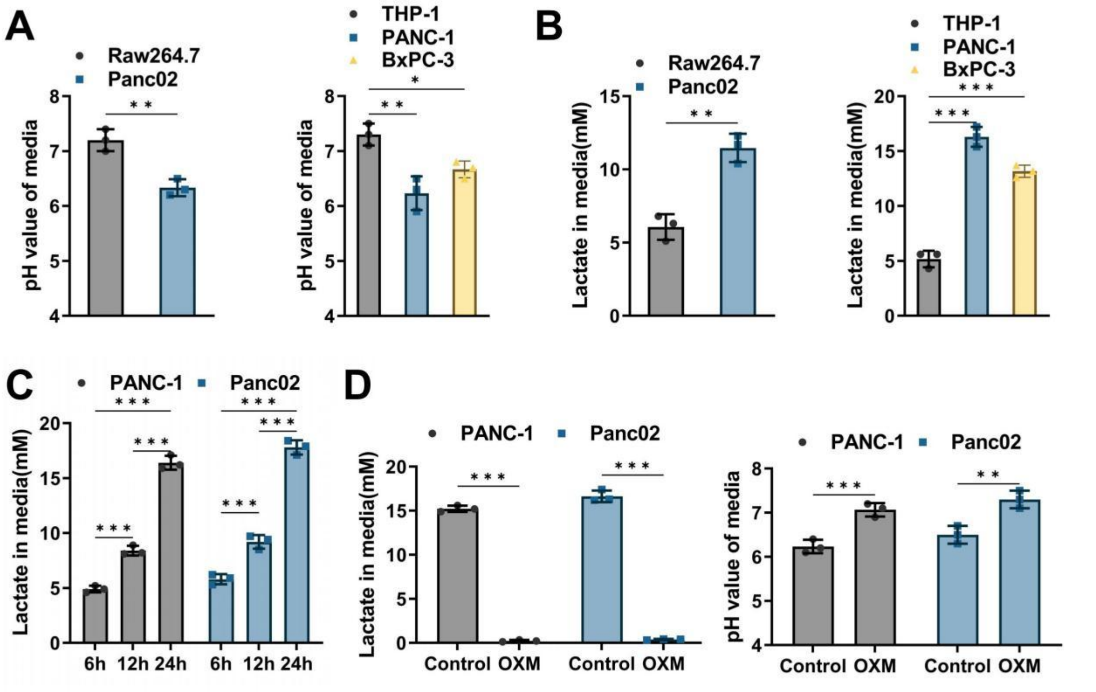


**Figure S13. Lactate production and pH changes in PAAD cells under hypoxic conditions.**

Note: (A) Measurement of pH in CM from RAW264.7 and Panc02 cells, as well as

pH assessment of THP-1 CM after supplementation with CM derived from different PAAD cell lines (PANC-1 and BxPC-3) (grouping as indicated in the figure legend); (B) Measurement of lactate concentrations in CM from RAW264.7 and Panc02 cells, and in THP-1 CM following supplementation with CM from PANC-1 or BxPC-3 cells (grouping as indicated in the figure legend); (C) Lactate concentrations in CM from PANC-1 and Panc02 cells measured at 6 h, 12 h, and 24 h; (D) Measurement of lactate concentration and pH in CM from PANC-1 and Panc02 cells treated with OXM or control conditions. All cell-based experiments were performed with three independent biological replicates. Data are presented as mean ± SD with individual data points overlaid. ns indicates no statistically significant difference; *$p < 0.05$, **$p < 0.01$, ***$p < 0.001$.

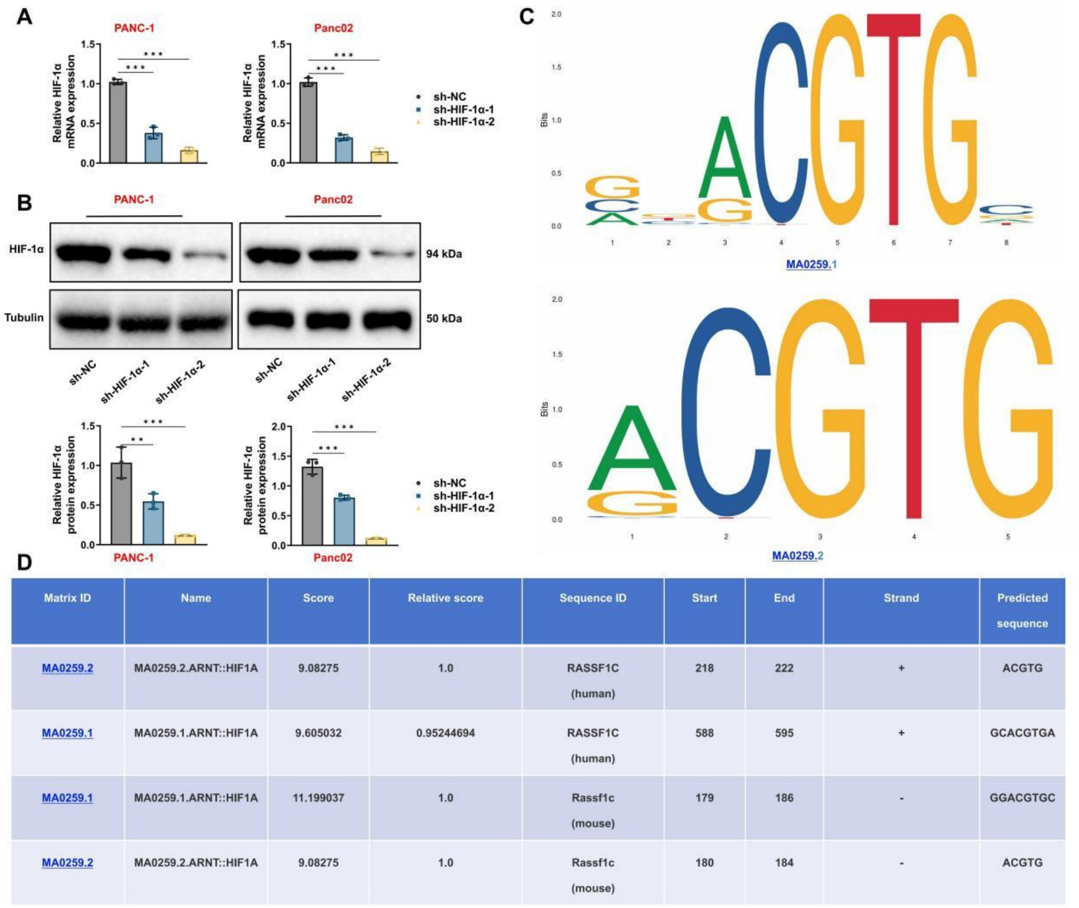


| Matrix ID | Name | Score | Relative score | Sequence ID | Start | End | Strand | Predicted sequence |
|---|---|---|---|---|---|---|---|---|
| MA0259.2 | MA0259.2.ARNT::HIF1A | 9.08275 | 1.0 | RASSF1C (human) | 218 | 222 | + | ACGTG |
| MA0259.1 | MA0259.1.ARNT::HIF1A | 9.605032 | 0.95244694 | RASSF1C (human) | 588 | 595 | + | GCACGTGA |
| MA0259.1 | MA0259.1.ARNT::HIF1A | 11.199037 | 1.0 | Rassf1c (mouse) | 179 | 186 | - | GGACGTGC |
| MA0259.2 | MA0259.2.ARNT::HIF1A | 9.08275 | 1.0 | Rassf1c (mouse) | 180 | 184 | - | ACGTG |

**Figure S14. Transcriptional regulation of RASSF1C expression by HIF-1α.**

Note: (A) Relative HIF-1α mRNA expression in PANC-1 and Panc02 cells after transfection with sh-HIF-1α-1, sh-HIF-1α-2, or control sh-NC, as determined by RT-qPCR; (B) HIF-1α protein levels under the same conditions assessed by Western blot (Tubulin as a loading control), with densitometric quantification; (C) Prediction of potential HIF-1α/ARNT binding motifs within the RASSF1C promoter region using the JASPAR database, with sequence logos shown; (D) Summary of predicted candidate binding sites, including matrix ID, genomic coordinates, strand orientation, and predicted sequences with corresponding scores (human and mouse sequences are indicated separately). All cell-based experiments were performed with three independent biological replicates. Data are presented as mean ± SD with individual data points overlaid. ns indicates no statistically significant difference; $^{*}p < 0.05$, $^{**}p < 0.01$, $^{***}p < 0.001$.

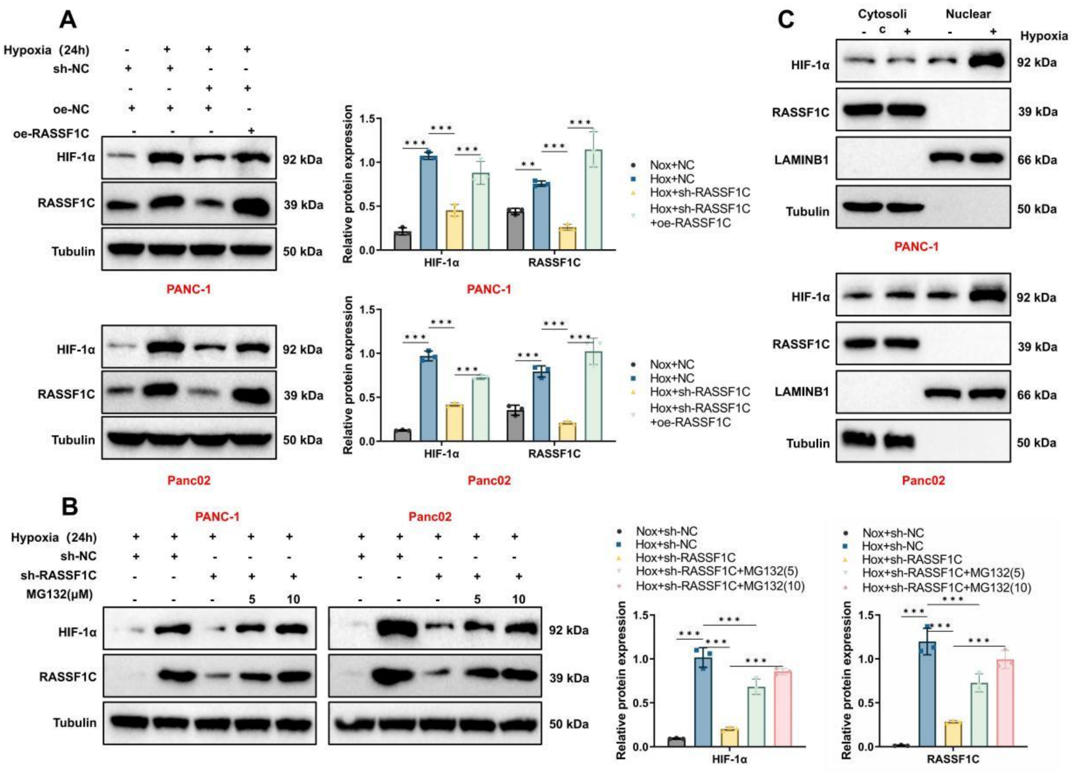


**Figure S15. RASSF1C stabilizes HIF-1α protein by suppressing HIF-1α hydroxylation and ubiquitination.**

Note: (A) HIF-1α and RASSF1C protein levels in PANC-1 and Panc02 cells subjected to sh-RASSF1C or oe-RASSF1C under normoxic (Nox) or hypoxic (Hox, 24 h) conditions, as assessed by Western blot (Tubulin as a loading control), with densitometric quantification; (B) HIF-1α and RASSF1C protein levels in PANC-1 and Panc02 cells treated under hypoxic conditions (24 h) in combination with the proteasome inhibitor MG132 (5 or 10 μM), analyzed by Western blot (Tubulin as a loading control), with densitometric quantification; (C) Subcellular fractionation followed by Western blot analysis to determine the distribution of HIF-1α and RASSF1C in cytoplasmic and nuclear fractions (LAMINB1 as a nuclear loading control and Tubulin as a cytoplasmic loading control). All cell-based experiments were performed with three independent biological replicates. Data are presented as mean ± SD with individual data points overlaid. ns indicates no statistically significant difference; $*p < 0.05$, $**p < 0.01$, $***p < 0.001$.

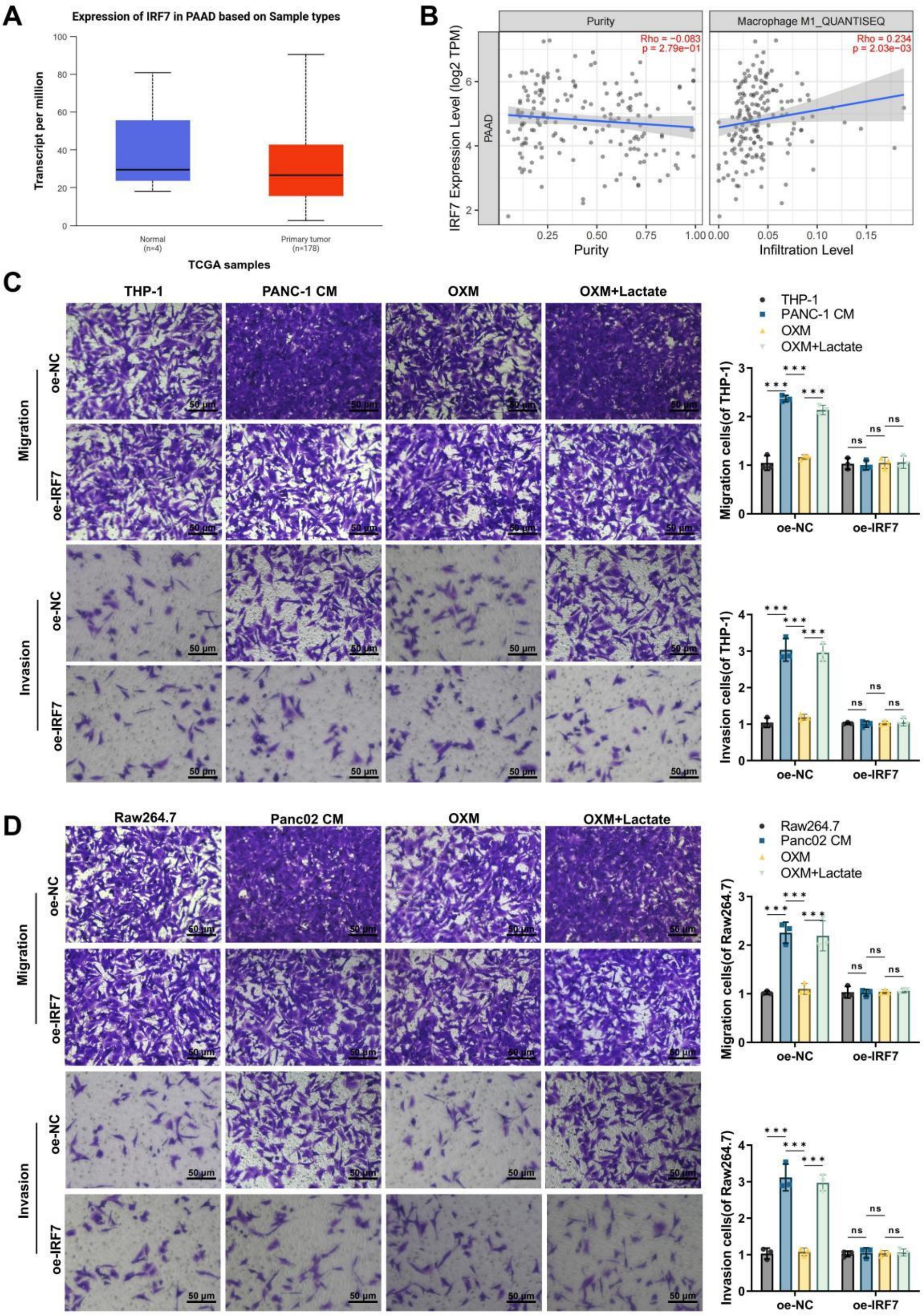


**Figure S16. Relationship between IRF7 expression levels, lactate-induced macrophage polarization, and PAAD cell invasion.**

Note: (A) Analysis of IRF7 expression levels in PAAD based on public databases; (B) Correlation analysis between IRF7 expression and the degree of M1 macrophage infiltration; (C) Effects of CM from THP-1-derived macrophages under different

conditions on the migration and invasion of PANC-1 cells (scale bar = 50 μm); (D) Effects of CM from RAW264.7 macrophages under different conditions on the migration and invasion of Panc02 cells (scale bar = 50 μm). All cell-based experiments were performed with three independent biological replicates. Data are presented as mean ± SD with individual data points overlaid. ns indicates no statistically significant difference; $**p < 0.01$, $***p < 0.001$.

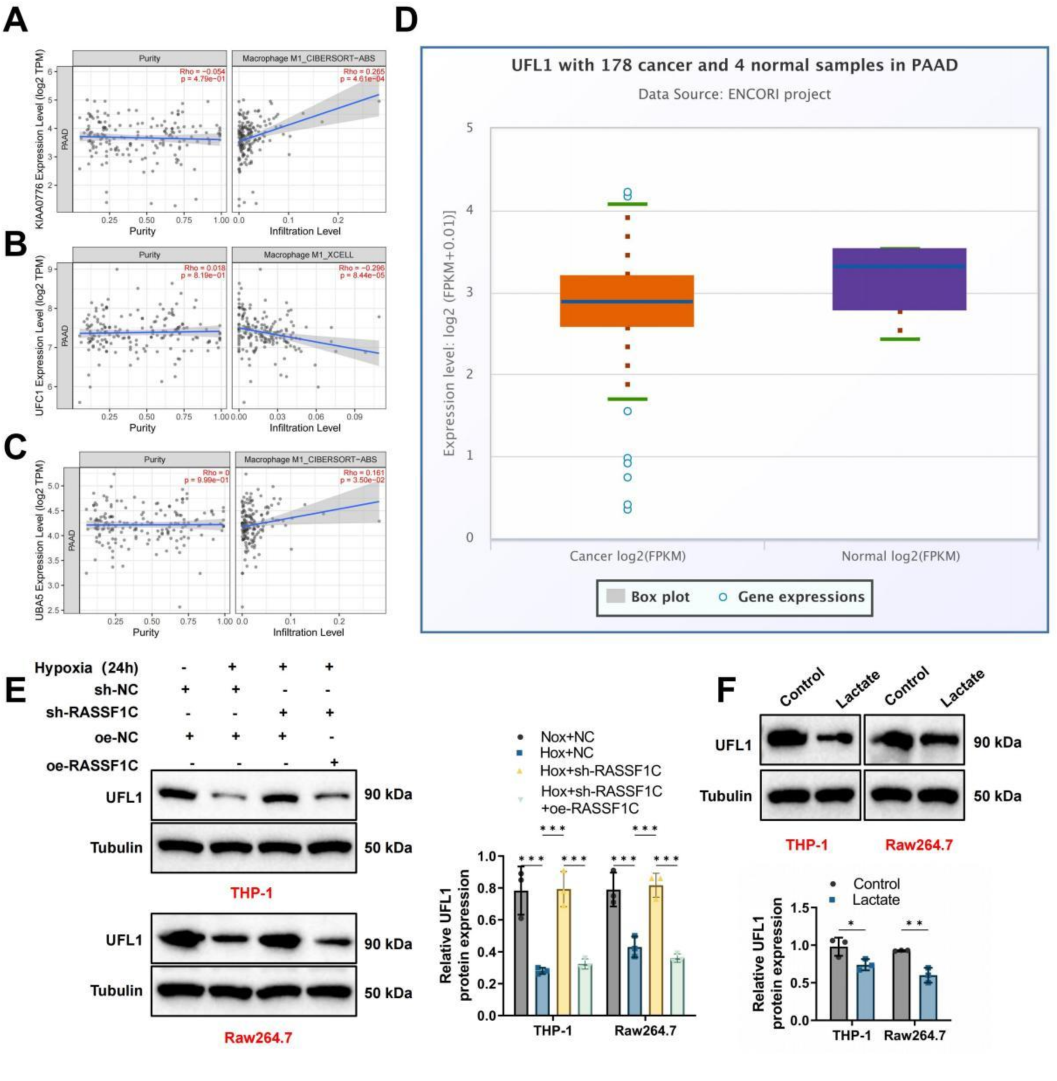


**Figure S17. Regulation of UFMylation-related molecules and their association with M1 macrophage infiltration in the PAAD microenvironment.**

Note: (A-C) TIMER v2.0 database analyses showing that UFL1 (A) and UBA5 (B) were positively correlated with M1 macrophage infiltration levels, whereas UFC1 (C)

was negatively correlated with M1 macrophage infiltration; (D) StarBase database analysis showing low UFL1 expression in PAAD; (E) UFL1 protein expression in THP-1 and RAW264.7 macrophages under hypoxic and RASSF1C-modulated conditions; (F) Effects of exogenous lactate treatment on UFL1 protein expression in THP-1 and RAW264.7 macrophages. All cell-based experiments were performed with three independent biological replicates. Data are presented as mean ± SD with individual data points overlaid; ***$p < 0.001$.

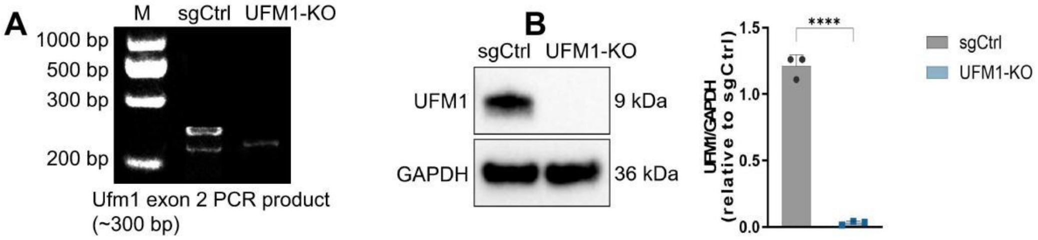


**Figure S18. Validation of Ufm1 knockout in RAW264.7 cells.**

Note: (A) Genomic DNA PCR amplification targeting exon 2 of Ufm1 followed by agarose gel electrophoresis (approximately 300 bp product; M, DNA marker; sgCtrl, control; UFM1-KO, knockout clones). (B) Western blot analysis of UFM1 protein levels in sgCtrl and UFM1-KO cells (GAPDH as a loading control), with densitometric quantification (UFM1/GAPDH, normalized to sgCtrl). Experiments were performed with three independent biological replicates. Data are presented as mean ± SD with individual data points overlaid. Comparisons between two groups were performed using an unpaired two-tailed t test; ***$p < 0.001$.
