# Supplementary material for: The RASSF1C-HIF-1α axis drives macrophage lipid metabolism to promote pancreatic cancer
Source: Cell Death Dis. 2026 Mar 30;17(1):430. doi: 10.1038/s41419-026-08609-0 (PMC13156295; doi:10.1038/s41419-026-08609-0)

Figure 2

F1-F4

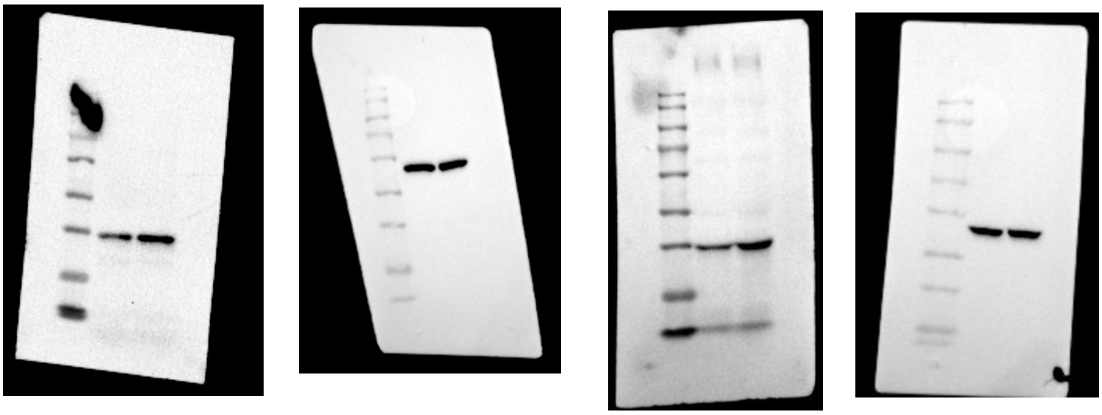

I1-I2

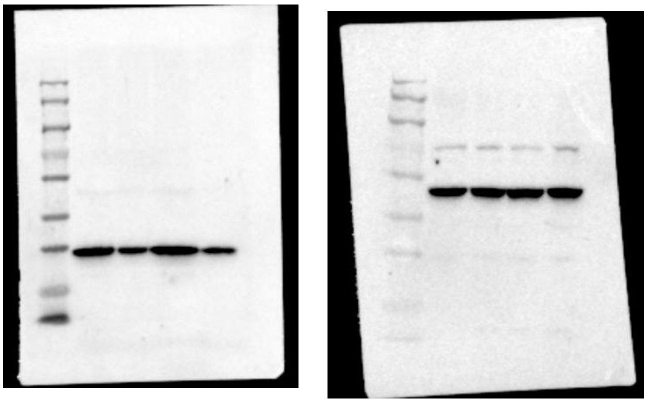

Figure 4

A1-A6

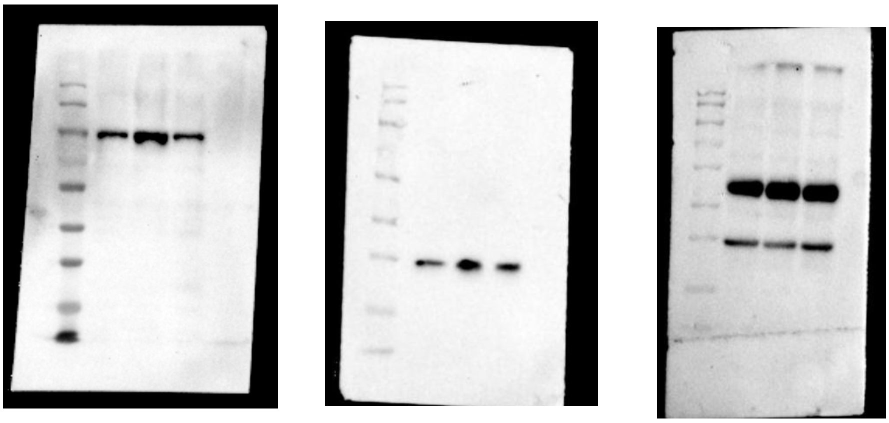

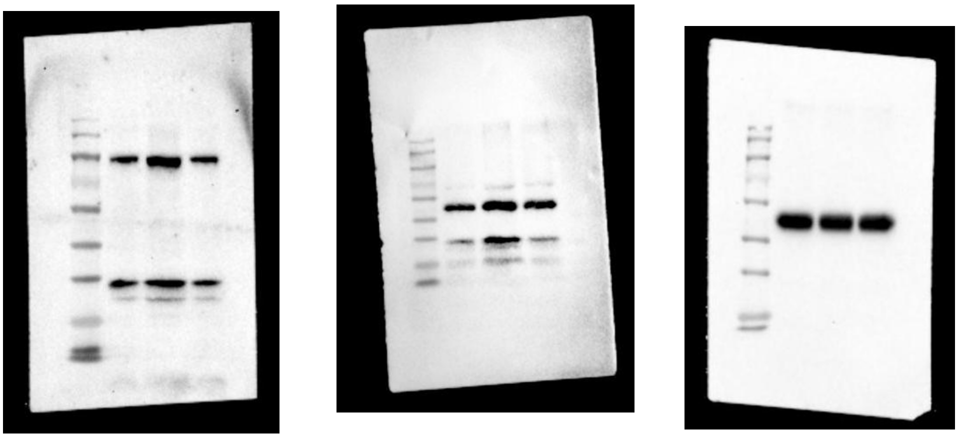

C1-C4

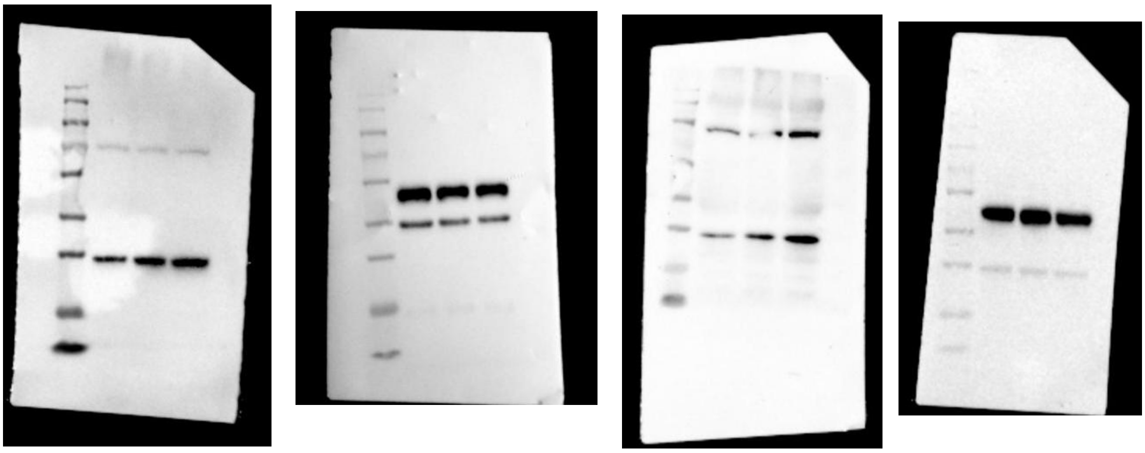

Figure 5

A1-A6

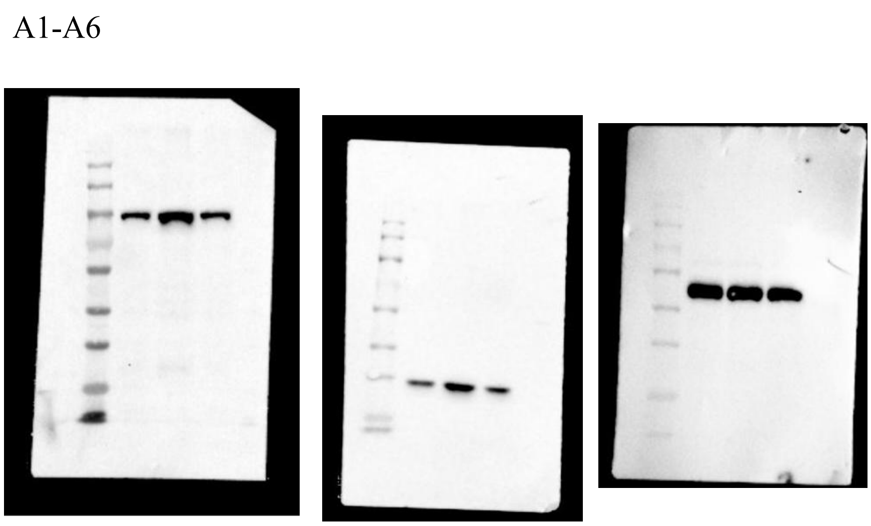

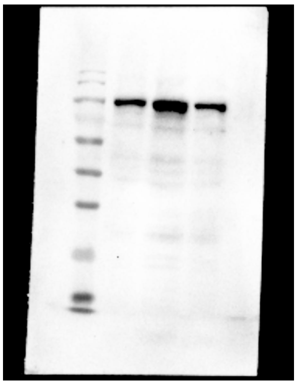
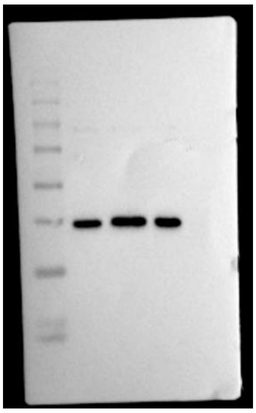
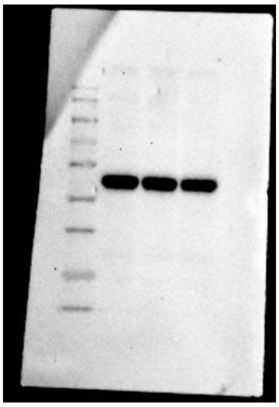

B1-B6

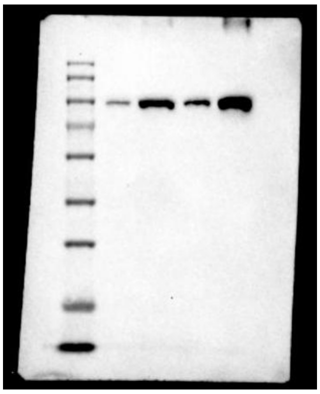
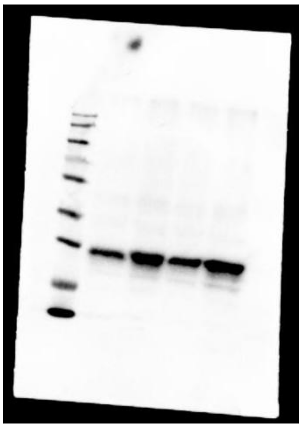
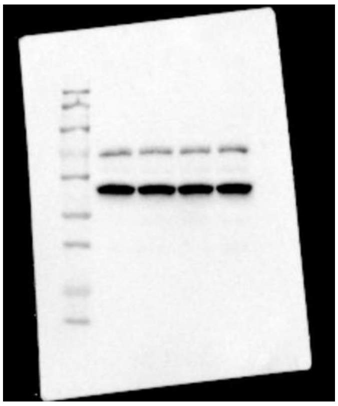
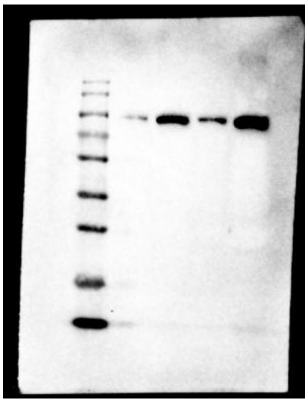
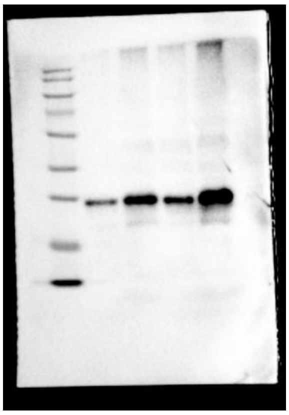
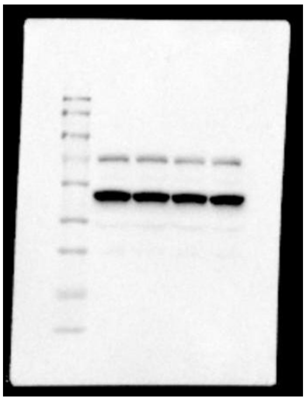

E1-E8

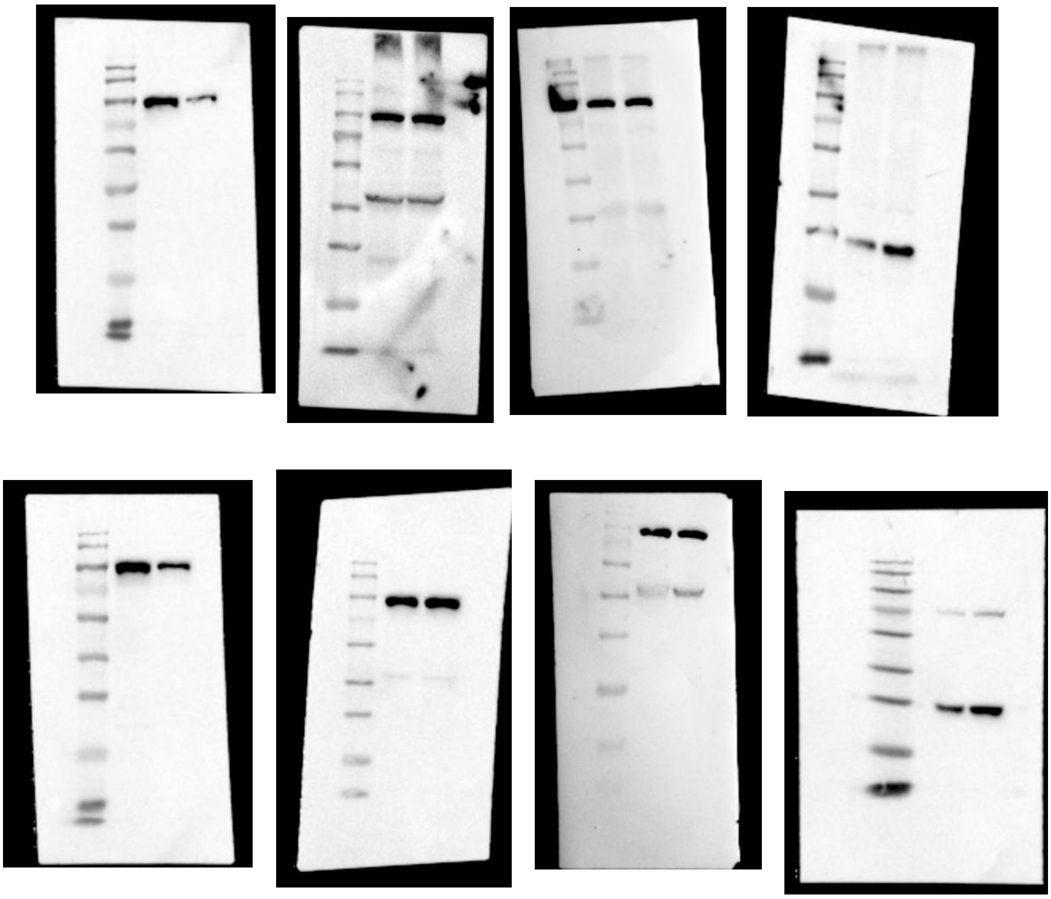

F1-F8

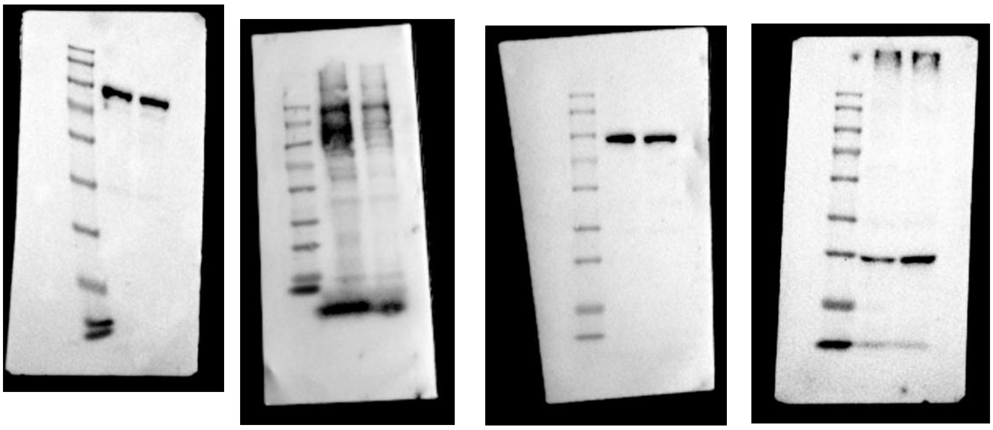

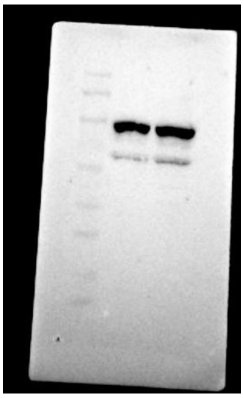
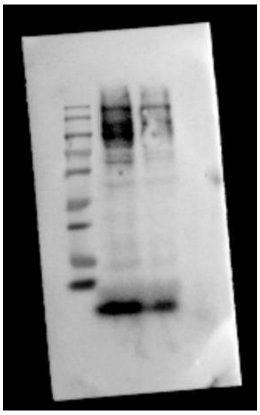
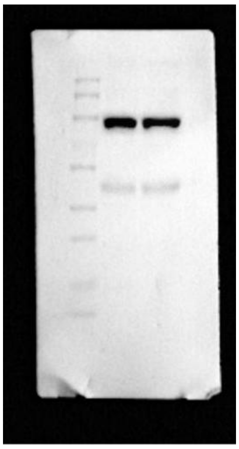
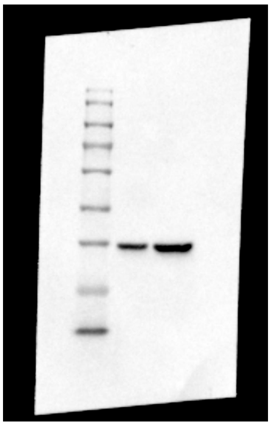

G1-1 -G1-8

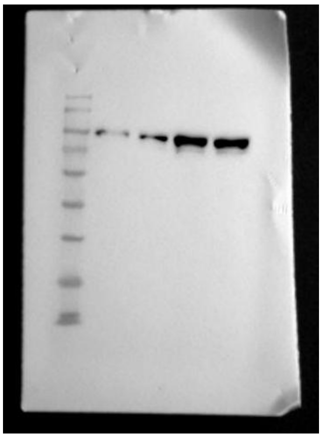
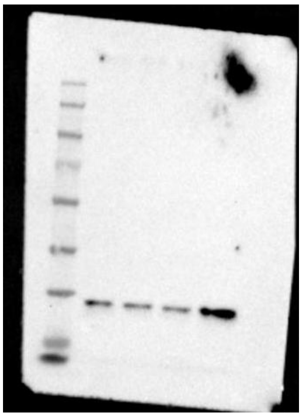
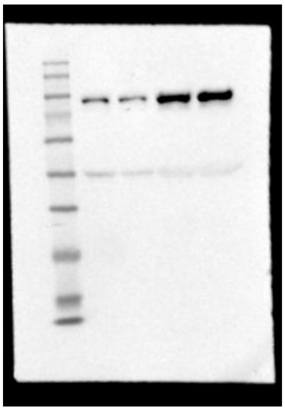
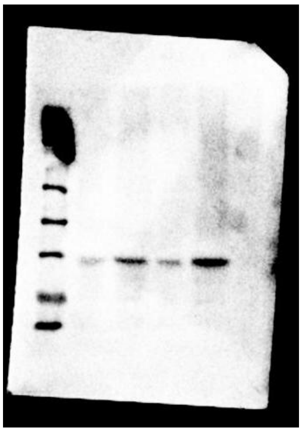
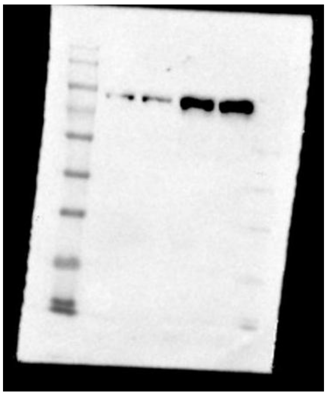
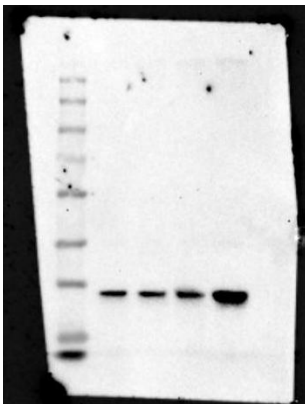
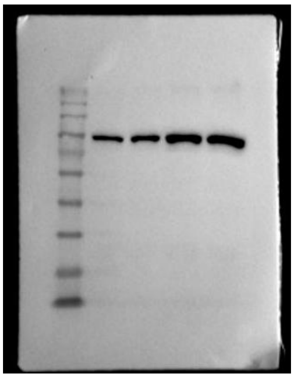
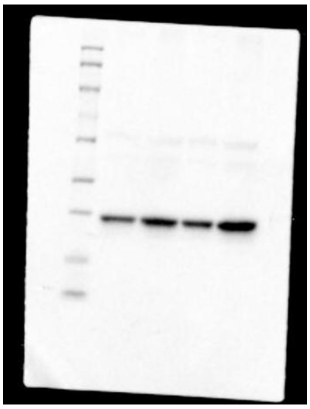

G2-1 -G2-8

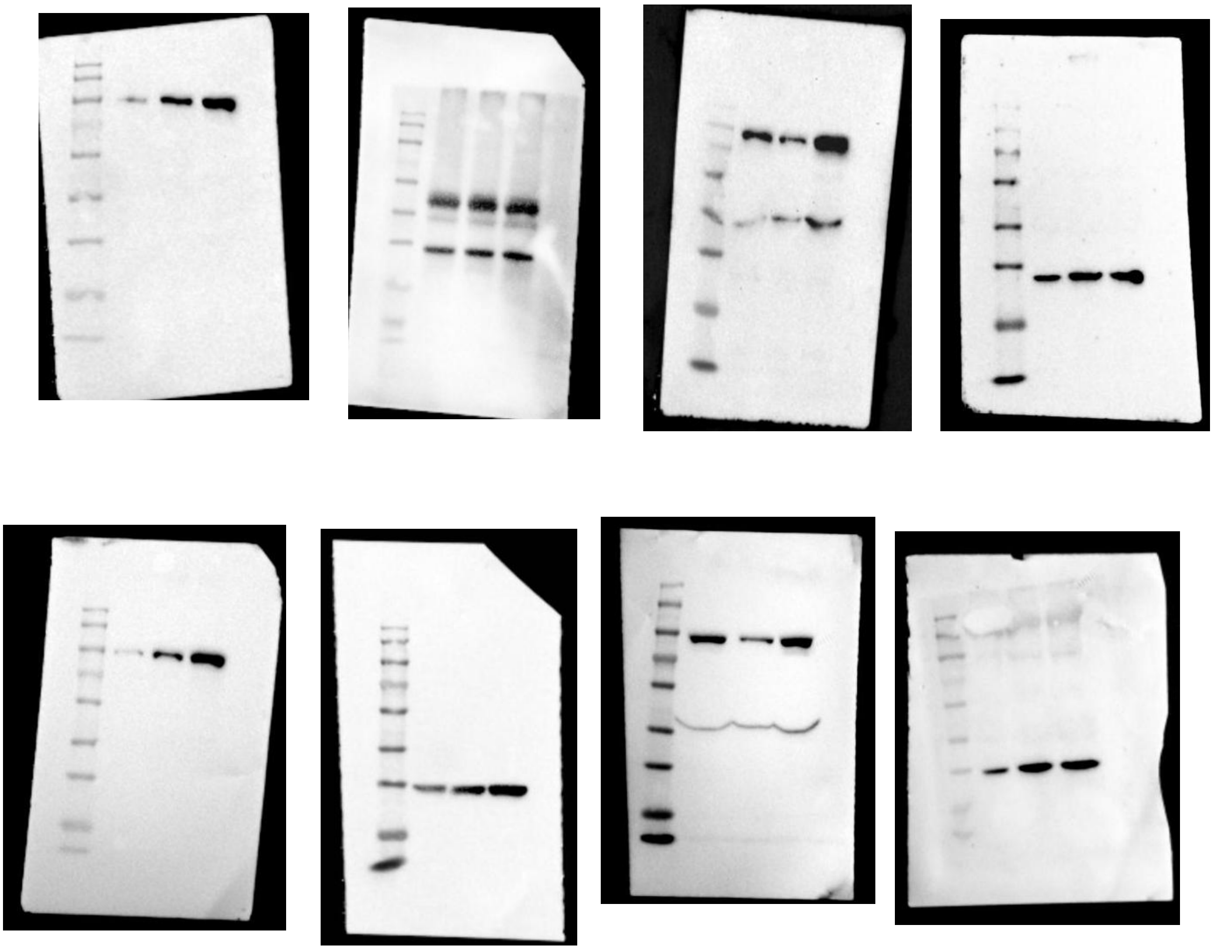

5H1-H4

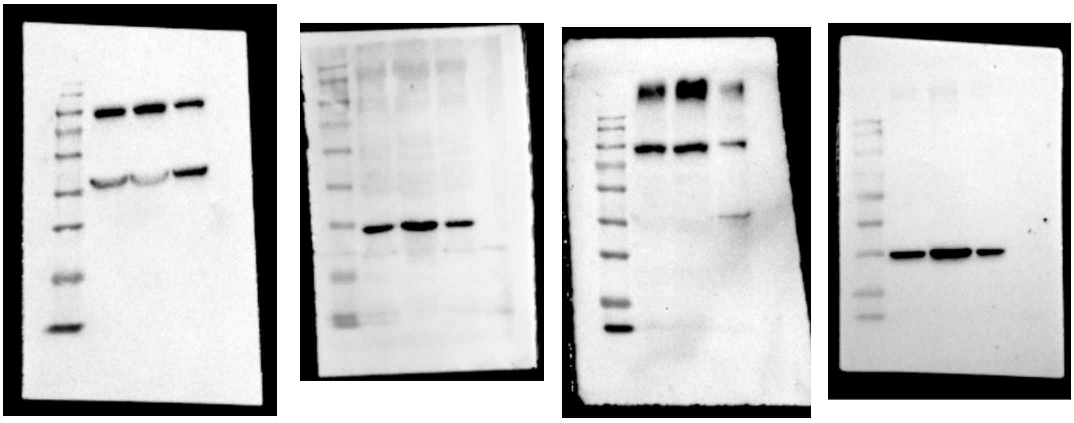

Figure 6

A1-A4

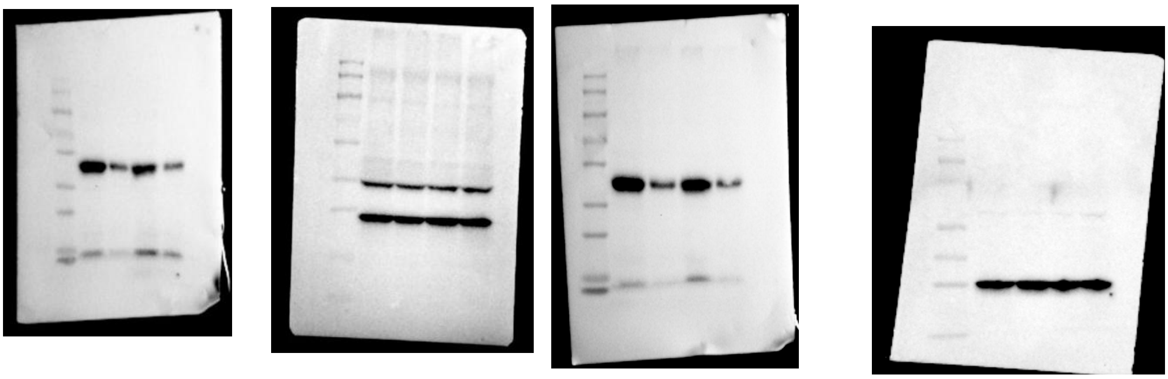

B1 -B4

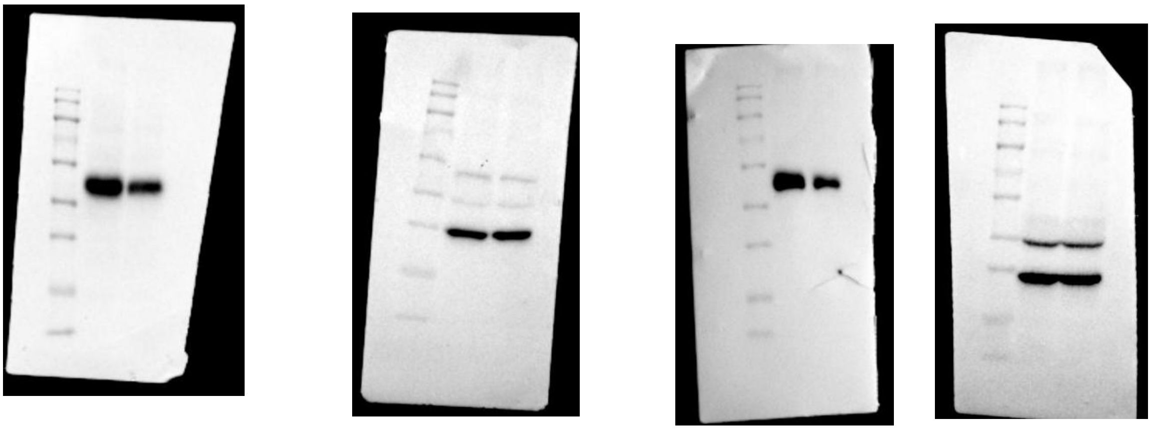

Figure 8

A1-A4

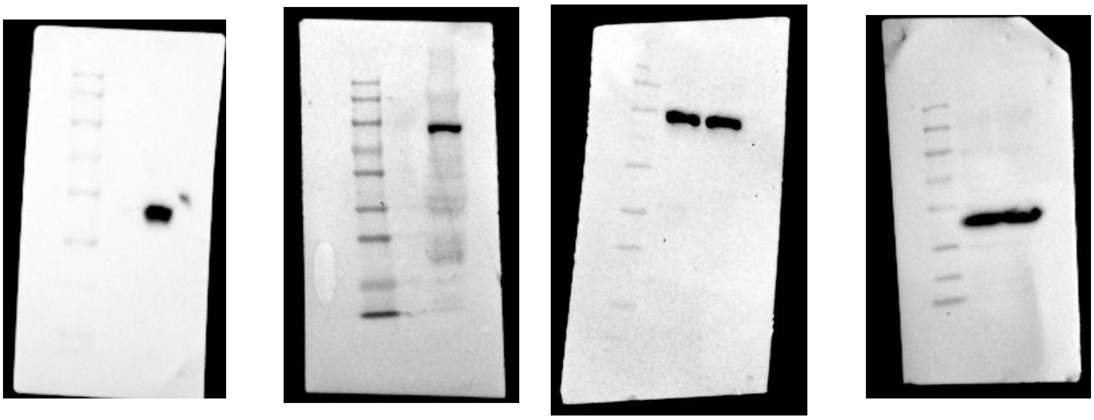

B1-B3

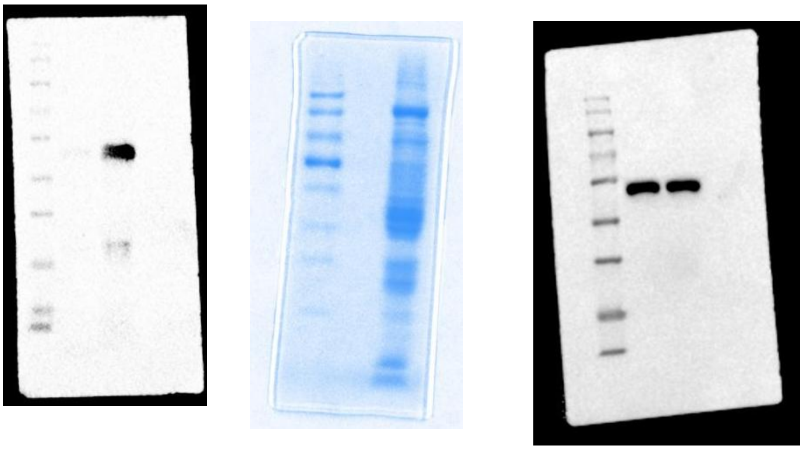

C1-C3

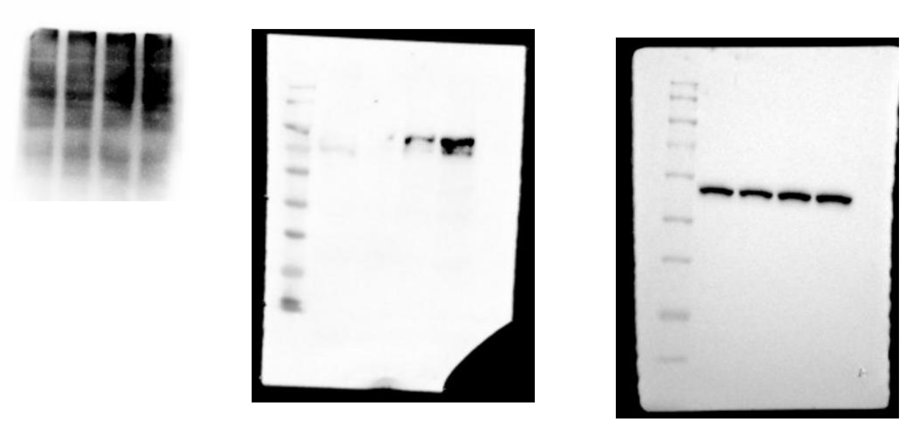

D1-D3

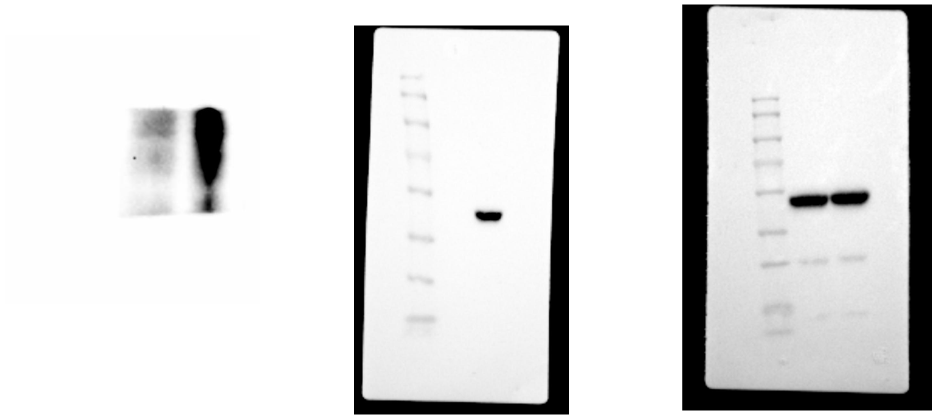

E1-E4

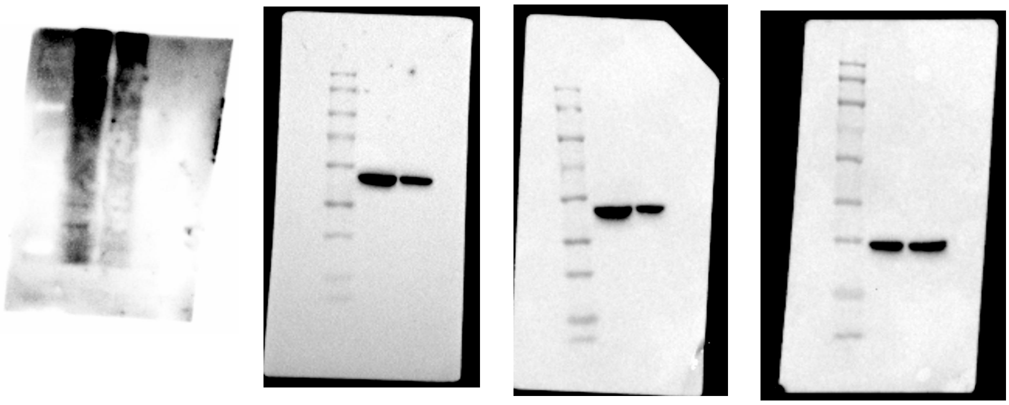

F1-F3

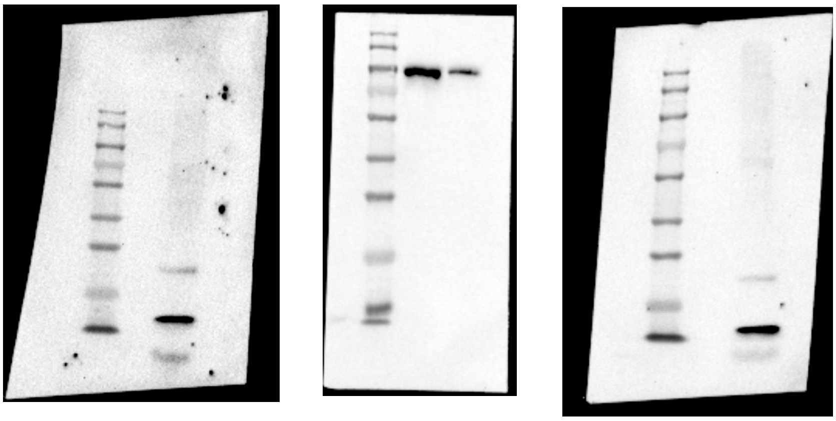

G1-G3

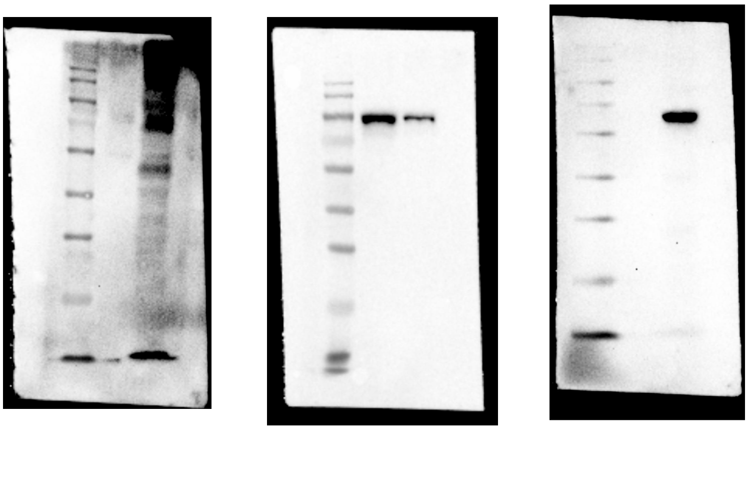

H1-H3

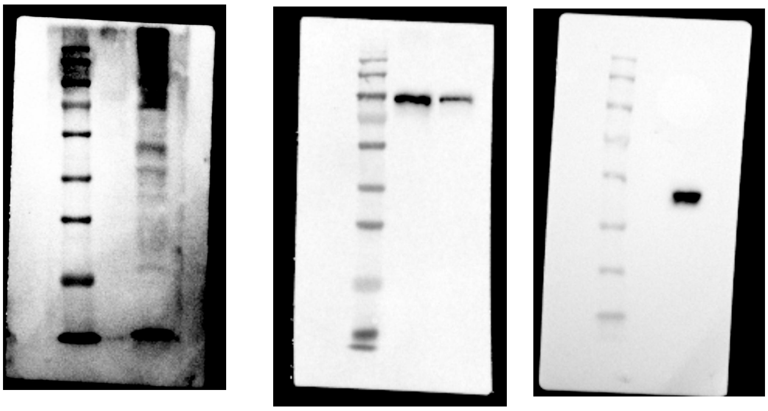

I1-I5

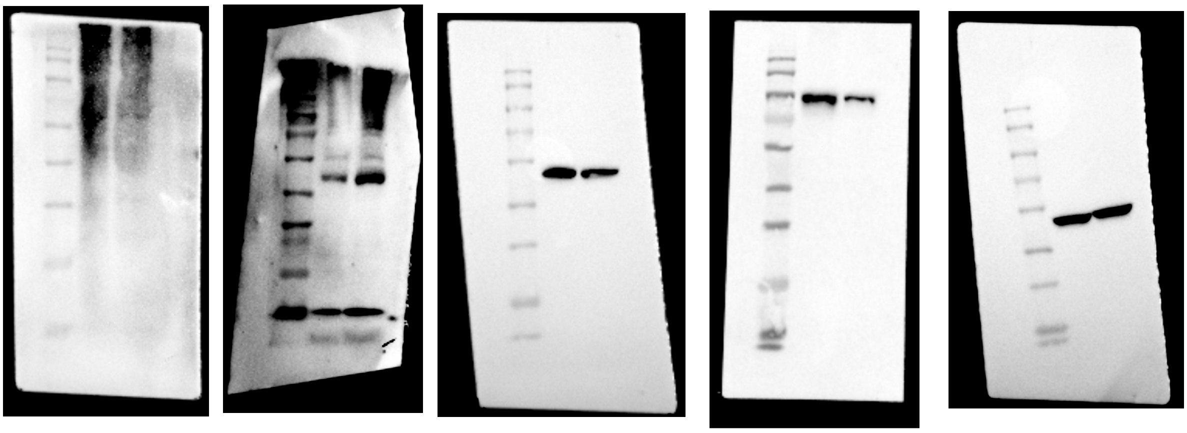

J1-J4

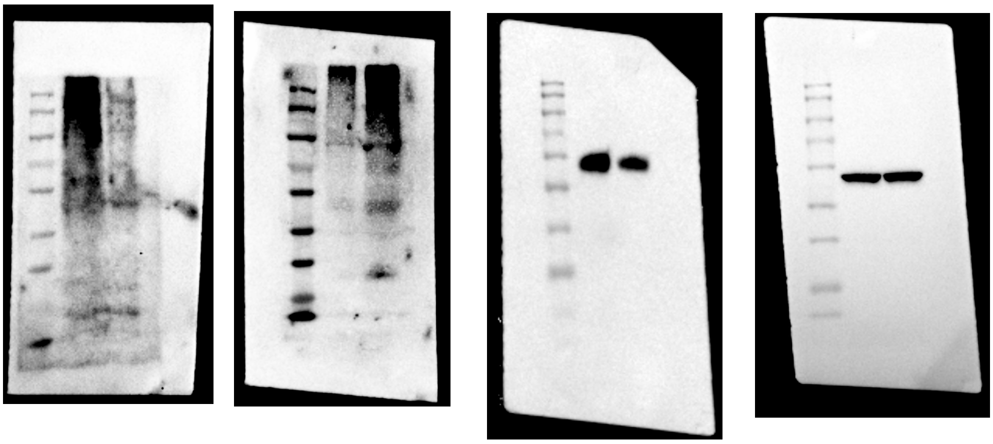

Figure 10B-1

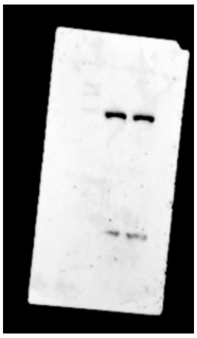

Figure 10B-2

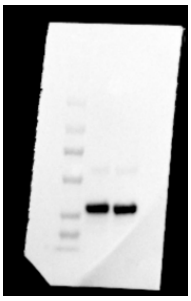

Figure *10C-1(1)*

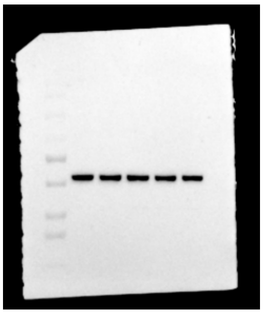

Figure *10C-1(2)*

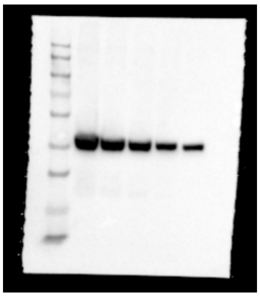

Figure *10C-2(1)*

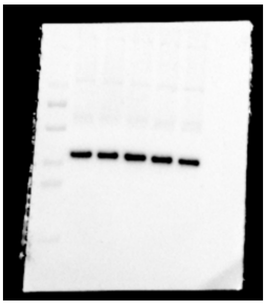

Figure *10C-2(2)*

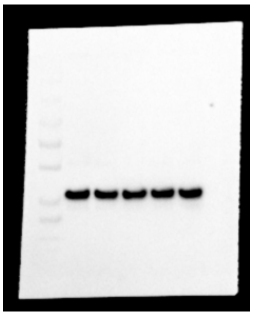

Figure 10E-1

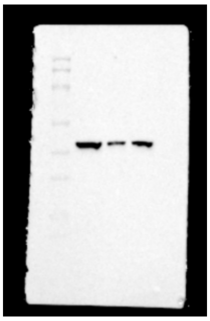

Figure 10E-2

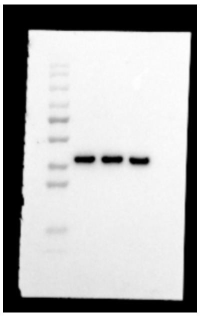

Figure 10F-1

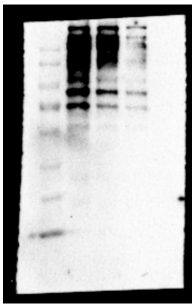

Figure 10F-2

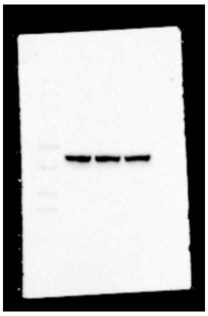

Figure 10F-3

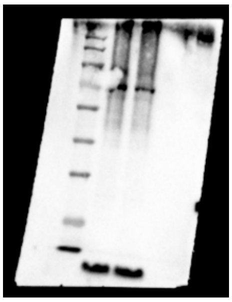

Figure 10F-4

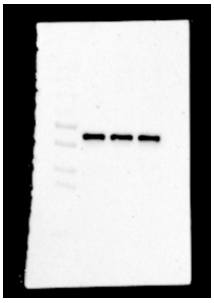

Figure 10F-5

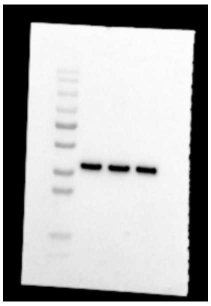

Figure 10G-1

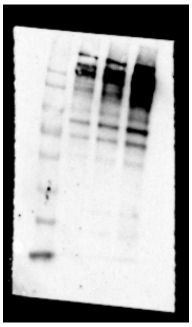

Figure 10G-2

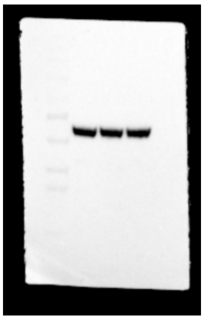

Figure 10G-3

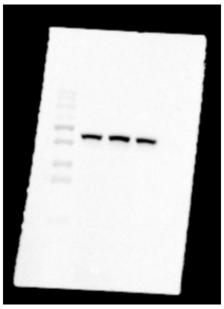

Figure 10G-4

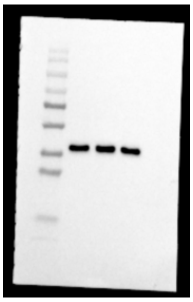

Figure S5

D1-D10

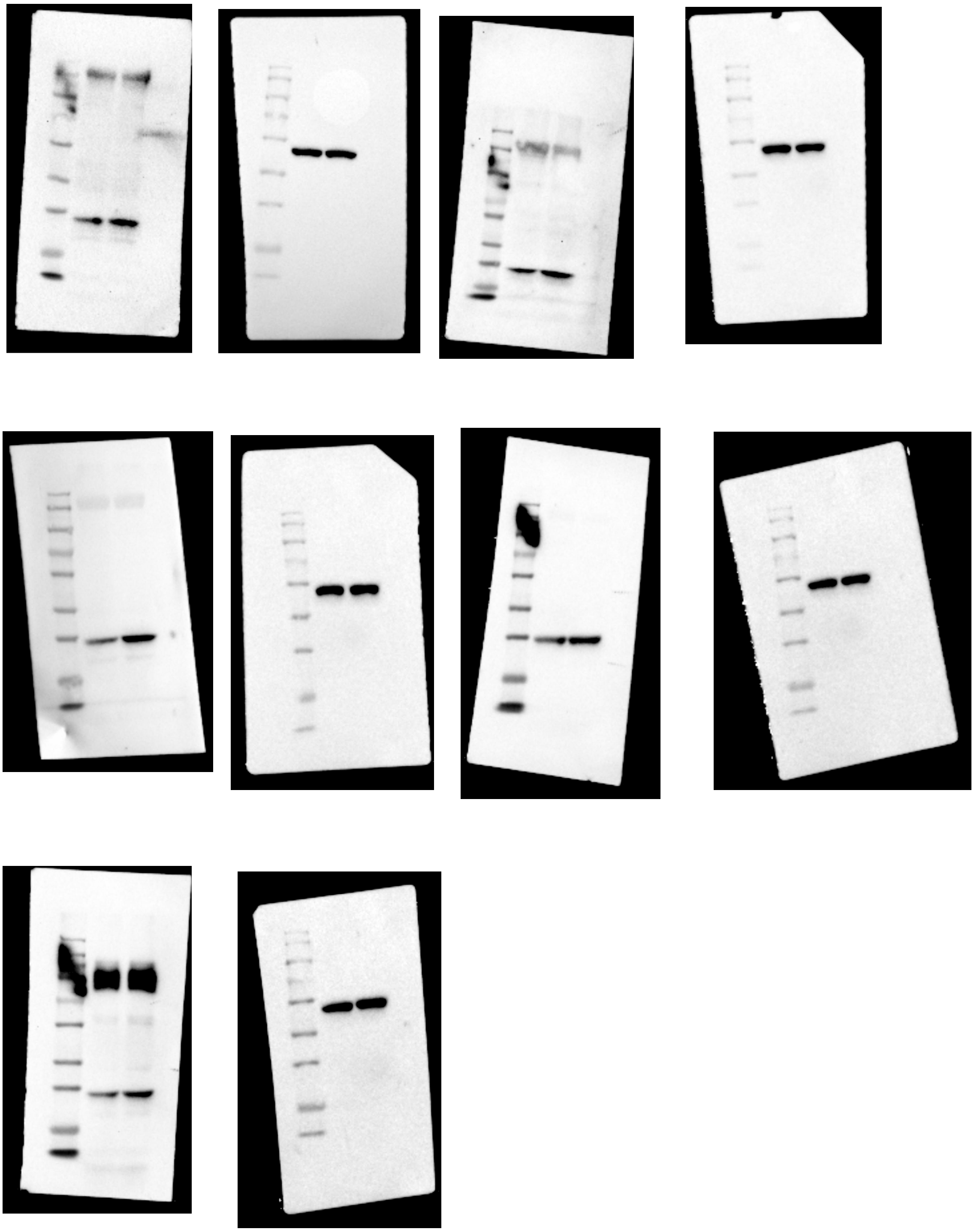

Figure S6

B1-B4

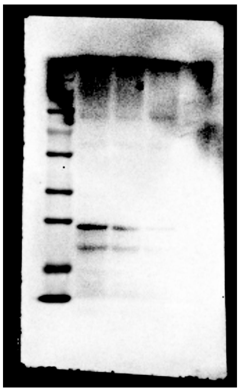

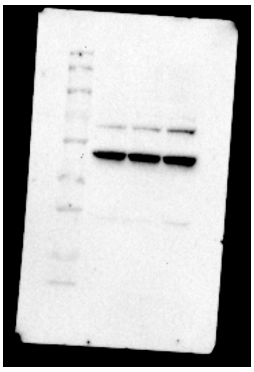

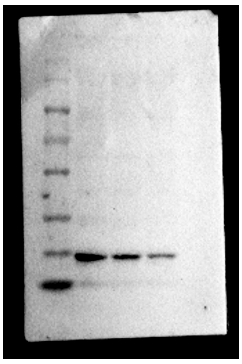

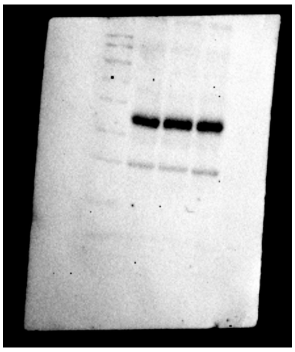

C1-C2

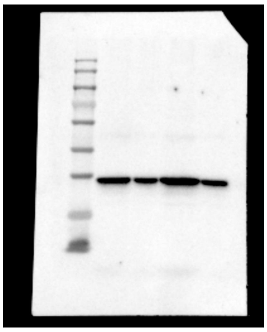

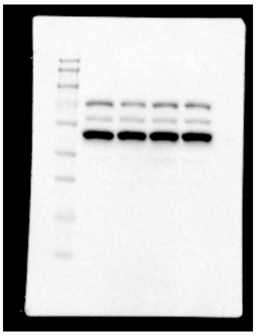

Figure S9

B2-B4

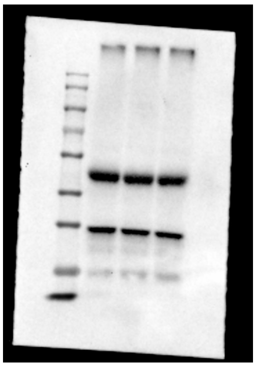

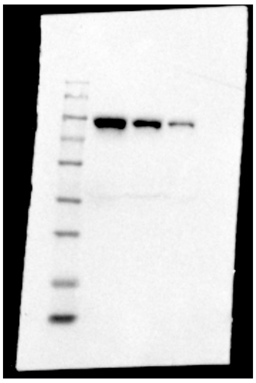

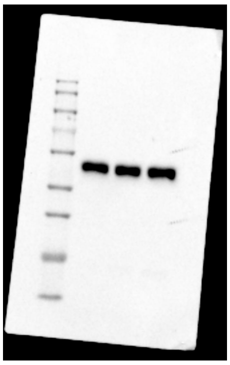

Figure S10

A1-A6

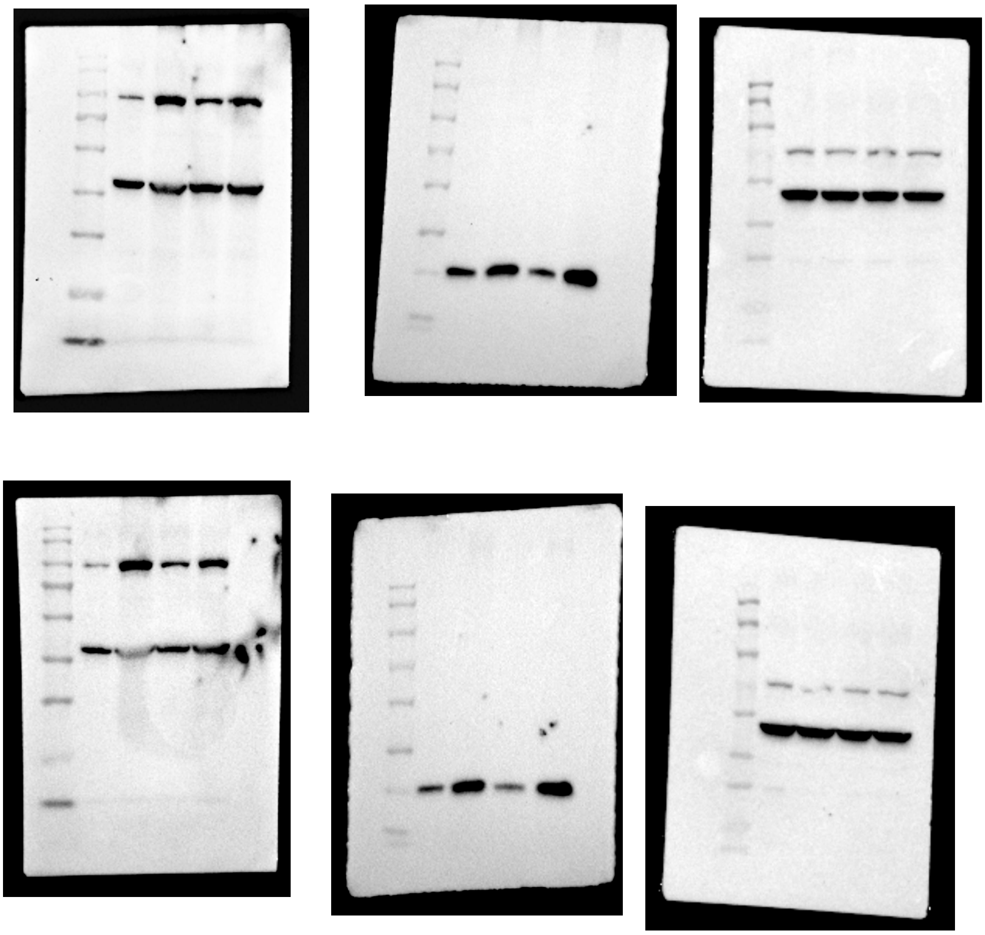

B1-B6

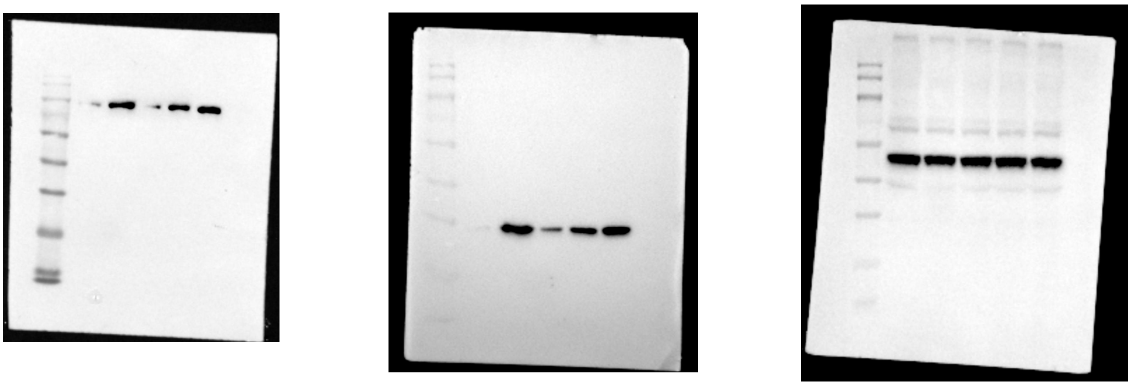

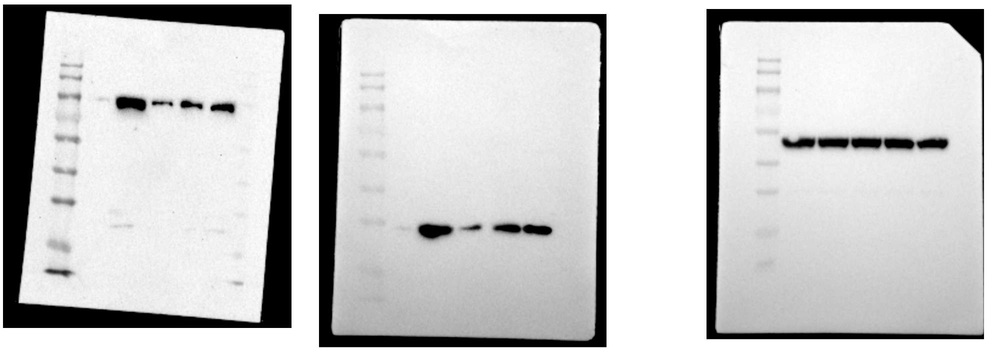

C1-C8

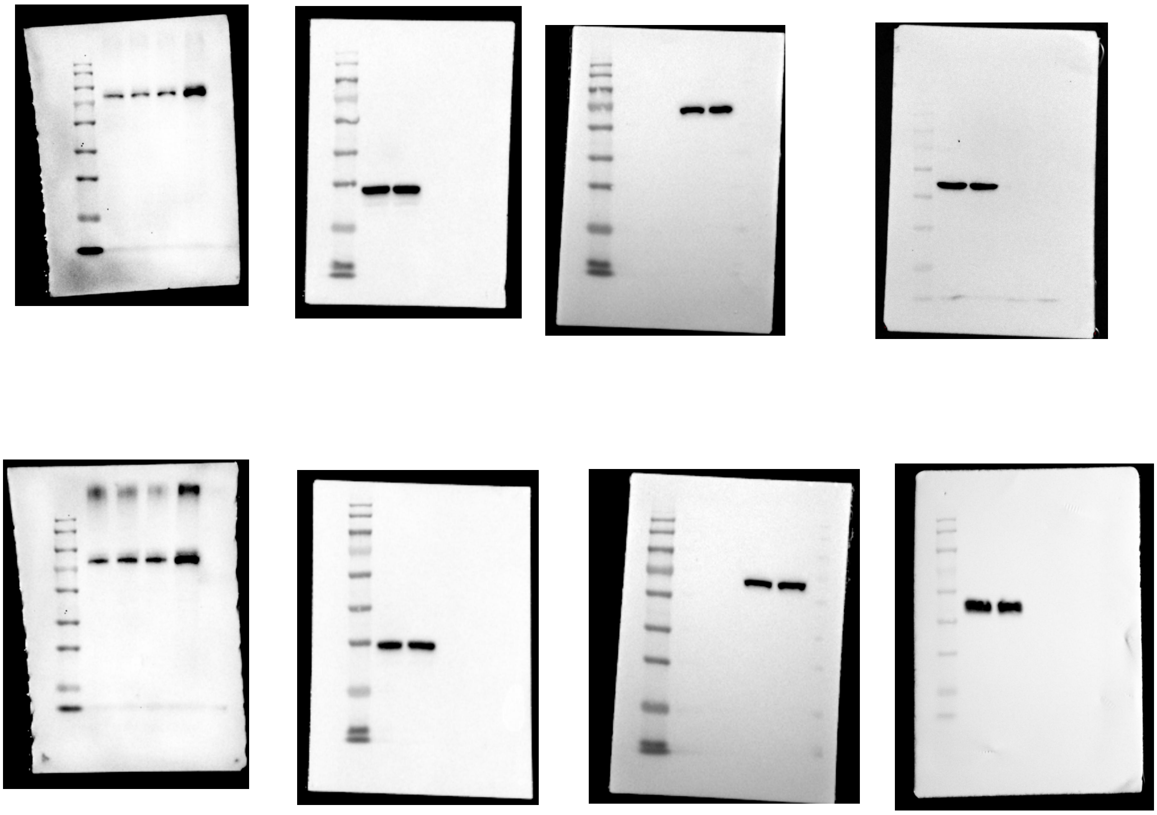

Figure S12

E1-E4

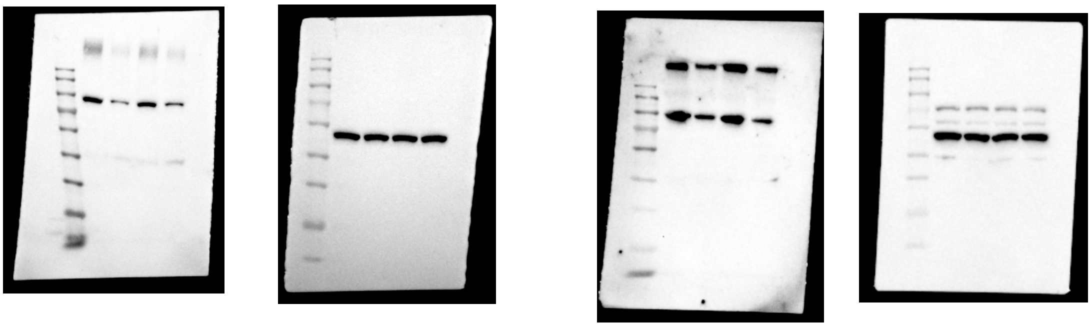

F1-F4

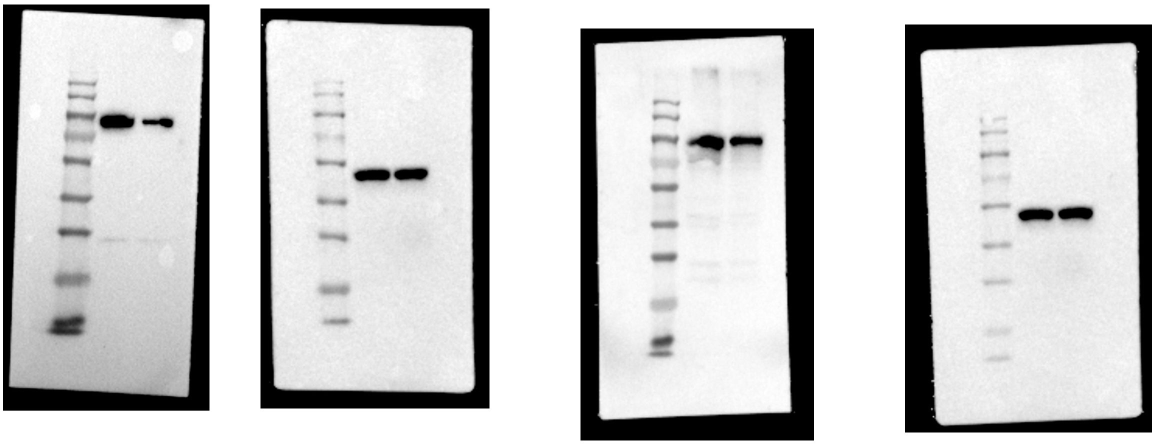

Figure S18A

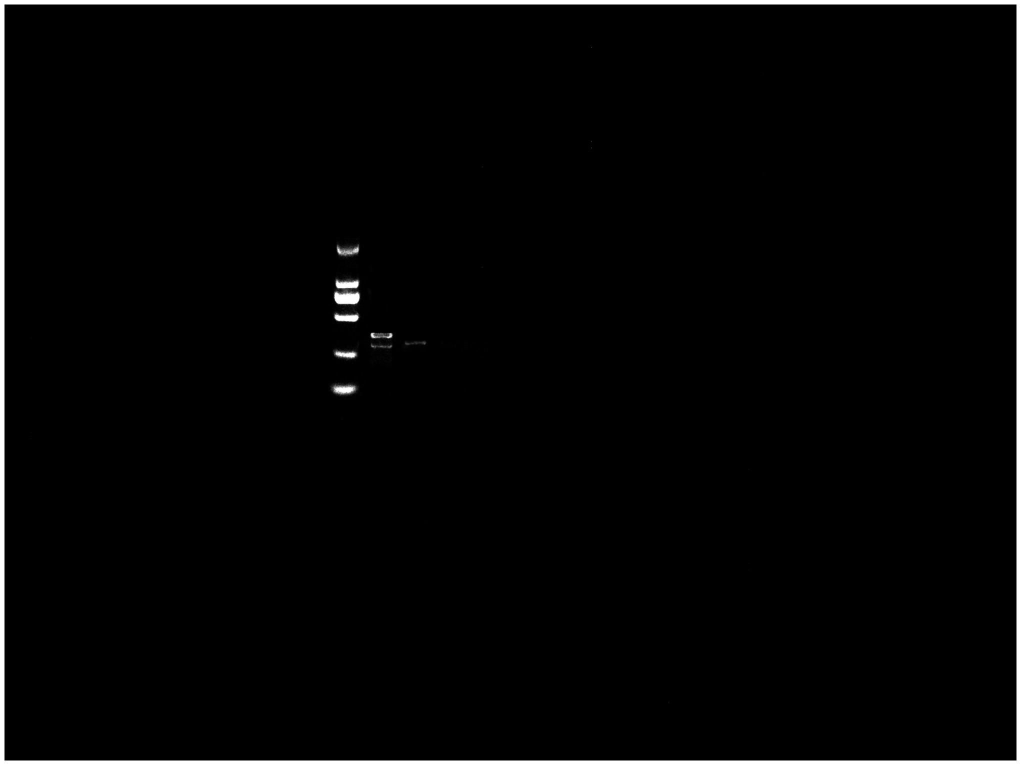

Figure S18B-1

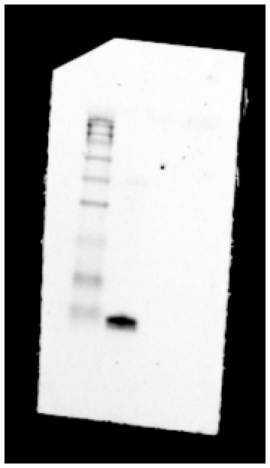

Figure S18B-2

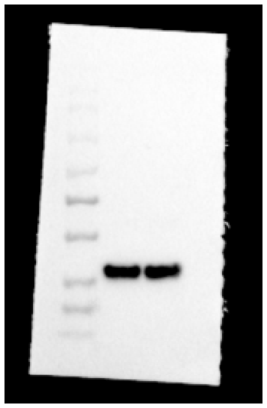

Supplement: Supplementary file 2 — Western Blot [file 41419_2026_8609_MOESM2_ESM.pdf]
