# Supplementary material for: The RASSF1C-HIF-1α axis drives macrophage lipid metabolism to promote pancreatic cancer
Source: Cell Death Dis. 2026 Mar 30;17(1):430. doi: 10.1038/s41419-026-08609-0 (PMC13156295; doi:10.1038/s41419-026-08609-0)
Supplement: Supplementary file 3 — Supplementary Tables [file 41419_2026_8609_MOESM3_ESM.docx]

**Table S1. Primer sequences for lentiviral transduction.**

| **gene** | **primer sequence (5'-3')** |
| --- | --- |
| sh-RASSF1C-1(human) | GCTTGAACAAGGACGGTTCTT |
| sh-RASSF1C-2(human) | CGGTTCTTACACAGGCTTCAT |
| sh-RASSF1C-1(mouse) | GCACTCTTTGAGCGAACTGAA |
| sh-RASSF1C-2(mouse) | GCATGCCTGAACTGCACAATT |
| sh-HIF-1α-1(human) | CCGCTGGAGACACAATCATAT |
| sh-HIF-1α-2(human) | CCAGTTATGATTGTGAAGTTA |
| sh-HIF-1α-1(mouse) | GCCACTTTGAATCAAAGAAAT |
| sh-HIF-1α-2(mouse) | GCCGCTCAATTTATGAATATT |
| sh-IRF7-1(mouse) | CCGCATAAGGTGTACGAACTT |
| sh-IRF7-2(mouse) | CTTCGACTTCAGCACTTTCTT |
| sh-NC(human) | CGCGATGTAGTTACGCTACAT |
| sh-NC(mouse) | GTATQGCTGGAGTGAAGTAAT |

**Table S2. Primer sequences for RT-qPCR.**

| **gene** | **primer sequence (5'-3')** | |
| --- | --- | --- |
| iNOS(Human) | F: AGGGACAAGCCTACCCCTC | R: CTCATCTCCCGTCAGTTGGT |
| iNOS(Mouse) | F: GTTCTCAGCCCAACAATACAAGA | R: GTGGACGGGTCGATGTCAC |
| Arg1(Human) | F: CCCTGGGGAACACTACATTTTG | R: GCCAATTCCTAGTCTGTCCACTT |
| Arg1(Mouse) | F: CTCCAAGCCAAAGTCCTTAGAG | R: GGAGCTGTCATTAGGGACATCA |
| IL‐10(Human) | F: TCTCACTCIRF7TTTGGCTCC | R: CTCCGAGACACTGGAAGGTG |
| IL‐10(Mouse) | F: CTTACTGACTGGCATGAGGATCA | R: GCAGCTCTAGGAGCATGTGG |
| TNF‐α(Human) | F: CTCTCTGCCATCAAGAGCCC | R: CAGACTCGGCAAAGTCGAGA |
| TNF‐α(Mouse) | F: CAGGCGGTGCCTATGTCTC | R: CGATCACCCCGAAGTTCAGTAG |
| RASSF1C(Human) | F: AGGACGGTTCTTACACAGGCT | R: TGGGCAGGTAAAAGGAAGTGC |
| Rassf1c(Mouse) | F: GGTACAACACGCAATCCGTCA | R: GACAACGGTAATGGCAGGTGA |
| Srebf1(Mouse) | F: TGACCCGGCTATTCCGTGA | R: CTGGGCTGAGCAATACAGTTC |
| Srebf2(Mouse) | F: GCAGCAACGGGACCATTCT | R: CCCCATGACTAAGTCCTTCAACT |
| Nr1h3(Mouse) | F: CTGATTCTGCAACGGAGTTGT | R: GACGAAGCTCTGTCGGCTC |
| Nr1h2(Mouse) | F: GCCTGGGAATGGTTCTCCTC | R: AGATGACCACGATGTAGGCAG |
| Acat2(Mouse) | F: CCCGTGGTCATCGTCTCAG | R: GGACAGGGCACCATTGAAGG |
| Mvd(Mouse) | F: ATGGCCTCAGAAAAGCCTCAG | R: TGGTCGTTTTTAGCTGGTCCT |
| Lss(Mouse) | F: CTCCAGAATGAGTTGGGTCGG | R: CGCTTTTGGTAAGTCCGTGAAA |
| Dhcr24(Mouse) | F: CGCTGCGAGTCGGAAAGTA | R: GTCACCTGACCCATAGACACC |
| GAPDH(Human) | F: GGAGCGAGATCCCTCCAAAAT | R: GGCTGTTGTCATACTTCTCATGG |
| GAPDH(Mouse) | F: AGGTCGGTGTGAACGGATTTG | R: GGGGTCGTTGATGGCAACA |

**Tabe S3. Western Blot antibody information.**

| **Target name** | **Manufacturer** | **Article number** |
| --- | --- | --- |
| RASSF1C (Human/mouse) | Bio-Techne | NBP2-03644 |
| HIF-1α (Human/mouse) | abcam | ab179483 |
| UFL1 (Human/mouse) | abcam | ab226216 |
| IRF7 (Human/mouse) | Bio-Techne | NBP2-67634 |
| UFM1(Human/mouse) | abcam | ab109305 |
| IgG(Human/mouse) | abcam | ab109489 |
| K48 ubiquitin(Human/mouse) | Cell Signaling Technology | 4289 |
| Hydroxyproline(Pro-DH)(Human/mouse) | Thermo Fisher Scientific | PA5-62366 |
| Flag(Human/mouse) | abcam | Ab205606 |
| Lamin B1 (Human/mouse) | abcam | ab229025 |
| Tubulin(Human/mouse) | abcam | ab7291 |
| GAPDH (Human/mouse) | abcam | ab8245 |
